# Contagion-preserving compression for multiscale epidemic modeling

**DOI:** 10.1126/sciadv.adz4710

**Published:** 2026-10-09

**Authors:** Leyang Xue, Zengru Di, An Zeng

**Affiliations:** ^1^ Department of Systems Science, Faculty of Arts and Sciences, Beijing Normal University, Zhuhai 519087, China.; ^2^ International Academic Center of Complex Systems, Beijing Normal University, Zhuhai 519087, China.; ^3^ School of Systems Science, Beijing Normal University, Beijing 100875, China.; ^4^ Department of Physics, Bar-Ilan University, Ramat-Gan 52900, Israel.

## Abstract

Understanding how contagion unfolds on large, heterogeneous networks is essential for predicting and controlling spreading processes, yet structural complexity often obscures the mechanisms governing transmission across scales. Here, we show that contagion is naturally organized around dense local structures that become dynamical spreading units once infection saturates within them. Building on this principle, we introduce iterative structural coarse-graining (ISCG), a framework that compresses large networks into interpretable multiscale representations while retaining the contagion dynamics of the original system. In this saturation regime, these representations reproduce macroscopic outbreak sizes, node-level infection risks, and spatiotemporal infection trajectories across scales. Beyond this regime, ISCG enables controlled trade-offs between dynamical fidelity and structural compression. The resulting multiscale representations support mechanism-driven intervention strategies, including influence maximization, immunization, and surveillance, that consistently outperform adaptive centrality–based methods. These results establish a general multiscale framework for representing, understanding, and controlling contagion in networked systems.

## INTRODUCTION

Contagion processes—ranging from infectious disease transmission (*1*–*5*) to information diffusion (*6*, *7*) and opinion formation (*8*, *9*)—are shaped by the structure of the underlying network. Accurate modeling of these dynamics is critical for prediction, control, and intervention across public health, social, and technological systems. A central challenge lies not only in predicting macroscopic outcomes such as outbreak sizes or critical thresholds but also in understanding how contagion emerges from interactions across structural scales. Spreading is driven by microscopic contacts, organized by mesoscale network structures, and ultimately manifested at the system level. Capturing this multiscale organization is therefore essential for revealing how network structure shapes contagion across scales (*10*, *11*).

The scale and heterogeneity of real-world networks make this challenge particularly acute. Large-scale simulations can reproduce contagion dynamics but often obscure the structural origins of spreading and provide limited mechanistic insight. Analytical approaches, including mean-field approximations (*4*, *12*, *13*), generating-function techniques (*14*, *15*), and message-passing methods (*16*–*18*), have yielded important theoretical understanding yet rely on simplifying assumptions such as homogeneous mixing, weak correlations, or locally treelike structure. As a result, they struggle to capture the structure-driven and mesoscale contagion in realistic networks. Moreover, even with advances in simulation algorithms, repeated simulations on large networks remain costly and do not, by themselves, explain how microscopic interactions aggregate into collective behavior. These limitations motivate the need for intermediate representations that connect microscopic interactions with macroscopic contagion patterns.

One natural route toward such intermediate representations is network coarse-graining, which reduces structural complexity while retaining information relevant to dynamical processes. Existing approaches to network simplification and dynamics-aware reduction can be broadly grouped into three complementary classes, distinguished by what they preserve. First, structure-driven reductions, including clustering, renormalization, and edge-based backbone extraction (*19*–*27*), aim to simplify topology or highlight salient pathways. Although often computationally efficient and sometimes preserving macroscopic outcomes, they typically pruning edges and yield subgraphs rather than reduced state-space models. Second, model-based and information-theoretic approaches, such as minimum-description-length formulations and multilayer reducibility (*28*, *29*), provide principled compressed descriptions but generally do not produce explicit coarse-grained networks (CGNs) on which contagion dynamics can be directly evolved. Third, dynamics-aware methods, including spectral coarse-graining, low-dimensional embeddings, and chronology compression in temporal or multilayer networks (*30*–*36*), target the preservation of specific operators, modes, or temporal orderings but are typically tailored to particular linear dynamics and offer limited insight into the mesoscale structures that organize nonlinear contagion. Together, these approaches reveal a common gap: the lack of a general coarse-graining framework that identifies dynamically coherent spreading units, preserves contagion dynamics beyond macroscopic observables, and provides an interpretable multiscale representation of spreading.

Here, we introduce iterative structural coarse-graining (ISCG), a framework that preserves contagion dynamics while yielding an interpretable multiscale representation of network structure. ISCG is based on a simple observation that, once infection saturates within a dense local structure, the subsequent spread is governed primarily by transmission between such structures rather than by the detailed dynamics of individual nodes. Accordingly, ISCG identifies densely connected *k*-cliques and aggregates them into supernodes that act as dynamically coherent mesoscale spreading units. This construction is supported by a theoretical criterion: Above a matching transmission rate ${\hat{\beta }}_{k}$, infection within a *k*-clique becomes effectively inevitable, allowing the clique to be represented as a single dynamical unit without loss of contagion fidelity. The framework therefore establishes an explicit connection between transmission dynamics and structural coarse-graining, clarifying when mesoscale aggregation is dynamically justified.

Beyond providing structural compression, ISCG offers an interpretable multiscale representation of contagion. Rather than reducing a network to aggregate equations or low-dimensional descriptors (*37*), it preserves an explicit CGN in which supernodes represent effective spreading units and superedges describe transmission between them. The framework naturally extends from exact to controlled approximate coarse-graining, providing a continuum of structural resolutions that can be selected according to the desired balance between dynamical fidelity and structural simplicity. ISCG therefore links microscopic interactions, mesoscale organization, and macroscopic epidemic behavior within a common representation, providing a structural basis for analyzing and interpreting contagion across scales.

We evaluate ISCG on diverse empirical networks spanning social, biological, technological, infrastructure, and large-scale human mobility systems. Across these networks, the framework preserves not only aggregate epidemic outcomes but also node-level infection risks and the spatiotemporal organization of contagion over a broad range of transmission regimes. The resulting multiscale representations further support practical applications including influence maximization (IM), targeted immunization, and sentinel surveillance (SS) by identifying the mesoscale structures most relevant to contagion. These results demonstrate that ISCG provides a general framework for representing, interpreting, and controlling contagion processes across multiple structural scales.

## RESULTS

### Contagion model

We adopt the classical susceptible-infected-recovered (SIR) model to simulate contagion dynamics on complex networks. Theoretically, the SIR model provides a foundational framework for studying the spread of epidemics, information, and innovation (*1*, *2*); practically, it supports applications ranging from epidemic control (*38*, *39*) to viral marketing (*40*, *41*).

In the SIR model, each node exists in one of three states: susceptible (*S*), infected (*I*), or recovered (*R*). Susceptible nodes may become infected through contact with infected neighbors; infected nodes transmit the contagion with probability β and recover with probability μ, transitioning to the *R* state and becoming permanently immune. We consider a discrete-time implementation in which all nodes are initially susceptible, except for a small set of seed nodes initialized as infected. The process evolves iteratively until no infected nodes remain.

To characterize the system-level outcome, we define the final infection density as $\rho =\frac{1}{N}\sum_{i=1}^{N}s\left(v_{i}\right)$, where $s\left(v_{i}\right)=1$ if node $v_{i}$ is ever infected and $s\left(v_{i}\right)=0$ otherwise. This macroscopic quantity serves as an order parameter for the system. Throughout this study, we set the recovery probability to μ=1, which renders the discrete-time SIR process exactly equivalent to bond percolation (*14*).

### ISCG framework

The ISCG framework reduces large-scale networks into compact representations while preserving the contagion dynamics of the original system. Achieving this requires overcoming two key challenges: (i) reproducing the final outbreak size observed in the original network through simulations on the CGN with identical initial conditions and (ii) ensuring that the temporal and structural organization of the spreading process on the CGN remains dynamically consistent with that of the full-resolution system.

ISCG addresses these challenges by iteratively merging dense, locally redundant subgraphs, formalized here as maximal cliques—complete subgraphs in which all nodes are mutually connected—into supernodes. Such structures provide multiple redundant transmission pathways, so that once infection enters a clique, intraclique spreading is effectively fast and well mixed. As a result, each supernode acts as a dynamically coherent surrogate for its constituent subgraph, capturing its internal transmission behavior at a coarser scale. In the coarse-grained model, we therefore assume that, when a supernode becomes infected, all nodes it represents in the original network are infected. This mapping preserves the correspondence between coarse-grained and microscopic states. By summing the weights of infected supernodes in a given contagion configuration M, we obtain an accurate estimate of the final outbreak size in the original network. This construction preserves both macroscopic observables (e.g., outbreak size) and microscopic consistency (e.g., node-level infection coverage and infection ordering) while providing an interpretable multiscale representation of the spreading process.

#### ISCG method

We propose an iterative coarse-graining method that systematically reduces network size while preserving key dynamical properties relevant to contagion processes. Let G(V,E) denote an undirected network with N=∣V∣ nodes and ∣E∣ edges. Each node and edge is initially assigned a unit weight, yielding a weighted representation $G\left(V_{w},E_{w}\right)$ defined as

$$\begin{array}{c} V_{w}=\left\{v_{i}^{w}|v_{i}^{w}=1,v_{i}\in V\right\}\\ E_{w}=\left\{e_{\mathrm{ij}}^{w}|e_{\mathrm{ij}}^{w}=1,e_{\mathrm{ij}}\in E\right\} \end{array}$$ (1)

The coarse-graining procedure is defined by an operator F that maps a network *G* to a CGN $G^{\prime }=F\left(G\right)$. At each iteration, maximal cliques in the current network are identified and contracted into supernodes. When a node participates in multiple maximal cliques, one clique is selected at random for contraction. Edges between supernodes, or between a supernode and an individual node, are represented as superlinks. The weights of supernodes and superlinks are updated as the sums of the weights of their constituent nodes and edges, respectively (Fig. 1A). This procedure is applied iteratively until the network is reduced to a single node or until a prescribed coarse-graining level is reached. During each iteration, all external links of a contracted clique are reconnected to the corresponding supernode, which may induce additional cliques in the resulting CGN. Through this iterative process, ISCG achieves substantial structural compression while preserving the connectivity patterns that govern contagion dynamics.

**Fig. 1.**
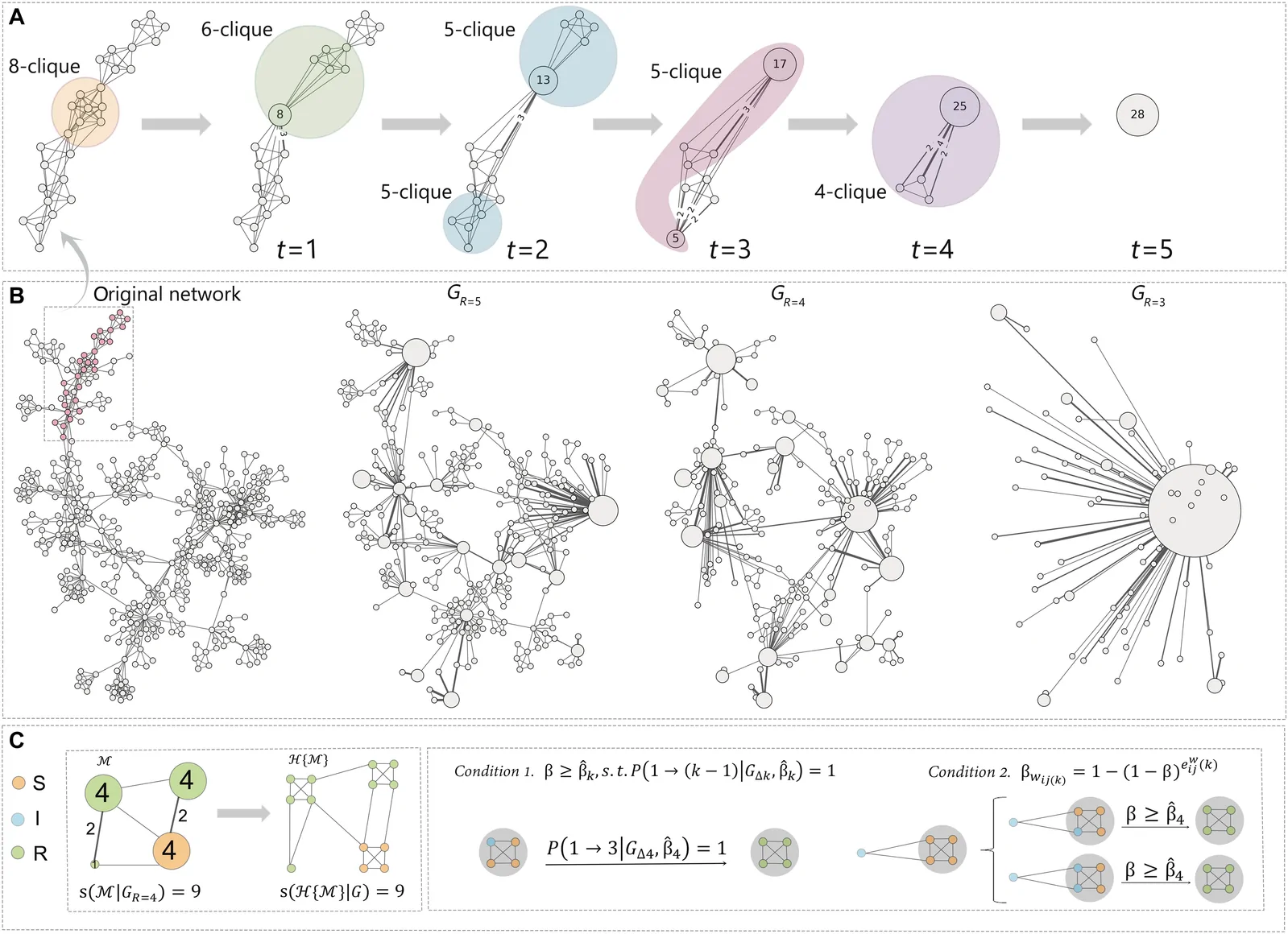
Illustration of the ISCG framework for preserving contagion dynamics. (**A**) Schematic illustration of the coarse-graining process. Maximal cliques in the network (e.g., orange circle) are merged into supernodes. Each supernode inherits all links of the merged clique, and its node weight equals the sum of the constituent node weights (initially 1 per node). Multiple links to the same neighbor are replaced by a single weighted edge whose weight equals the sum of the original links. This iterative procedure generates a sequence of weighted networks (e.g., t=1, second column) and continues until the network collapses into a single node. (**B**) Construction of *k*-clique CGNs. ISCG iteratively simplifies the original network (e.g., a coauthorship network) until all remaining cliques have size smaller than *k*, yielding a *k*-clique CGN ($G_{R=k}$). Examples are shown for k=5, 4, and 3. Node size represents the node weight, and edge thickness indicates the aggregated edge weight. (**C**) Preservation of the final outbreak size *s* under SIR dynamics. For a given contagion configuration M on $G_{R=4}$, all constituent nodes of an infected supernode are assumed to be infected. This induces a configuration H{M} on the original network, where *s* is obtained by summing nodes in the recovered (*R*) state. Two conditions ensure preservation of *s*: (i) $\beta \geq {\hat{\beta }}_{k}$, where ${\hat{\beta }}_{k}$ is the minimum transmission probability required to infect all nodes within a *k*-clique ($G_{\Delta_{k}}$), and (ii) $\beta_{w_{\mathrm{ij}}\left(k\right)}=1-{\left(1-\beta \right)}^{e_{\mathrm{ij}}^{w}\left(k\right)}$, where $\beta_{w_{\mathrm{ij}}\left(k\right)}$ is the effective transmission probability across a weighted edge $e_{\mathrm{ij}}^{w}\left(k\right)$ in $G_{R=k}$.

To formalize the construction, we define the *k*-clique CGN, denoted $G_{R=k}$, as the network obtained by iteratively applying F until all remaining cliques have size smaller than *k*

$$G_{R=k}=F^{\left(t_{k}\right)}\left(G\right),\;1<k\leq K$$ (2)

where K is the size of the largest clique in the original network and $t_{k}$ is the number of iterations required to reach $G_{R=k}$ (Fig. 1B). In the limiting case k=2, the procedure yields a single supernode whose weight equals the total number of nodes in *G*.

Although the coarse-graining procedure is formulated in terms of maximal cliques, this construction primarily serves to elucidate the multiscale and self-similar nature of the coarse-graining process. In practice, for a given *k*-clique coarse-graining scale, the dynamical fidelity of ISCG does not depend on a specific clique contraction order and does not require explicit identification of the largest cliques. Instead, the framework supports multiple local and iterative contraction strategies that yield approximately equivalent coarse-grained representations. Consequently, the computational cost is controlled by the number and sizes of cliques encountered during reduction and empirically scales polynomially with network size, $T∼N^{\alpha }$ with α≈2.0 to 2.2. Further complexity analysis and implementation details are provided in section S2.4 and figs. S3 to S6.

#### Conditions for preserving contagion dynamics

To ensure that contagion dynamics on the *k*-clique CGN faithfully reproduce those of the original network, two conditions must be satisfied. Together, they guarantee preservation of both macroscopic observables (e.g., final outbreak size) and microscopic behavior (e.g., node-level infection coverage and ordering) across scales (Fig. 1C).

##### Condition 1: Minimum transmission probability for intraclique contagion

To represent the internal dynamics of a *k*-clique by a single supernode, the transmission probability β must exceed a critical threshold ${\hat{\beta }}_{k}$, such that infection initiated at any node spreads to all other nodes within the clique. We define this threshold as

$$\beta \geq {\hat{\beta }}_{k},\;{\hat{\beta }}_{k}=\text{min}\;\underset{\beta }{\text{arg}\;\text{max}}\Lambda_{1\to \left(k-1\right)}^{k}\left(\beta \right)$$ (3)

where $\Lambda_{1\to \left(k-1\right)}^{k}\left(\beta \right)$ denotes the probability that a single infected node transmits the contagion to the remaining (*k* − 1) nodes of a *k*-clique. Explicit expressions for $\Lambda_{1\to \left(k-1\right)}^{k}\left(\beta \right)$ are derived in Eqs. 5 and 6. When this condition is satisfied, each supernode accurately represents complete intraclique contagion in the original network (Proposition 1).

##### Condition 2: Effective transmission across superlinks

To preserve transmission between supernodes, the effective infection probability $\beta_{w_{\mathrm{ij}}\left(k\right)}$ associated with a weighted superlink must match the cumulative probability of transmission along the corresponding edges in the original network. This yields

$$\beta_{w_{\mathrm{ij}}\left(k\right)}=1-{\left(1-\beta \right)}^{e_{\mathrm{ij}}^{w}\left(k\right)}$$ (4)

where $e_{\mathrm{ij}}^{w}\left(k\right)$ denotes the number of original edges aggregated into the superlink between nodes *i* and *j* in $G_{R=k}$. This expression ensures consistent interunit transmission whether interactions occur between individual nodes, supernodes, or mixed structures (Proposition 2).

Together, Condition 1 guarantees dynamical fidelity within each supernode, whereas Condition 2 ensures accurate propagation between supernodes. These two principles form the theoretical basis for preserving contagion dynamics on *k*-clique CGNs. Additional derivations and technical details are provided in the Methods section.

### Complete contagion in *k*-cliques

The minimum transmission probability ${\hat{\beta }}_{k}$ characterizes the onset of effectively complete contagion within a *k*-clique and is central to the fidelity of the ISCG framework. ${\hat{\beta }}_{k}$ is defined in terms of the eventual outcome of the contagion process, rather than infection occurring in a single transmission step. Specifically, we consider the probability $\Lambda_{1\to \left(k-1\right)}^{k}\left(\beta \right)$ that a single initially infected node ultimately leads to infection of all remaining *k* − 1 nodes in a symmetric *k*-clique, allowing for arbitrarily many transmission steps until the process terminates. Throughout this analysis, we adopt the SIR model with deterministic recovery (μ=1), which allows for a transparent analytical treatment of intraclique contagion.

In a fully connected *k*-clique (k≥2; Fig. 2A), infection can propagate through multiple redundant pathways. Although there is always a finite probability that the initial seed fails to infect any neighbor in the first step, once infection persists beyond this initial stage, newly infected nodes can continue to transmit the contagion to remaining susceptible nodes through repeated interactions. As a consequence, there exists a finite threshold ${\hat{\beta }}_{k}$ such that, for $\beta \geq {\hat{\beta }}_{k}$, the absorbing outcome in which all *k* nodes become infected occurs with probability one, i.e., $\Lambda_{1\to \left(k-1\right)}^{k}\left(\beta \right)=1$. The probability $\Lambda_{1\to \left(k-1\right)}^{k}\left(\beta \right)$ satisfies the recursive relation

$$\Lambda_{1\to \left(k-1\right)}^{k}\left(\beta \right)=\sum_{m=1}^{k-1}\left(\begin{array}{c} k-1\\ m \end{array}\right)\beta^{m}{\left(1-\beta \right)}^{k-1-m}\Lambda_{m\to \left(k-1-m\right)}^{k-1}\left(\beta \right)$$ (5)

where *m* denotes the number of nodes directly infected by the seed in the first time step, and $\Lambda_{m\to \left(k-1-m\right)}^{k-1}\left(\beta \right)$ is the probability that these *m* infected nodes subsequently infect the remaining (k−1−m) nodes.

**Fig. 2.**
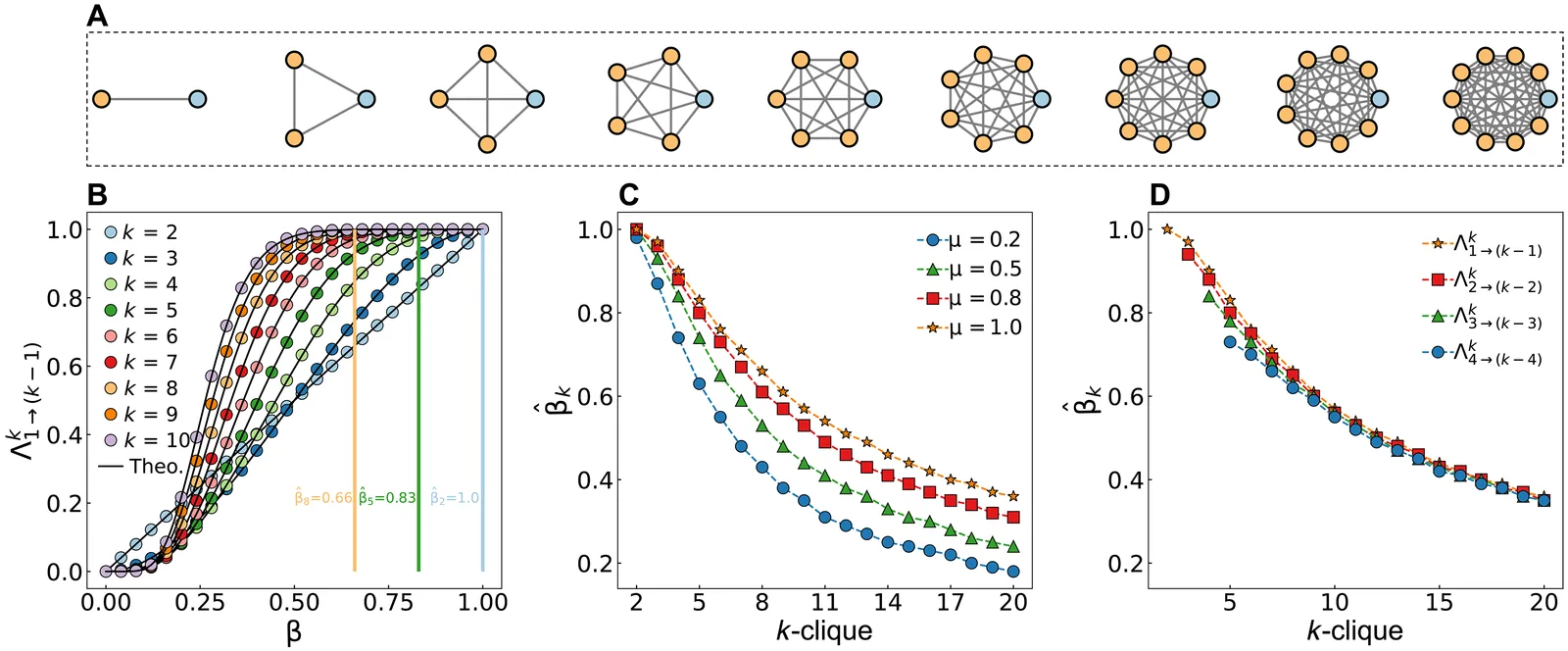
Minimum transmission probability required to preserve contagion behavior in *k*-clique CGNs. (**A**) Schematic illustration of a single infected seed node transmitting infection to all other nodes within a *k*-clique (k=2 to 10). Susceptible nodes are shown in orange, and the initial infected node is shown in blue. (**B**) Probability $\Lambda_{1\to \left(k-1\right)}^{k}$ that a single seed fully infects a *k*-clique as a function of the transmission probability β. Circle markers denote numerical results, whereas solid curves correspond to theoretical predictions from Eqs. 5 and 6. Vertical lines indicate the minimum transmission probability ${\hat{\beta }}_{k}$ at which $\Lambda_{1\to \left(k-1\right)}^{k}=1$. (**C**) Dependence of ${\hat{\beta }}_{k}$ on the clique size *k* for different recovery rates μ. Larger cliques require lower transmission probabilities to ensure full infection. (**D**) Effect of multiple initial seeds on ${\hat{\beta }}_{k}$ for different clique sizes at μ=1. As *k* increases, the difference between single-seed and multiseed thresholds diminishes, indicating reduced sensitivity to initial conditions.

To evaluate $\Lambda_{m\to \left(k-1-m\right)}^{k-1}\left(\beta \right)$, we enumerate all configurations in which contagion fails to reach a subset of nodes, yielding

$$\begin{array}{c} \Lambda_{m\to \left(k-1-m\right)}^{k-1}\left(\beta \right)=1-\sum_{n=0}^{k-2-m}\left(\begin{array}{c} k-1-m\\ n \end{array}\right){\left(1-\beta \right)}^{m\left(k-1-m-n\right)}\\ \cdot {\left(1-\beta \right)}^{n\left(k-1-m-n\right)}\Lambda_{m\to n}^{m+n}\left(\beta \right) \end{array}$$ (6)

where *n* is the number of susceptible nodes that become infected in each configuration. By definition, $\Lambda_{m\to 0}^{m}\left(\beta \right)=1$ for all *m* and β as no further infections are required. This recursive structure enables exact evaluation of $\Lambda_{1\to \left(k-1\right)}^{k}\left(\beta \right)$ via iterative computation. For example, substituting k=Z and m=1 into Eq. 6 yields

$$\Lambda_{1\to \left(Z-2\right)}^{Z-1}\left(\beta \right)=\Lambda_{1\to \left[\left(Z-1\right)-1\right]}^{\left(Z-1\right)}\left(\beta \right)$$ (7)

which is consistent with Eq. 5 evaluated at k=Z−1, highlighting the self-similar structure of the recurrence. Solving Eqs. 5 and 6 recursively allows us to determine the threshold ${\hat{\beta }}_{k}$ above which contagion within a *k*-clique becomes effectively complete. A full derivation, including explicit examples for small values of *k* and general recursive formulas, is provided in section S2.

We validate the analytical results by comparing the theoretical values of $\Lambda_{1\to \left(k-1\right)}^{k}\left(\beta \right)$ with numerical simulations across a range of clique sizes (Fig. 2B). The strong agreement confirms the accuracy of the recursive formulation and the correctness of the derived threshold ${\hat{\beta }}_{k}$. The threshold ${\hat{\beta }}_{k}$ provides several key insights into contagion dynamics within *k*-cliques. First, ${\hat{\beta }}_{k}$ decreases monotonically with increasing clique size *k* (Fig. 2B), indicating that the transmission rate required for effectively complete contagion becomes lower in larger cliques. As a consequence, if complete contagion is achieved within a clique of size *k* at $\beta ={\hat{\beta }}_{k}$, it will also be achieved in any larger clique at the same transmission rate. Second, ${\hat{\beta }}_{k}$ reveals a fundamental trade-off between structural reduction and dynamical fidelity. Smaller *k*-clique CGNs enable stronger compression but require higher transmission probabilities to ensure effective intraclique contagion, whereas larger *k* values relax this requirement, achieving higher dynamical fidelity at the expense of reduced compression (Fig. 2C). Third, introducing multiple initial seeds within a clique lowers the transmission probability required for complete contagion compared to the single-seed case (Fig. 2D). This effect diminishes as *k* increases, indicating that the influence of seeding multiplicity becomes negligible in large cliques. Nevertheless, the single-seed threshold ${\hat{\beta }}_{k}$ remains the most stringent condition for guaranteeing effective intraclique contagion and, consequently, for preserving supernode activation dynamics under coarse-graining. These results provide a quantitative foundation for the validity of clique-based coarse-graining and clarify how the balance between structural compression and dynamical fidelity is controlled by the transmission rate and the chosen coarse-graining scale.

### Structural compression

ISCG reduces network complexity by iteratively contracting dense local structures into supernodes while preserving the large-scale organization of the network. As representative examples, we first consider four scientific collaboration networks—GrQc, CondMat, HepPh, and NetScience—constructed as one-mode coauthorship projections of underlying author-paper bipartite systems (*42*). All analyses are performed on the resulting unipartite networks, and ISCG does not use information from the underlying bipartite structure.

The evolution of clique structure during coarse-graining reveals the multiscale nature of the compression process. Figure 3A shows that clique sizes follow an approximately power-law distribution, indicating that small cliques dominate the local organization of these networks. Consequently, the early stages of coarse-graining produce rapid structural reduction. As coarse-graining proceeds, however, repeatedly contracting small cliques also generates additional cliques, producing the extended plateaus observed in Fig. 3B. Figure 3C further shows that the newly generated cliques also follow an approximately power-law size distribution, indicating that this self-similar organization is maintained throughout the coarse-graining process. These observations show that network compression unfolds across multiple structural scales rather than through a single dominant aggregation step.

**Fig. 3.**
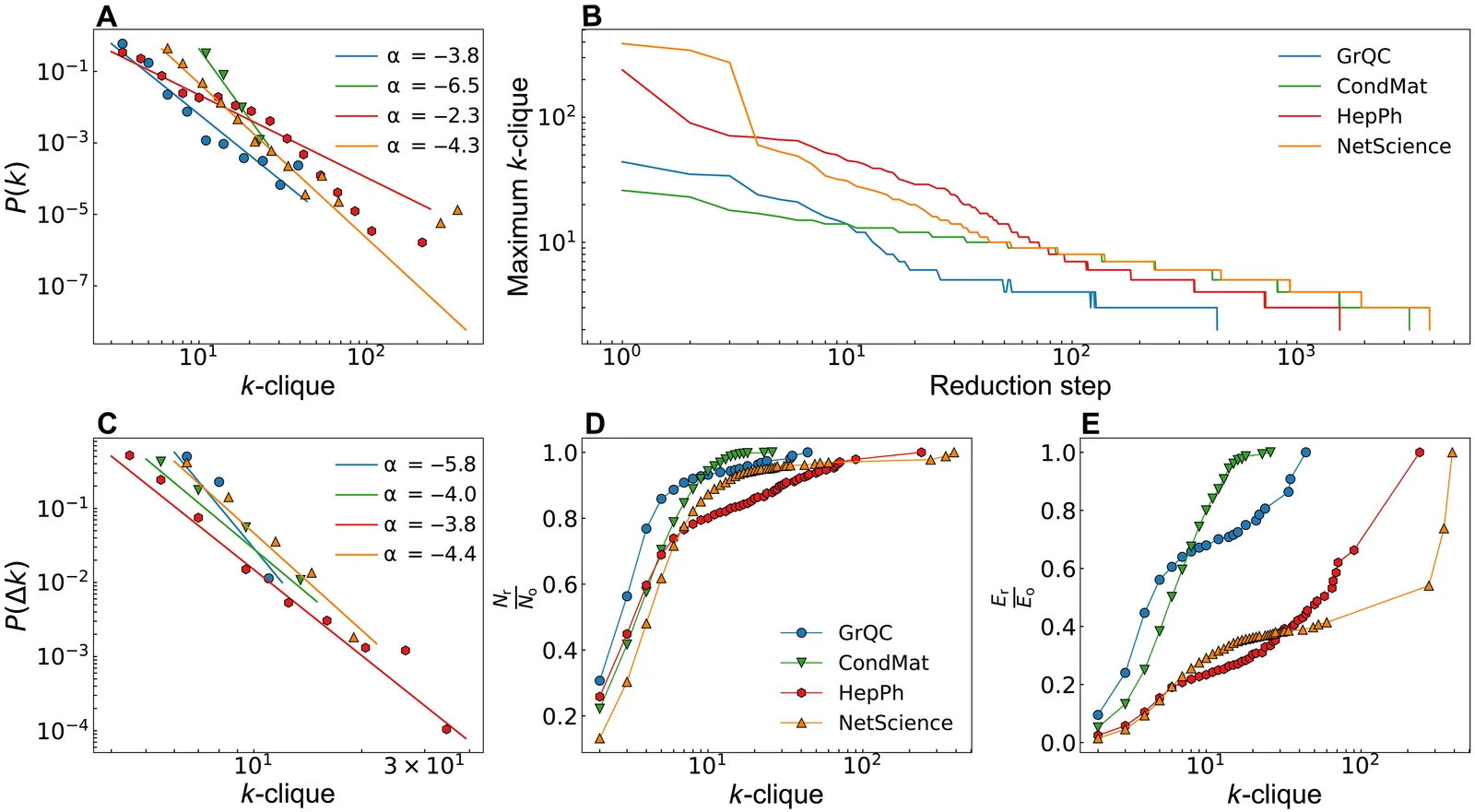
Network reduction performance of *k*-clique CGNs across four real-world networks. (**A**) Distribution of clique sizes in the original networks, exhibiting a power-law form $P\left(k\right)∼k^{-\alpha }$. The exponent α is estimated via maximum-likelihood fitting. (**B**) Evolution of the largest clique size across successive coarse-graining iterations. As *k* decreases, merging small cliques frequently generates additional *k*-cliques, leading to extended plateaus in the reduction process. (**C**) Size distribution of newly formed cliques introduced during coarse-graining (excluding original cliques). These distributions also follow a power-law form, indicating structural self-similarity across reduction scales. (**D**) Fraction of nodes retained in the *k*-clique CGNs relative to the original network. Node reduction accelerates as *k* decreases. (**E**) Fraction of links retained in the *k*-clique CGNs relative to the original network. Link reduction is more pronounced than node reduction, yielding substantial structural simplification.

The multiscale coarse-graining process results in substantial structural reduction. Figure 3 (D and E) quantifies the retained node and edge fractions ($N_{r}/N_{0}$ and $E_{r}/E_{0}$) as functions of the clique threshold *k*. As progressively smaller cliques are contracted, both quantities decrease monotonically, demonstrating progressively stronger compression. At k=5, more than half of the original edges are removed in most collaboration networks, whereas larger systems such as NetScience exhibit even greater reductions. Comparable levels of structural compression are observed across biological, social, technological, infrastructure, transportation, and mobility networks (fig. S7), indicating that the structural reduction achieved by ISCG is not specific to collaboration networks.

Despite this substantial compression, the essential structural organization of the original networks is largely retained. Path-length distributions and local clustering statistics are approximately preserved across coarse-graining scales after appropriate rescaling (fig. S8). This preservation of multiscale structural organization provides the structural basis for the dynamic fidelity demonstrated in the following section. More generally, the ISCG framework is not restricted to clique-based coarse-graining. For example, replacing cliques with *k*-plexes yields comparable structural compression (fig. S9), demonstrating that the framework is not tied to a specific structural motif and can be generalized naturally to alternative dense-subgraph definitions.

### Dynamic fidelity and trade-offs

A central goal of ISCG is to reduce network structure while retaining the contagion behavior of the original system. This requires balancing two linked properties: dynamic fidelity and structural compression. We therefore first examine temporal contagion dynamics in representative collaboration networks and then characterize final epidemic outcomes together with the exact- and approximate-preservation regimes. The resulting compression-accuracy trade-off is quantified across the complete set of empirical networks.

#### Preservation of temporal contagion dynamics

Figure 4 evaluates the temporal contagion dynamics for the original networks and their corresponding k=10 clique CGNs across four representative collaboration networks under three transmission regimes: low ($\frac{{\hat{\beta }}_{10}}{3}$), intermediate ($\frac{2{\hat{\beta }}_{10}}{3}$), and the theoretically matched transmission rate (${\hat{\beta }}_{10}$). The first two rows compare node-level infection probabilities and mean infection times (Fig. 4, A to H). Because coarse-graining shortens effective transmission paths, it systematically advances the apparent spreading time. We therefore compare temporal observables after applying a single global temporal shift, ΔT, which can be predicted directly from the reduction in average shortest-path length (fig. S10). After this correction, mean infection times remain highly correlated across all four networks and transmission regimes, whereas node-level infection probabilities exhibit near-perfect agreement. These observations indicate that the CGNs preserve both the temporal organization of contagion and the heterogeneous infection risks of individual nodes. The same agreement extends to population-level epidemic dynamics. Both the transient infected fraction *I*(*t*) and the cumulative outbreak trajectory ρ(t) remain closely matched across all transmission regimes (Fig. 4, I to P), with consistently small root mean square errors (RMSEs). These observations show that ISCG preserves not only which nodes are most likely to become infected but also how contagion unfolds over time. Similar agreement is observed for noncollaboration networks that are not derived from bipartite projections (figs. S11 and S12), as well as across different coarse-graining scales (fig. S13). Together, these results show that ISCG preserves the spatiotemporal organization of contagion across diverse transmission regimes, network classes, and coarse-graining scales.

**Fig. 4.**
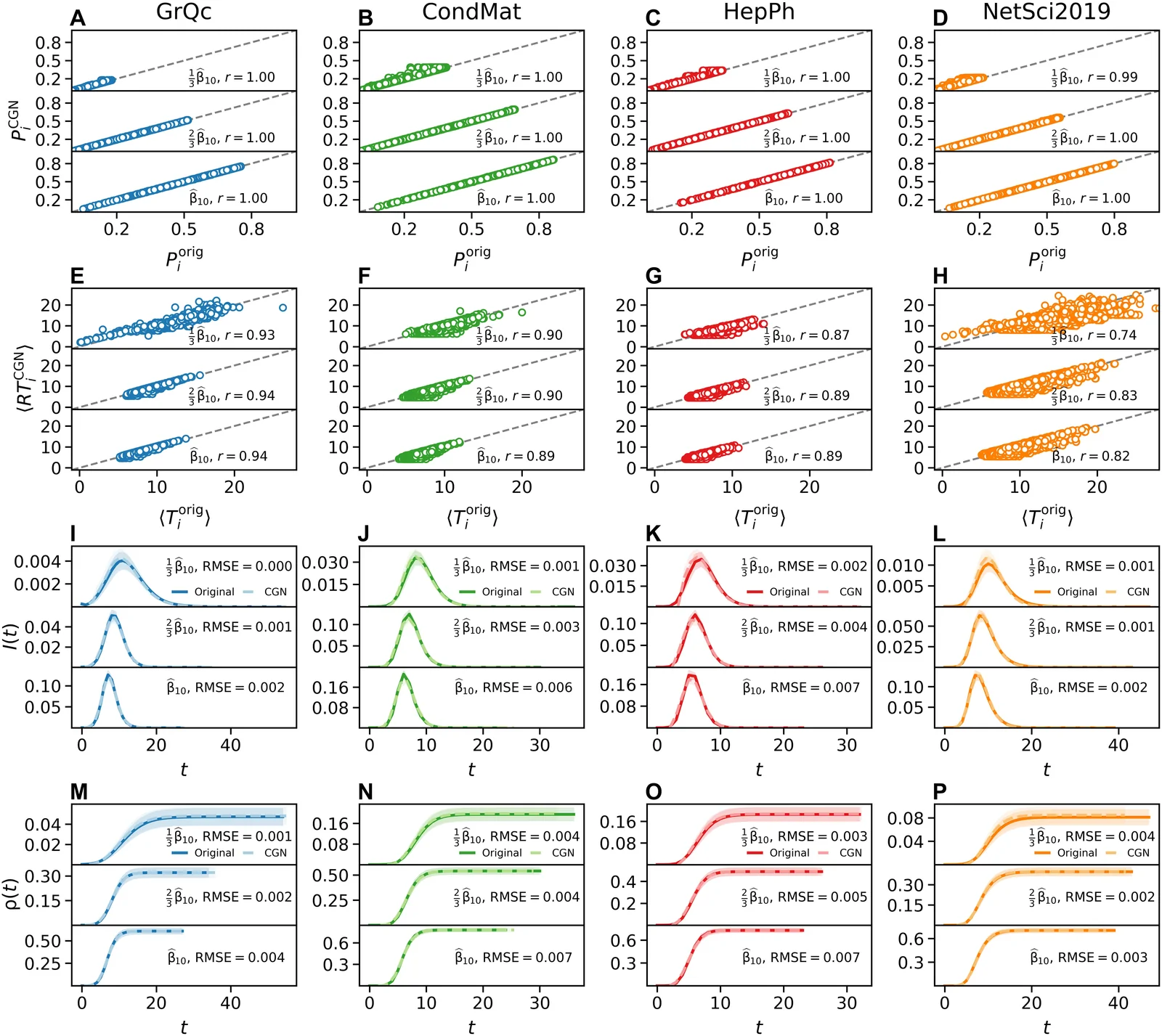
Preservation of spatiotemporal contagion dynamics across transmission regimes. Results compare the original networks with their corresponding k=10 clique CGNs for four representative collaboration networks: (**A**, **E**, **I**, and **M**) GrQc, (**B**, **F**, **J**, and **N**) CondMat, (**C**, **G**, **K**, and **O**) HepPh, and (**D**, **H**, **L**, and **P**) NetSci2019. For each network, results are shown for three transmission regimes: $\frac{{\hat{\beta }}_{10}}{3}=0.193$, $\frac{2{\hat{\beta }}_{10}}{3}=0.387$, and ${\hat{\beta }}_{10}=0.58$. Rows from top to bottom compare node-level infection probabilities $\left(P_{i}^{\text{orig}}\;\text{versus}\;P_{i}^{\text{CGN}}\right)$, mean infection times $\left(\langle T_{i}^{\text{orig}}\rangle \;\text{versus}\;\langle T_{i}^{\text{CGN}}\rangle +\Delta T\right)$, transient infected fractions *I*(*t*), and cumulative outbreak trajectories ρ(t). Mean infection times and temporal trajectories are shown after applying the global temporal shift t→t+ΔT, where $\Delta T=\langle T^{\text{orig}}\rangle -\langle T^{\text{CGN}}\rangle$. Pearson correlation coefficients *r* are reported for the node-level comparisons, and RMSE values are reported for the temporal trajectories. The RMSE values are computed pointwise between the original curves and the temporally shifted CGN curves over the full time window shown. Panels (A) to (P) were computed from 100 randomly selected outbreak seeds, each averaged over 500 independent epidemic realizations; shaded regions in panels (I) to (P) denote the SE across outbreak seeds. Across all transmission regimes, including those well below the theoretically predicted matching rate ${\hat{\beta }}_{10}$, the CGNs closely reproduce node-level infection probabilities, temporal ordering of infection events, transient prevalence dynamics, and cumulative outbreak trajectories. These results show that high spatiotemporal fidelity can be maintained well beyond the exact-preservation regime.

#### Preservation of final epidemic outcomes

We next characterize the preservation of final epidemic outcomes across transmission rates and coarse-graining scales. Figure 5A shows that the final outbreak size of the CGNs closely follows that of the original network over the full range of transmission rates β. Exact agreement is achieved once the transmission rate reaches the theoretically predicted matching condition $\beta ={\hat{\beta }}_{k}$ (inset). The same exact-agreement regime is reflected in the node-level infection-risk error (Fig. 5B), showing that preservation extends beyond the overall outbreak size to the heterogeneous infection risks of individual nodes. For each *k*-clique CGN, the microscopic discrepancy rapidly decreases to zero as β approaches the corresponding matching rate ${\hat{\beta }}_{k}$. The representative cases, 6, and 10 illustrate how the exact-agreement regime shifts with coarse-graining scale. Below the matching condition, the error increases gradually rather than abruptly, indicating a smooth crossover from exact to approximate agreement. We further assess how coarse-graining affects the epidemic threshold. Figure 5C shows that the susceptibility peaks of the original network and CGN remain closely aligned, although small shifts are observed for $\beta <{\hat{\beta }}_{k}$, where exact agreement is not expected. Despite these shifts, the CGNs provide a close approximation to the epidemic threshold. Similar qualitative behavior is observed for the remaining collaboration networks (CondMat, HepPh, and NetSci2019; fig. S14), indicating that these preservation regimes are not specific to the GrQc network.

**Fig. 5.**
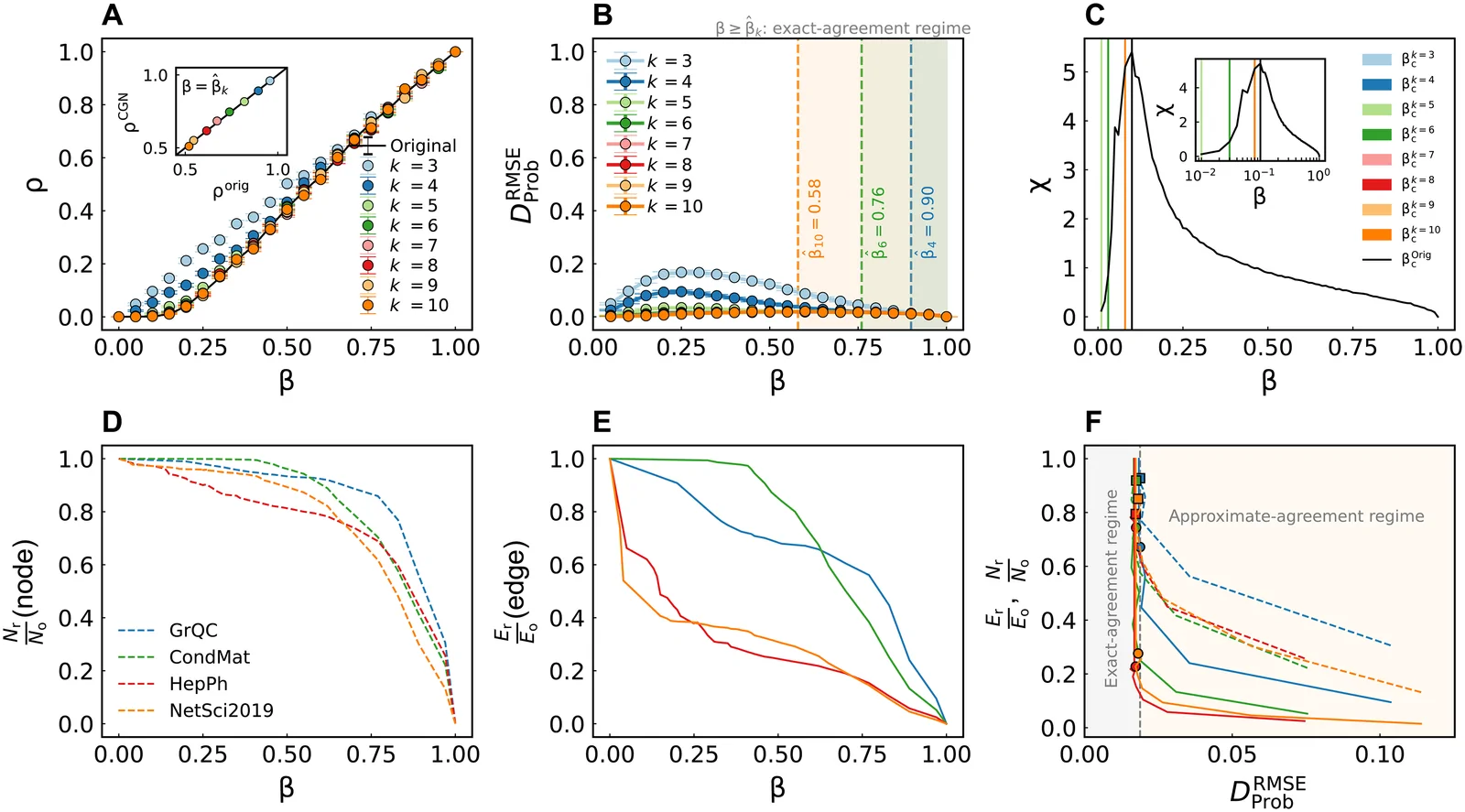
Preservation of final epidemic outcomes across coarse-graining scales. Results in (**A**) to (**C**) are shown for the GrQc collaboration network. (A) Final outbreak fraction as a function of transmission probability β for the original network (solid line) and *k*-clique CGNs (k=3 to 10; circles). Error bars show SEs over 1000 independent epidemic realizations with random initial seeds. Inset: Comparison between $\rho^{\text{orig}}$ and $\rho^{\text{CGN}}$ at $\beta ={\hat{\beta }}_{k}$, with ${\hat{\beta }}_{k}=\left(0.96,0.90,0.83,0.76,0.71,0.66,0.61,0.58\right)$ for k=3,…,10. (B) RMSE $D_{\text{prob}}^{\text{RMSE}}\left(\beta \right)=\sqrt{\frac{1}{N}\sum_{i=1}^{N}{\left[P_{i}^{\text{orig}}\left(\beta \right)-P_{i}^{\text{CGN}}\left(\beta \right)\right]}^{2}}$ between node-level infection probability of the original network and CGN as a function of β. The shaded region indicates the regime $\beta \geq {\hat{\beta }}_{k}$, in which the *k*-clique CGNs achieve exact agreement with the original network. (C) Susceptibility $\chi =\left[\langle s^{2}\rangle -{\langle s\rangle }^{2}\right]/\langle s\rangle$ as a function of β (*62*). The peaks identify the epidemic threshold $\beta_{c}$ of the original network (black) and the corresponding thresholds of the CGNs (colored lines). Inset: Logarithmic view highlighting the vicinity of the epidemic thresholds. (**D**) Fraction of retained nodes in the smallest *k*-clique CGN that satisfies the exact-agreement criterion at each transmission rate β (dashed). (**E**) Corresponding fraction of retained edges (solid). (**F**) Trade-off between compression and microscopic accuracy at $\beta ={\hat{\beta }}_{10}=0.58$. Dashed and solid curves denote the retained node (dashed) and edge (solid) fractions, respectively, as a function of $D_{\text{prob}}^{\text{RMSE}}$. The shaded regions indicate the exact-agreement and approximate-agreement regimes. Benchmark points (k=10; squares and circles) mark the exact reduction at $\beta ={\hat{\beta }}_{10}=0.58$. Significant compression is achievable beyond exact preservation with only minor loss of accuracy. These results show that ISCG preserves the final epidemic outcomes over a broad range of transmission probabilities while enabling substantial structural compression. Points in (B) and (F) are averaged over 100 initial seeds, with 1000 epidemic realizations per seed.

#### Compression under exact preservation

The theoretically predicted matching condition provides a direct criterion for determining how much of the network can be removed while still reproducing the epidemic outcomes exactly. Figure 5 (D and E) quantifies this limit by reporting the node and edge retention ratios for the smallest clique size satisfying the matching condition at each transmission rate. As the transmission rate increases, progressively smaller cliques satisfy the matching condition, allowing a larger fraction of the network to be contracted while remaining within the exact-preservation regime. Consequently, both node ($N_{r}/N_{o}$) and edge ($E_{r}/E_{o}$) retention ratios decrease monotonically with increasing transmission rate. This behavior arises because stronger transmission enables infection to saturate within progressively smaller cliques before recovery, whereas weaker transmission requires larger cliques to remain unresolved to preserve epidemic outcomes exactly. Despite this conservative exactness requirement, substantial structural reduction is still achieved. For example, in the NetSci2019 and HepPh networks, more than half of the original edges can already be removed at relatively low β while remaining within the exact-agreement regime, indicating that a substantial fraction of network connectivity is redundant for reproducing the epidemic outcomes.

#### Compression beyond exact preservation

Exact preservation defines a conservative operating point of the ISCG framework. Relaxing this requirement reveals a substantially broader compression-accuracy landscape, in which additional structural reduction can be achieved while maintaining high fidelity to the epidemic outcomes. Figure 5F quantifies this trade-off at a fixed transmission rate, $\beta ={\hat{\beta }}_{10}=0.58$. Starting from the original network, progressively decreasing the clique size *k* produces increasingly stronger structural compression. The k=10 clique CGN (marked by the square and circle symbols) represents the smallest CGN that still satisfies the exact-preservation condition. Further contraction below this scale leads to a smooth increase in node-level infection-risk error rather than an abrupt loss of agreement, indicating a controlled departure from exact preservation. This gradual crossover demonstrates that additional compression is possible beyond the exact-preservation regime while introducing only modest deviations in epidemic outcomes.

The same behavior is consistently observed across all 21 empirical networks analyzed in Fig. 6 (A and B). Despite substantial differences in network size and topology, all networks exhibit a common compression-accuracy trajectory. This trajectory consists of an initial high-fidelity plateau, in which substantial node and edge reduction is achieved with negligible microscopic error, followed by a gradual crossover where further compression incurs progressively larger deviations. Colored curves highlight representative high- and low-compressibility networks, whereas gray curves show the remaining datasets. Notably, edge reduction consistently exceeds node reduction, indicating that ISCG preferentially removes structurally redundant connectivity while preserving the dominant pathways governing contagion.

**Fig. 6.**
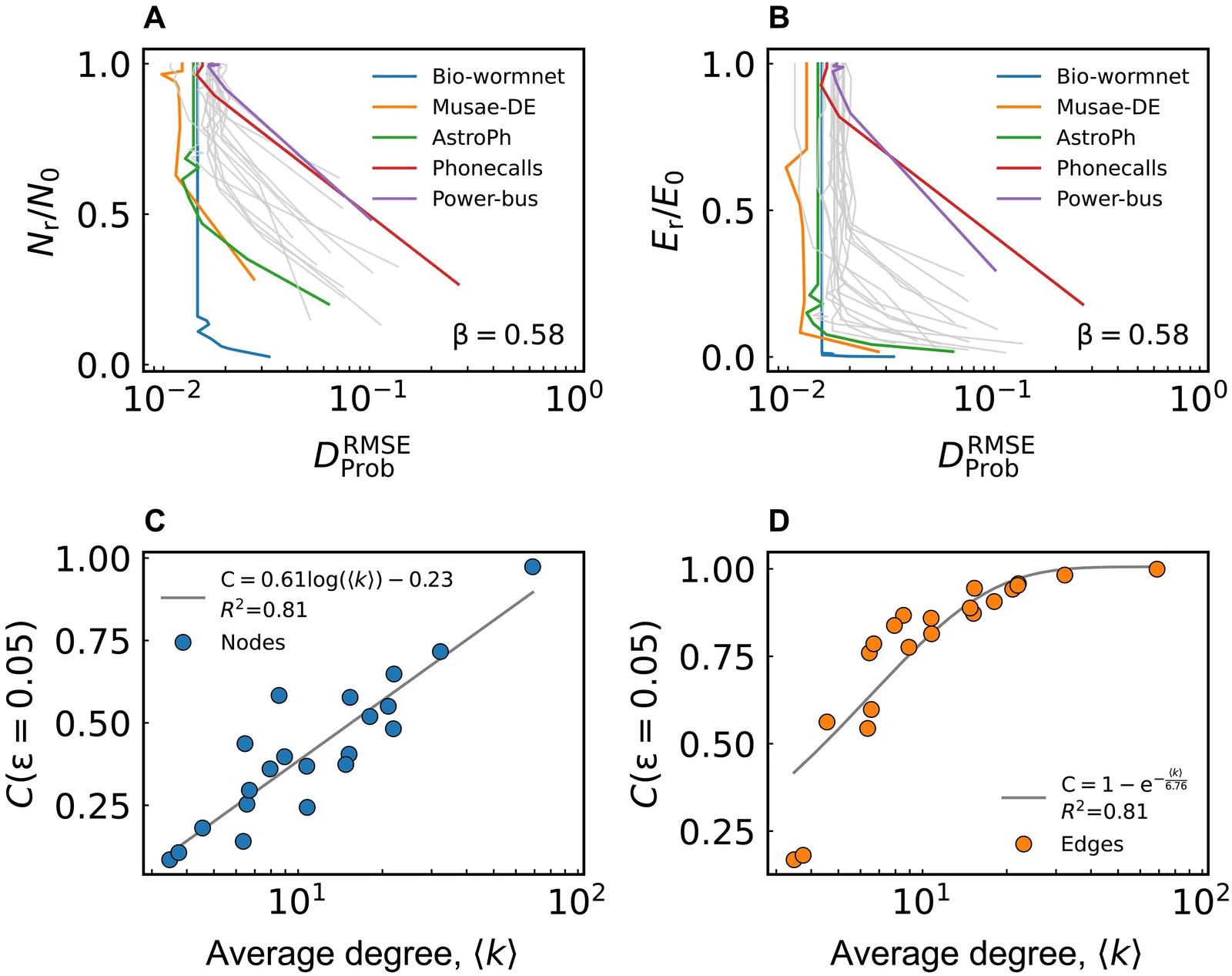
Accuracy-compression trade-off and its structural determinants. (**A** and **B**) Accuracy-compression trade-off across 20 empirical networks at fixed transmission probability β=0.58. For each network, varying the coarse-graining scale *k* generates a CGN trajectory relating the retained node fraction $N_{r}/N_{0}$ (A) and retained edge fraction $E_{r}/E_{0}$ (B) to the node-level infection-risk RMSE $D_{\text{prob}}^{\text{RMSE}}\left(\beta \right)=\sqrt{\frac{1}{N}\sum_{i=1}^{N}{\left[P_{i}^{\text{orig}}\left(\beta \right)-P_{i}^{\text{CGN}}\left(\beta \right)\right]}^{2}}$. Points in (A) and (B) are averaged over 100 initial seeds, with 1000 epidemic realizations per seed. Colored curves highlight representative high- and low-compressibility networks, whereas gray curves show the remaining datasets. Across networks, the curves exhibit an initial high-fidelity plateau, followed by a smooth crossover in which further compression incurs increasing microscopic error. Edge reduction consistently exceeds node reduction at comparable microscopic accuracy. (**C** and **D**) Structural determinants of compressibility. For each network, the compressibility score C(ε=0.05) is defined as the maximum reduction attainable at β=0.58 while maintaining $D_{\text{prob}}^{\text{RMSE}}\leq \varepsilon$. Specifically, $C=1-\frac{N_{r}}{N_{o}}$ for node compression (C) and $C=1-\frac{E_{r}}{E_{o}}$ for edge compression (D). Compressibility increases with average degree 〈k〉: Node-level compressibility grows approximately logarithmically, whereas edge-level compressibility rapidly saturates. The fitted trends are shown with $R^{2}=0.81$.

The achievable compression is also strongly influenced by the average degree of the original network. Figure 6 (C and D) shows that, at a fixed accuracy tolerance, denser networks admit substantially stronger compression. Using the compressibility score C(ε), defined as the maximum structural reduction attainable under a prescribed microscopic error tolerance, we find that node-level compressibility increases approximately logarithmically with average degree, whereas edge-level compressibility rapidly approaches saturation. These trends reflect the increasing abundance of redundant intraclique connectivity in denser networks. The compression-accuracy relationship is robust across transmission regimes (fig. S15). Similar trade-offs are observed at both lower (β=0.36) and higher (β=0.83) transmission rates, with stronger transmission permitting greater compression at a fixed accuracy because infection probabilities become progressively less sensitive to fine-grained structural details. These results show that the exact-preservation regime provides a conservative baseline, whereas controlled approximation enables substantially greater compression with predictable and quantifiable losses in accuracy.

Last, we find that the framework extends naturally to weighted contact networks: Edge-weight aggregation yields coarse-grained representations that closely reproduce both epidemic outcomes and spatiotemporal contagion dynamics despite substantial edge-weight heterogeneity (fig. S16).

### Application of the ISCG framework

The ISCG framework enables multiscale representations of large empirical networks while preserving the epidemic behavior relevant to the original system. To illustrate its practical utility, we consider two complementary applications: (i) multiscale epidemic modeling on a large-scale human mobility network and (ii) intervention strategies that exploit dynamically coherent structural units identified through coarse-graining.

#### Multiscale epidemic modeling in a large-scale mobility network

Large human mobility networks are among the most challenging systems for epidemic modeling because they combine massive numbers of contacts with strong mesoscale organization. To demonstrate the practical value of ISCG, we apply the framework to the YJMob100K dataset (*43*), which reconstructs an urban contact network from high-resolution mobility trajectories. Individuals colocated within spatial cells of 500 m×500 m during 30-min intervals are connected, naturally forming densely connected local interaction groups. Aggregating these interactions over 1 day yields a contact network with 95,131 nodes and 4,633,574 edges, providing a demanding test for multiscale epidemic modeling.

Figure 7 shows that ISCG naturally generates a hierarchy of CGNs rather than a single reduced representation. As illustrated in Fig. 7 (A and B), the original mobility network is progressively reduced from an 11-clique CGN to a much smaller 10-clique CGN. Despite this substantial reduction, SIR simulations initiated from the same seed produce nearly identical epidemic outcomes, with 37,077 and 37,048 infected nodes, respectively. The hierarchy extends continuously across clique scales. Figure 7C shows that decreasing the clique threshold yields progressively more compact network representations, allowing different structural resolutions to be selected according to the desired balance between computational efficiency and epidemic fidelity. This increase in compression does not compromise epidemic outcomes. Figure 7D shows that the CGNs remain in close agreement with the original network over a broad range of transmission probabilities despite the substantial reduction in network size. These results show that ISCG provides a family of multiscale representations for large empirical contact networks while preserving aggregate epidemic outcomes. This hierarchy enables epidemic processes to be studied at resolutions matched to the application, providing a practical foundation for scalable epidemic modeling in large networked systems.

**Fig. 7.**
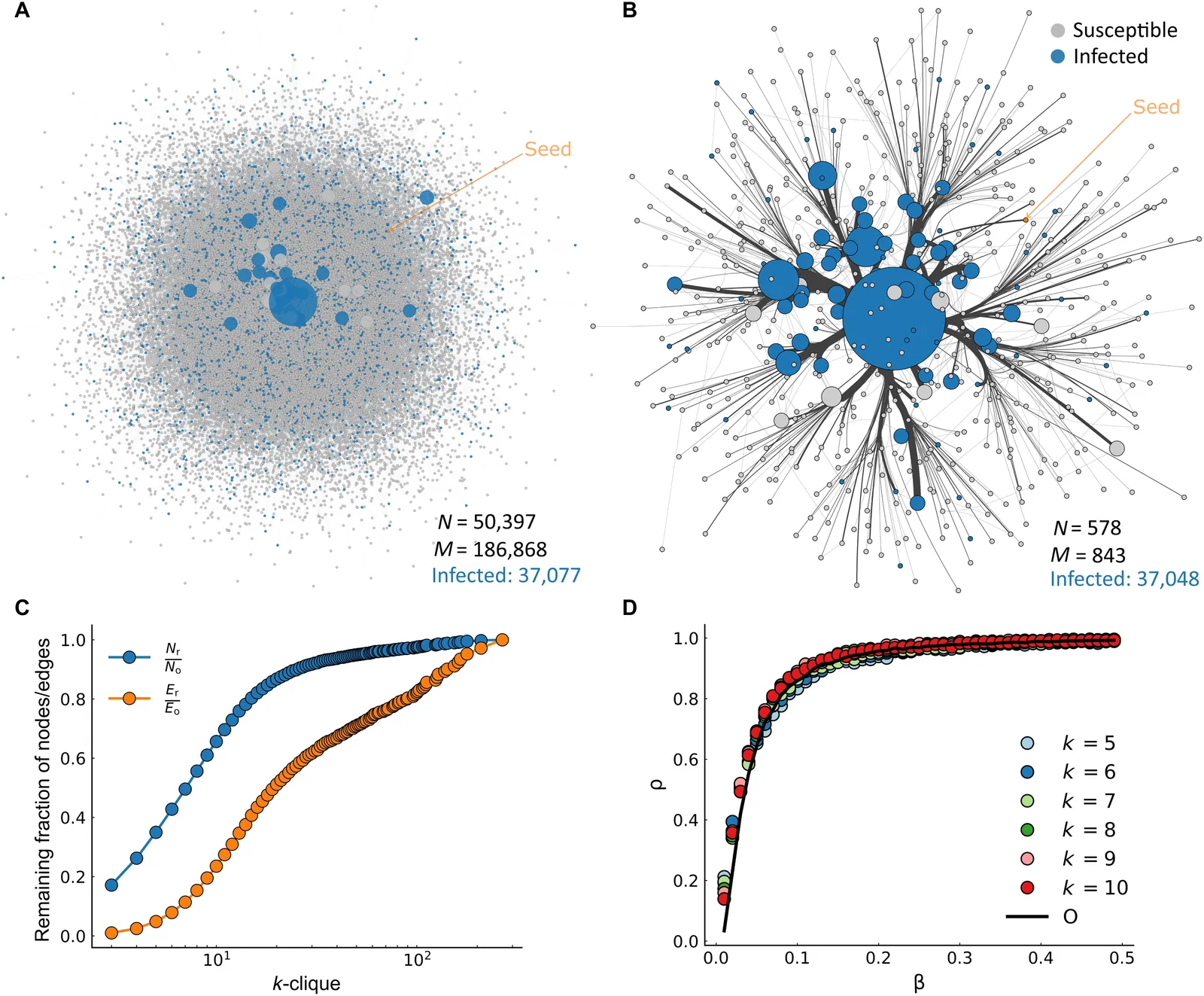
Application of ISCG to a large-scale human mobility network. (**A** and **B**) Visualization of progressive coarse-graining of a city-scale mobility network. An 11-CGN contains N=50,397 nodes and M=186,868 edges (A), whereas further reduction to a 10-clique CGN yields a compact representation with N=578 nodes and M=843 edges (B). Node size indicates the supernode weight, defined as the number of original nodes aggregated into each coarse-grained node. SIR simulations initiated from the same seed produce nearly identical outbreak sizes in the two CGNs, with 37,077 and 37,048 infected nodes, respectively. (**C**) Retained node and edge counts as functions of clique scale *k*. The original network contains N=95,131 nodes and M=4,633,574 edges. (**D**) Final infection density as a function of transmission probability β for the original network (solid line) and *k*-clique CGNs (k=5 to 10; markers). The close agreement across coarse-graining scales shows that ISCG preserves epidemic outcomes while producing substantial structural compression in a large mobility network.

#### Contagion control strategies guided by coarse-grained structures

Beyond reducing network complexity, ISCG filters out structurally redundant or dynamically negligible connections, yielding multiscale representations that emphasize the pathways and units most relevant for contagion spread. These representations not only facilitate efficient simulation but also expose mesoscale organizational structures that govern transmission dynamics, providing a principled basis for targeted intervention. To illustrate this idea, we consider three canonical contagion-control problems (*44*): IM, edge-based immunization (EI), and SS. For each task, we develop an ISCG-based strategy that exploits multiscale network structure while retaining node-level resolution when required. Across multiple empirical networks and transmission regimes, these approaches consistently outperform widely used centrality-based heuristics.

##### Influence maximization

IM seeks a seed set that maximizes the expected outbreak size, typically measured by the final infection density ρ (*40*, *41*, *45*, *46*). ISCG-IM addresses this problem by first identifying influential supernodes in the CGN and then selecting high-degree nodes within each supernode as seeds in the original network (Fig. 8A). This two-level strategy promotes broad structural coverage while avoiding redundant seed placement (fig. S17, A and B). As a result, ISCG-IM consistently outperforms adaptive centrality–based methods, including degree-based, *k*-core (*40*), betweenness, closeness, eigenvector (*47*), Katz (*48*), subgraph (*49*), collective influence (*46*), and nonbacktracking centrality (*50*). As shown in Fig. 8B, ISCG-IM achieves larger outbreak sizes for a given number of seeds at $\beta =2\beta_{c}$, a trend that persists across transmission probabilities (Fig. 8C) and network topologies (Fig. 8D). The improvement arises because the coarse-grained representation guides seed placement across distinct mesoscale regions, reducing redundancy while maintaining strong local spreading capability (fig. S17).

**Fig. 8.**
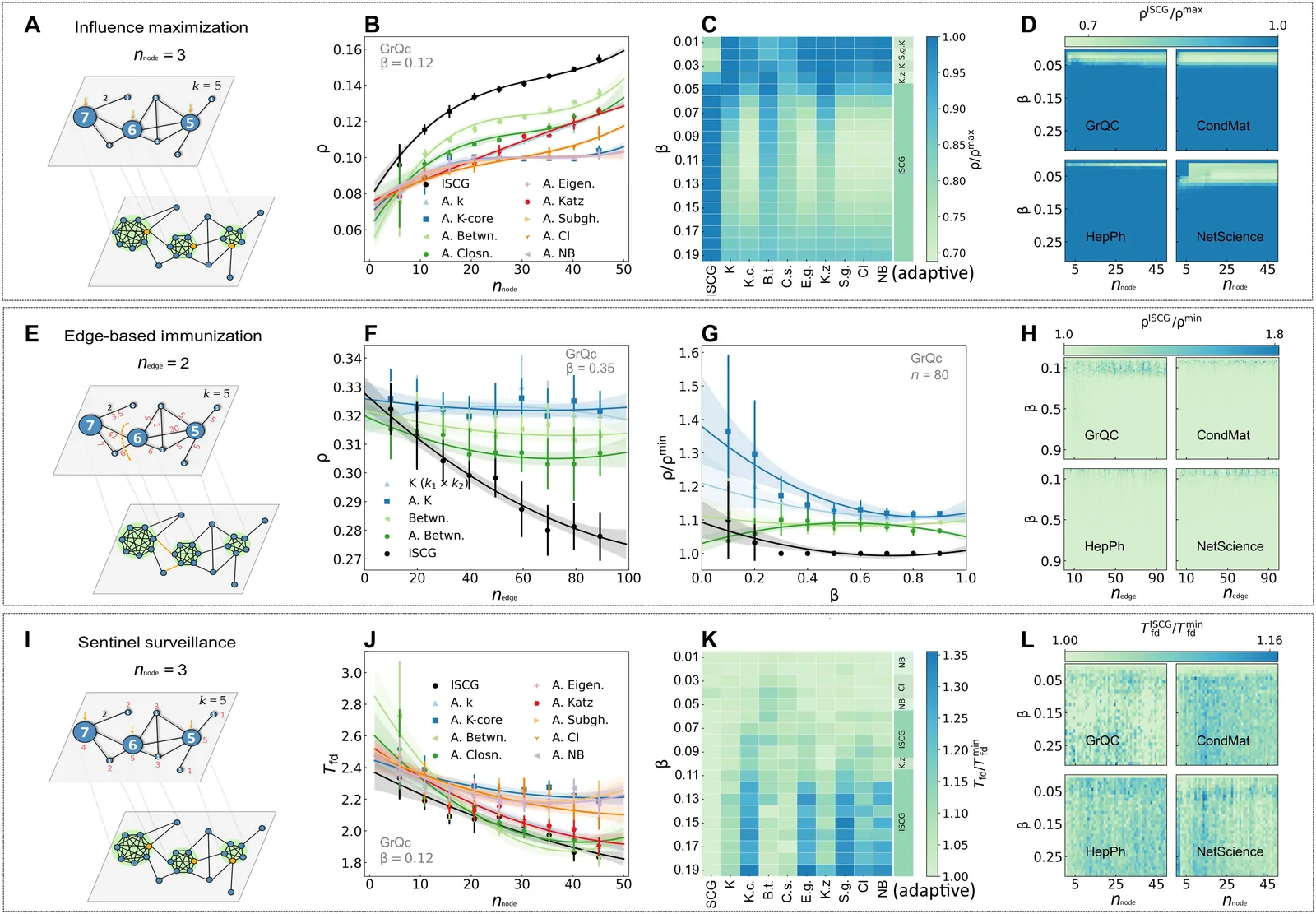
Application of the ISCG framework to canonical contagion control tasks. The ISCG framework uses *k*-clique CGNs to simplify network structure while preserving contagion dynamics, enabling scalable solutions to three representative tasks: IM, EI, and SS. (**A** to **D**) ISCG-IM. (A) Supernodes in a 5-clique CGN are ranked to select candidate regions (orange arrows), from which seed nodes (orange nodes) are chosen in the original network. (B) Final outbreak size ρ as a function of the number of selected nodes $n_{\text{node}}$ at $\beta =2\beta_{c}$. (C) Maximal outbreak size $\rho^{\text{max}}$ versus transmission rate β ($n_{\text{node}}=30$). (D) Heatmap summarizing ISCG-IM performance across four networks as a function of β and $n_{\text{node}}$. (**E** to **H**) ISCG-EI. (E) Superedges are scored by the product of endpoint supernode weights normalized by the superedge weight (red labels). Top-ranked superedges are selected, mapped to minimum edge cuts (orange dashed lines), and removed in the original network (orange edges). (F) Infection density ρ as a function of the number of removed edges $n_{\text{edge}}$ at $\beta =5\beta_{c}$. (G) Minimal outbreak size $\rho^{\text{min}}$ versus β ($n_{\text{edge}}=80$). (H) Heatmap of ISCG-EI performance across four networks. (**I** to **L**) ISCG-SS. (I) Supernodes are ranked to identify regions with high exposure (orange arrow), from which sentinel nodes (orange nodes) are selected. (J) Earliest detection time $T_{\text{fd}}^{\text{min}}$ as a function of $n_{\text{node}}$ at $\beta =2\beta_{c}$. (K) Comparison of $T_{\text{fd}}^{\text{min}}$ across β ($n_{\text{node}}=30$). (L) Heatmap of ISCG-SS performance across four networks. Bars in (C) and (K) indicate the best-performing method at each β; results in these panels are shown for the GrQc network.

##### Edge-based immunization

EI suppresses contagion by removing transmission links (*38*, *39*). ISCG-EI ranks interactions between supernodes in the CGN and maps the selected superedges back to the corresponding edge sets in the original network (Fig. 8E). Operating on the multiscale representation allows the method to target interactions between dynamically coherent groups rather than isolated edges. As shown in Fig. 8F, ISCG-EI suppresses outbreaks more effectively than baseline methods as the number of removed edges increases. This advantage persists across transmission probabilities (Fig. 8G) and network structures (Fig. 8H) because targeting superedges concentrates the intervention on intergroup connections with disproportionate influence on aggregate spreading (fig. S18).

##### Sentinel surveillance

SS aims to detect outbreaks as early as possible (*51*, *52*). ISCG-SS first identifies highly exposed regions in the CGN and then selects representative nodes within those regions as sentinels in the original network (Fig. 8I). This strategy combines mesoscale exposure with local node-level resolution. Consistent with this design, ISCG-SS achieves earlier outbreak detection than existing approaches at $\beta =2\beta_{c}$ (Fig. 8J). This improvement persists across transmission probabilities (Fig. 8K) and empirical networks (Fig. 8L) because the coarse-grained representation concentrates sentinel placement in exposed mesoscale regions while retaining local node-level resolution (fig. S19).

## DISCUSSION

The central insight of this work is that dense local structures define the natural dynamical units of contagion. Once infection saturates within a clique, epidemic spreading beyond the clique is governed primarily by transmission between cliques rather than by the detailed dynamics of individual nodes. Contagion therefore evolves naturally across scales, with rapid homogenization within dense local groups followed by propagation between them. This multiscale organization provides the basis for the ISCG framework. By representing dynamically coherent groups as supernodes and the interactions between them as superedges, ISCG constructs a hierarchy of CGNs that preserves the essential organization of contagion. Above the theoretically predicted matching condition, $\beta \geq {\hat{\beta }}_{k}$, these coarse-grained representations reproduce not only aggregate epidemic outcomes but also the spatiotemporal organization of contagion. After a single global temporal alignment, they further preserve infection timing and the complete spreading trajectory. Beyond the regime of exact preservation, the same framework naturally extends to controlled approximations. Relaxing the clique-saturation condition allows stronger structural compression while keeping the loss of dynamical fidelity small and quantifiable. This flexibility allows the coarse-graining level to be tailored to the desired balance between dynamical fidelity, structural simplicity, and computational cost.

The significance of ISCG lies beyond computational acceleration, although substantial reductions in simulation cost are often achieved. Many epidemic models already admit highly efficient simulation algorithms, including percolation-based and event-driven approaches capable of generating realizations at near-linear or sublinear cost (*14*, *53*, *54*). The distinctive contribution of ISCG lies instead in providing an interpretable multiscale representation of contagion. Unlike reduced-order models that replace the original network with aggregate equations or scalar descriptors, ISCG preserves an interpretable network organized around contagion dynamics. Supernodes correspond to effective spreading units, and superedges describe the aggregate transmission pathways between them. By exposing these effective spreading units and their aggregate interactions, the coarse-grained representation can support simulation, mechanistic interpretation, visualization, and intervention design within the same structural description.

Although the present theory focuses on unweighted static networks, the framework extends naturally to more general settings. Extending the analytical theory to weighted, directed, or temporal networks is more challenging because intraclique heterogeneity breaks the symmetry underlying the present exactness conditions. Similarly, the current theoretical guarantees are derived for discrete-time contagion with deterministic recovery. Generalizing the theory to stochastic recovery, temporal networks, non-Markovian dynamics, and higher-order contagion processes (*55*–*58*) therefore represents a natural direction for future work.

The multiscale perspective developed here are not restricted to epidemic spreading. Any dynamical process in which interactions become effectively homogenized within dense local structures, including information diffusion (*2*), synchronization (*59*), cascading failures (*60*), and evolutionary dynamics (*61*), may admit similar multiscale descriptions. More generally, these results suggest that coarse-graining should be viewed not merely as a computational strategy but also as a means of identifying the dynamically relevant organization of complex systems. From this perspective, ISCG provides a general framework for uncovering the mesoscale organization that links microscopic interactions to macroscopic dynamics.

## METHODS

### Numerical simulations

All numerical results are obtained from ensemble simulations of the contagion dynamics. Unless otherwise stated, each reported quantity is averaged over 1000 independent epidemic realizations initiated from uniformly selected random seed nodes. To compare the original network and CGN, we evaluate observables characterizing both final epidemic outcomes and spatiotemporal contagion dynamics. Final-outcome preservation is assessed using outbreak size and node-level infection probabilities, whereas dynamical fidelity is evaluated using node-level infection times together with the temporal evolution of the infected fraction and cumulative outbreak trajectory. Outbreak size is computed directly from the simulation ensemble, whereas the remaining observables are defined below.

#### Lifting coarse-grained observables

Because coarse-graining changes the node-level state space of the system, observables computed on the CGN are first mapped back to the original node space before comparison. Specifically, each original node is assigned the observable associated with its corresponding supernode. This lifting procedure enables direct comparison of node-level and temporal observables between the original network and CGN within a common node-level state space.

#### Node-level infection probability

For each node *i*, the infection probability $P_{i}$ is defined as the fraction of realizations in which node *i* becomes infected before epidemic termination. After lifting, the node-level infection-risk error is quantified by the RMSE between the infection-probability vectors of the original network and CGN.

#### Mean infection time

For each node *i*, the mean infection time $\langle T_{i}\rangle$ is computed by averaging the infection time of node *i* over all realizations in which the node becomes infected. The lifted mean infection times are used to compare infection ordering between the original network and CGN.

#### Spatiotemporal propagation trajectories

The transient infected fraction *I*(*t*) and cumulative outbreak trajectory ρ(t) are averaged over the simulation ensemble. Before comparison, the coarse-grained trajectories are aligned by a global temporal shift

$$\Delta T=\langle T^{\text{orig}}\rangle -\langle T^{\text{CGN}}\rangle$$

where $\langle T^{\text{orig}}\rangle$ and $\langle T^{\text{CGN}}\rangle$ denote the average infection times over all infected nodes in the original network and CGN, respectively. After temporal alignment, spatiotemporal agreement is quantified by the pointwise RMSE between the original and coarse-grained trajectories over the full simulation window.

### Consistency of contagion dynamics

A central requirement of the ISCG framework is that contagion dynamics remain well defined and comparable under structural coarse-graining. Because coarse-graining reduces the dimensionality of the network, ensuring that dynamical equivalence requires an explicit correspondence between states in the CGN and configurations in the original system. To formalize this correspondence, we introduce an inverse coarse-graining operator that lifts coarse-grained contagion states back to the original network.

This construction provides a precise theoretical foundation for assessing when contagion dynamics are preserved. From it, we derive two conditions for dynamical equivalence: (i) preservation of the final outbreak size and (ii) preservation of configuration probabilities. As proven in Propositions 1 and 2, these conditions jointly guarantee macroscopic and microscopic consistency between the original network and its coarse-grained representation under analytically defined constraints. They therefore establish a principled basis for both exact and controlled approximate reductions.

#### Inverse coarse-grained operator

The inverse operator H{⋅} reconstructs contagion states in the original network from configurations defined on a *k*-clique CGN. It acts both at the level of individual supernodes and at the level of full contagion configurations, thereby enabling direct comparison of dynamical observables across scales.

1) Supernode reconstruction. For a supernode $v_{i}\left(k\right)$ in the *k*-clique CGN $G_{R=k}$, the operator $H\left\{v_{i}\left(k\right)\right\}$ returns the induced subgraph in the original network that was iteratively aggregated into $v_{i}\left(k\right)$. This reconstruction follows the nested hierarchy of clique contractions

$$v_{i}\left(k\right)\overset{G_{\Delta_{k}}}{⟶}\cdots \overset{G_{\Delta_{n}}}{⟶}\cdots \overset{G_{\Delta_{K}}}{⟶}H\left\{v_{i}\left(k\right)\right\}$$ (8)

where $G_{\Delta_{k}}$ denotes the CGN at clique scale *k*, and $H\left\{v_{i}\left(k\right)\right\}$ is the corresponding subgraph in the original network.

2) Contagion configuration lifting. Given a contagion configuration M defined on $G_{R=k}$, the operator H{M} maps it to a configuration in the original network by assigning all nodes aggregated into an infected supernode to the infected state

$$H\left\{M\right\}=\left\{H\left\{v_{i}\left(k\right)\right\}|v_{i}\left(k\right)\in V\left(k\right),\;s\left(v_{i}\left(k\right)|G_{R=k}\right)=1\right\}$$ (9)

Here, $s\left(v_{i}\left(k\right)|G_{R=k}\right)=1$ indicates that the supernode $v_{i}\left(k\right)$ is infected. The size of each reconstructed subgraph equals the weight $v_{i}^{w}\left(k\right)$ of the supernode, corresponding to the number of original nodes it aggregates. This inverse-mapping formalism aligns the state spaces of the original and coarse-grained systems and enables consistent computation of dynamical observables, including final outbreak size and configuration probabilities. It thus provides the theoretical mechanism through which ISCG preserves contagion dynamics across scales.

#### Conditions for maintaining contagion dynamics

To ensure that a CGN faithfully replicates the contagion behavior of the original system, we derive two theoretical conditions that form the foundation of the ISCG framework. These conditions guarantee the preservation of both the expected final outbreak size and the configuration probability distribution, and are rigorously established in Propositions 1 and 2.

We begin by expressing the expected outbreak size for a contagion process initiated at node $v_{i}$ in the original network *G*

$$\langle \rho_{i}\left(G\right)\rangle =\sum_{M^{\prime }\in \Omega_{i}^{G}}P\left(M^{\prime }|G\right)\cdot s\left(M^{\prime }|G\right)$$ (10)

where $\Omega_{i}^{G}$ denotes the set of all possible contagion configurations initiated at $v_{i}$, $P\left(M^{\prime }|G\right)$ is the probability of configuration $M^{\prime }$, and $s\left(M^{\prime }|G\right)$ is the number of infected nodes in that configuration. This expression serves as a baseline for comparing contagion dynamics between the original network *G* and its coarse-grained counterpart $G_{R=k}$.

When the transmission probability satisfies $\beta \geq {\hat{\beta }}_{k}$, infection saturates within each *k*-clique before recovery occurs (Proposition 1). In this regime, each contagion configuration in the *k*-clique CGN corresponds one-to-one with a configuration in the original network via the inverse coarse-graining operator

$$H\left\{\Omega_{i}^{G_{R=k}}\right\}=\Omega_{i}^{G}$$ (11)

Under this condition, the expected outbreak size in the original network can be rewritten as

$$\langle \rho_{i}\left(G\right)\rangle =\sum_{M^{\prime }\in H\left\{\Omega_{i}^{G_{R=k}}\right\}}P\left(M^{\prime }|G\right)\cdot s\left(M^{\prime }|G\right)$$ (12)

whereas the corresponding expression in the CGN is

$$\langle \rho_{i}\left(G_{R=k}\right)\rangle =\sum_{M\in \Omega_{i}^{G_{R=k}}}P\left(M|G_{R=k}\right)\cdot s\left(M|G_{R=k}\right)$$ (13)

Dynamic equivalence holds if, for every configuration $M\in \Omega_{i}^{G_{R=k}}$, the following two conditions are satisfied

$$\left\{ \begin{array}{c} s\left(M|G_{R=k}\right)=s\left(M^{\prime }|G\right)\\ P\left(M|G_{R=k}\right)=P\left(M^{\prime }|G\right) \end{array}\right.$$ (14)

where $M^{\prime }=H\left\{M\right\}$. These conditions respectively ensure the preservation of outbreak size and configuration probabilities. When they are met—under the analytically derived thresholds in Eqs. 3 and 4—the expected outbreak sizes in the original network and CGN are identical

$$\langle \rho_{i}\left(G\right)\rangle =\langle \rho_{i}\left(G_{R=k}\right)\rangle$$ (15)

For $\beta <{\hat{\beta }}_{k}$, intraclique transmission may be incomplete, violating the one-to-one correspondence between configurations. In this subsaturation regime, partially infected clique states can arise in *G* that have no representation in $G_{R=k}$. This mismatch introduces controlled deviations in predicted dynamics rather than a breakdown of the framework. We provide an illustrative example in the section S2 to highlight the origin of these deviations. These results delineate the limits of exact equivalence and clarify the fidelity-compression trade-offs inherent to the ISCG coarse-graining procedure.

#### Validation of contagion dynamics consistency

The theoretical conditions established in Eqs. 3 and 4 guarantee the equivalence of two key dynamical quantities: (i) the final outbreak size and (ii) the probability distribution over contagion configurations. To validate these guarantees, we explicitly verify each component of Eq. 14. We begin by examining the equivalence of outbreak size between the original network and its *k*-clique coarse-grained representation.

##### Equivalence of final outbreak size

For a contagion configuration M on the *k*-clique CGN $G_{R=k}$, the total number of infected nodes is given by

$$s\left(M|G_{R=k}\right)=\sum_{v_{i}\left(k\right)\in V\left(k\right)}s\left(v_{i}\left(k\right)|G_{R=k}\right)\cdot v_{i}^{w}\left(k\right)$$ (16)

where *V*(*k*) denotes the node set of the CGN, $s\left(v_{i}\left(k\right)|G_{R=k}\right)\in \left\{0,1\right\}$ indicates whether supernode $v_{i}\left(k\right)$ is infected, and $v_{i}^{w}\left(k\right)$ is its weight, corresponding to the number of original nodes it aggregates.

The corresponding configuration in the original network is $M^{\prime }=H\left\{M\right\}$. The number of infected nodes in *G* under this configuration is

$$s\left(M^{\prime }|G\right)=s\left(H\left\{M\right\}|G\right)=\sum_{v_{i}\left(k\right)\in V\left(k\right)}s\left(H\left\{v_{i}\left(k\right)\right\}|G\right)$$ (17)

where $H\left\{v_{i}\left(k\right)\right\}$ denotes the subgraph in the original network that was aggregated into supernode $v_{i}\left(k\right)$, and $s\left(H\left\{v_{i}\left(k\right)\right\}|G\right)$ is the number of infected nodes within that subgraph.

When $\beta \geq {\hat{\beta }}_{k}$, Proposition 1 guarantees that infection saturates within each such subgraph before recovery occurs. As a result, all nodes aggregated into an infected supernode become infected, implying

$$s\left(H\left\{v_{i}\left(k\right)\right\}|G\right)=v_{i}^{w}\left(k\right)$$ (18)

Substituting this relation into Eq. 17 yields

$$s\left(M^{\prime }|G\right)=\sum_{v_{i}\left(k\right)\in V\left(k\right)}s\left(v_{i}\left(k\right)|G_{R=k}\right)\cdot v_{i}^{w}\left(k\right)$$ (19)

which is identical to Eq. 16. Therefore

$$s\left(M|G_{R=k}\right)=s\left(M^{\prime }|G\right)$$ (20)

establishing exact preservation of the final outbreak size under the condition $\beta \geq {\hat{\beta }}_{k}$. For $\beta <{\hat{\beta }}_{k}$, intraclique transmission may be incomplete. In this subsaturation regime, partially infected subgraphs can arise in the original network that have no representation in the CGN, leading to controlled discrepancies in outbreak size. An explicit example and a quantitative characterization of these deviations are provided in section S2.

##### Equivalence of contagion configuration probabilities

The second condition in Eq. 14 requires that each contagion configuration M in the CGN has the same probability as its corresponding configuration $M^{\prime }=H\left\{M\right\}$ in the original network. This ensures that coarse-graining preserves not only expected outbreak size but also the full stochastic structure of the contagion process.

For the *k*-clique CGN $G_{R=k}$, the probability of observing configuration M can be written as a sum over all transmission realizations that produce M

$$\begin{array}{cc} P\left(M|G_{R=k}\right)= & \sum_{P\in \Psi \left(M\right)}\prod_{\left(v_{i}\left(k\right)\to v_{j}\left(k\right)\right)\in P}P\left(v_{i}\left(k\right)\to v_{j}\left(k\right)|G_{R=k},\beta_{w_{\mathrm{ij}}\left(k\right)}\right)\\ & \times \prod_{\left(v_{m}\left(k\right)↛v_{n}\left(k\right)\right)\in P}\left[1-P\left(v_{m}\left(k\right)\to v_{n}\left(k\right)|G_{R=k},\beta_{w_{\mathrm{mn}}\left(k\right)}\right)\right] \end{array}$$ (21)

where Ψ(M) denotes the set of all realizations of successful and unsuccessful transmissions between supernodes that lead to configuration M.

The corresponding configuration in the original network is $M^{\prime }=H\left\{M\right\}$. Its probability in *G* is given by an analogous sum over transmission realizations between the reconstructed subgraphs

$$\begin{array}{cc} P\left(M^{\prime }|G\right)= & \sum_{P^{\prime }\in \Psi \left(H\left\{M\right\}\right)}\prod_{\left(H\left\{v_{i}\left(k\right)\right\}\to H\left\{v_{j}\left(k\right)\right\}\right)\in P^{\prime }}P\left(H\left\{v_{i}\left(k\right)\right\}\to H\left\{v_{j}\left(k\right)\right\}|G,\beta \right)\\ & \times \prod_{\left(H\left\{v_{m}\left(k\right)\right\}↛H\left\{v_{n}\left(k\right)\right\}\right)\in P^{\prime }}\left[1-P\left(H\left\{v_{m}\left(k\right)\right\}\to H\left\{v_{n}\left(k\right)\right\}|G,\beta \right)\right] \end{array}$$ (22)

where each realization $P^{\prime }$ represents a specific pattern of intersubgraph transmissions in the original network.

Proposition 2 establishes that, when $\beta \geq {\hat{\beta }}_{k}$ and the CGN transmission probabilities are defined as $\beta_{w_{\mathrm{ij}}\left(k\right)}=1-{\left(1-\beta \right)}^{e_{\mathrm{ij}}^{w}\left(k\right)}$, the effective transmission probability between two supernodes in the CGN exactly matches the probability of transmission between their corresponding subgraphs in the original network

$$\begin{array}{c} P\left(H\left\{v_{i}\left(k\right)\right\}\to H\left\{v_{j}\left(k\right)\right\}|G,\beta \right)\\ =P\left(v_{i}\left(k\right)\to v_{j}\left(k\right)|G_{R=k},\beta_{w_{\mathrm{ij}}\left(k\right)}\right) \end{array}$$ (23)

As a consequence, there exists a one-to-one correspondence between transmission realizations P∈Ψ(M) and $P^{\prime }\in \Psi \left(H\left\{M\right\}\right)$ with identical probabilities. Substituting Eq. 23 into Eqs. 21 and 22 therefore yields

$$P\left(M|G_{R=k}\right)=P\left(M^{\prime }|G\right)$$ (24)

This result establishes that ISCG preserves the full configuration probability distribution whenever the conditions in Eqs. 3 and 4 are satisfied. Combined with the exact preservation of outbreak size, this completes the proof of dynamic equivalence between the original and coarse-grained systems under clique-level saturation.

### Proposition 1

Let $v_{i}\left(k\right)\in V\left(k\right)$ be a supernode in the *k*-clique CGN $G_{R=k}$, representing the subgraph $H\left\{v_{i}\left(k\right)\right\}$ in the original network *G*. If the transmission probability satisfies $\beta \geq {\hat{\beta }}_{k}$ and the supernode is infected, i.e., $s\left(v_{i}\left(k\right)|G_{R=k}\right)=1$, then the entire subgraph is infected

$$s\left(H\left\{v_{i}\left(k\right)\right\}|G\right)=v_{i}^{w}\left(k\right)$$ (25)

where $v_{i}^{w}\left(k\right)$ denotes the number of original nodes aggregated into $v_{i}\left(k\right)$.

#### Proof

We distinguish two cases. Case 1: $v_{i}^{w}\left(k\right)=1$ (singleton). In this case, $H\left\{v_{i}\left(k\right)\right\}$ consists of a single node. Because $v_{i}\left(k\right)$ is infected in the CGN, the corresponding node in *G* is infected, and the statement holds trivially.

Case 2: $v_{i}^{w}\left(k\right)>1$ (multinode subgraph). The supernode $v_{i}\left(k\right)$ is formed by iteratively contracting a hierarchy of cliques with sizes n≥k via the operator F

$$H\left\{v_{i}\left(k\right)\right\}\overset{G_{\Delta_{K}}}{⟶}\cdots \overset{G_{\Delta_{n}}}{⟶}\cdots \overset{G_{\Delta_{k}}}{⟶}v_{i}\left(k\right)$$ (26)

where each $G_{\Delta_{n}}$ denotes an *n*-clique encountered during the iterative coarse-graining process, and K is the size of the largest clique. Reversing this construction, $H\left\{v_{i}\left(k\right)\right\}$ can be reconstructed as a collection Φ of overlapping cliques with sizes n∈[k,K].

The total number of infected nodes in $H\left\{v_{i}\left(k\right)\right\}$ can be written as

$$s\left(H\left\{v_{i}\left(k\right)\right\}|G\right)=\sum_{G_{\Delta }\in \Phi }s\left(G_{\Delta }|H\left\{v_{i}\left(k\right)\right\}\right)$$ (27)

By definition of the threshold ${\hat{\beta }}_{k}$, when $\beta \geq {\hat{\beta }}_{k}$ any *k*-clique becomes fully infected once at least one node is infected

$$P\left(1\to \left(k-1\right)|G_{\Delta_{k}},{\hat{\beta }}_{k}\right)=1$$ (28)

Moreover, because ${\hat{\beta }}_{k}\geq {\hat{\beta }}_{n}$ for all n>k (Fig. 2), the same full-infection property holds for all larger cliques

$$P\left(1\to \left(n-1\right)|G_{\Delta_{n}},{\hat{\beta }}_{k}\right)=1$$ (29)

Therefore, every clique $G_{\Delta_{n}}\in \Phi$ that contains at least one infected node becomes fully infected

$$s\left(G_{\Delta_{n}}|H\left\{v_{i}\left(k\right)\right\}\right)=n$$ (30)

Because $v_{i}\left(k\right)$ is infected in $G_{R=k}$, at least one node in $H\left\{v_{i}\left(k\right)\right\}$ is infected in *G*. Full intraclique transmission then implies that infection spreads across the entire overlapping clique structure, resulting in complete infection of $H\left\{v_{i}\left(k\right)\right\}$. Hence

$$s\left(H\left\{v_{i}\left(k\right)\right\}|G\right)=v_{i}^{w}\left(k\right)$$ (31)

This completes the proof.

### Proposition 2

Let $e_{\mathrm{ij}}\left(k\right)\in E\left(k\right)$ be a superedge in the *k*-clique CGN ($G_{R=k}$) connecting supernodes $v_{i}\left(k\right)$ and $v_{j}\left(k\right)$, which correspond to subgraphs $H\left\{v_{i}\left(k\right)\right\}$ and $H\left\{v_{j}\left(k\right)\right\}$ in the original network *G*. Denote by $e_{\mathrm{ij}}^{w}\left(k\right)$ the number of original edges connecting these two subgraphs. If the transmission probability satisfies $\beta \geq {\hat{\beta }}_{k}$, and the effective superedge transmission probability is defined as

$$\beta_{w_{\mathrm{ij}}\left(k\right)}=1-{\left(1-\beta \right)}^{e_{\mathrm{ij}}^{w}\left(k\right)}$$

then the contagion transmission probability between the two subgraphs in the original network is exactly reproduced by the corresponding superedge in the CGN

$$\begin{array}{c} P\left(H\left\{v_{i}\left(k\right)\right\}\to H\left\{v_{j}\left(k\right)\right\}|G,\beta \right)\\ =P\left(v_{i}\left(k\right)\to v_{j}\left(k\right)|G_{R=k},\beta_{w_{\mathrm{ij}}\left(k\right)}\right) \end{array}$$ (32)

#### Proof

Because $\beta \geq {\hat{\beta }}_{k}$, Proposition 1 guarantees that once a supernode is infected, all nodes within its corresponding subgraph are fully infected. Transmission between two supernodes therefore reduces to the probability that at least one of the $e_{\mathrm{ij}}^{w}\left(k\right)$ intersubgraph edges successfully transmits infection.

We consider two cases. Case 1: $e_{\mathrm{ij}}^{w}\left(k\right)=1$ (single-edge coupling). In this case, the two subgraphs are connected by a single edge in the original network. The probability of transmission between the subgraphs is simply $P\left(H\left\{v_{i}\left(k\right)\right\}\to H\left\{v_{j}\left(k\right)\right\}|G,\beta \right)=\beta$. The effective superedge transmission probability in the CGN is $\beta_{w_{\mathrm{ij}}\left(k\right)}=1-{\left(1-\beta \right)}^{1}=\beta$, yielding identical transmission probabilities on both representations.

Case 2: $e_{\mathrm{ij}}^{w}\left(k\right)>1$ (multiedge coupling). Here, the two subgraphs are connected by $e_{\mathrm{ij}}^{w}\left(k\right)$ independent edges. Because all nodes in $H\left\{v_{i}\left(k\right)\right\}$ are infected once $v_{i}\left(k\right)$ is infected, the probability that infection crosses to $H\left\{v_{j}\left(k\right)\right\}$ through at least one of these edges is

$$P\left(H\left\{v_{i}\left(k\right)\right\}\to H\left\{v_{j}\left(k\right)\right\}|G,\beta \right)=1-{\left(1-\beta \right)}^{e_{\mathrm{ij}}^{w}\left(k\right)}$$ (33)

By construction, this is exactly the effective transmission probability $\beta_{w_{\mathrm{ij}}\left(k\right)}$ assigned to the superedge in $G_{R=k}$

$$P\left(v_{i}\left(k\right)\to v_{j}\left(k\right)|G_{R=k},\beta_{w_{\mathrm{ij}}\left(k\right)}\right)=\beta_{w_{\mathrm{ij}}\left(k\right)}$$ (34)

In both cases, the probability of contagion transmission between subgraphs in the original network is exactly reproduced by the corresponding superedge in the CGN. This establishes the equivalence stated in Eq. 32.

## Supplementary Materials

### This PDF file includes:

Sections S1 to S3 Table S1 Figs. S1 to S19 References

## References

[1] R. Pastor-Satorras, C. Castellano, P. Van Mieghem, A. Vespignani, Epidemic processes in complex networks. Rev. Mod. Phys. 87, 925–979 (2015).

[2] Z.-K. Zhang, C. Liu, X.-X. Zhan, X. Lu, C.-X. Zhang, Y.-C. Zhang, Dynamics of information diffusion and its applications on complex networks. Phys. Rep. 651, 1–34 (2016).

[3] F. Battiston, G. Cencetti, I. Iacopini, V. Latora, M. Lucas, A. Patania, J.-G. Young, G. Petri, Networks beyond pairwise interactions: Structure and dynamics. Phys. Rep. 874, 1–92 (2020).

[4] R. Pastor-Satorras, A. Vespignani, Epidemic spreading in scale-free networks. Phys. Rev. Lett. 86, 3200–3203 (2001). 10.1103/PhysRevLett.86.3200 11290142

[5] V. Colizza, A. Barrat, M. Barthélemy, A. Vespignani, The role of the airline transportation network in the prediction and predictability of global epidemics. Proc. Natl. Acad. Sci. U.S.A. 103, 2015–2020 (2006). 10.1073/pnas.0510525103 PMC1413717 16461461

[6] J. L. Iribarren, E. Moro, Impact of human activity patterns on the dynamics of information diffusion. Phys. Rev. Lett. 103, 038702 (2009). 10.1103/PhysRevLett.103.038702 19659326

[7] J. T. Davis, N. Perra, Q. Zhang, Y. Moreno, A. Vespignani, Phase transitions in information spreading on structured populations. Nat. Phys. 16, 590–596 (2020).

[8] C. Nardini, B. Kozma, A. Barrat, Who’s talking first? Consensus or lack thereof in coevolving opinion formation models. Phys. Rev. Lett. 100, 158701 (2008). 10.1103/PhysRevLett.100.158701 18518162

[9] H. Schawe, L. Hernández, Higher order interactions destroy phase transitions in Deffuant opinion dynamics model. Commun. Phys. 5, 32 (2022).

[10] D. Brockmann, D. Helbing, The hidden geometry of complex, network-driven contagion phenomena. Science 342, 1337–1342 (2013). 10.1126/science.1245200 24337289

[11] G. Burgio, A. Arenas, S. Gómez, J. T. Matamalas, Network clique cover approximation to analyze complex contagions through group interactions. Commun. Phys. 4, 111 (2021).

[12] Y. Moreno, R. Pastor-Satorras, A. Vespignani, Epidemic outbreaks in complex heterogeneous networks. Eur. Phys. J. B 26, 521–529 (2002).

[13] C. Castellano, R. Pastor-Satorras, Thresholds for epidemic spreading in networks. Phys. Rev. Lett. 105, 218701 (2010). 10.1103/PhysRevLett.105.218701 21231361

[14] M. E. Newman, Spread of epidemic disease on networks. Phys. Rev. E 66, 016128 (2002). 10.1103/PhysRevE.66.016128 12241447

[15] E. Volz, SIR dynamics in random networks with heterogeneous connectivity. J. Math. Biol. 56, 293–310 (2008). 10.1007/s00285-007-0116-4 PMC7080148 17668212

[16] B. Karrer, M. E. Newman, Message passing approach for general epidemic models. Phys. Rev. E 82, 016101 (2010). 10.1103/PhysRevE.82.016101 20866683

[17] A. Y. Lokhov, M. Mézard, L. Zdeborová, Dynamic message-passing equations for models with unidirectional dynamics. Phys. Rev. E 91, 012811 (2015). 10.1103/PhysRevE.91.012811 25679661

[18] G. T. Cantwell, M. E. Newman, Message passing on networks with loops. Proc. Natl. Acad. Sci. U.S.A. 116, 23398–23403 (2019). 10.1073/pnas.1914893116 PMC6876225 31685640

[19] M. Girvan, M. E. Newman, Community structure in social and biological networks. Proc. Natl. Acad. Sci. U.S.A. 99, 7821–7826 (2002). 10.1073/pnas.122653799 PMC122977 12060727

[20] C. Song, S. Havlin, H. A. Makse, Self-similarity of complex networks. Nature 433, 392–395 (2005). 10.1038/nature03248 15674285

[21] M. Sales-Pardo, R. Guimera, A. A. Moreira, L. A. N. Amaral, Extracting the hierarchical organization of complex systems. Proc. Natl. Acad. Sci. U.S.A. 104, 15224–15229 (2007). 10.1073/pnas.0703740104 PMC2000510 17881571

[22] M. A. Serrano, M. Boguñá, A. Vespignani, Extracting the multiscale backbone of complex weighted networks. Proc. Natl. Acad. Sci. U.S.A. 106, 6483–6488 (2009). 10.1073/pnas.0808904106 PMC2672499 19357301

[23] S. Xu, P. Wang, “Coarse graining of complex networks: A k-means clustering approach,” in *Proceedings of the 28th Chinese Control and Decision Conference, CCDC 2016* (IEEE, 2016), pp. 4113–4118.

[24] G. García-Pérez, M. Boguñá, M. Serrano, Multiscale unfolding of real networks by geometric renormalization. Nat. Phys. 14, 583–589 (2018).

[25] D. Grady, C. Thiemann, D. Brockmann, Robust classification of salient links in complex networks. Nat. Commun. 3, 864 (2012). 10.1038/ncomms1847 22643891

[26] L. M. Shekhtman, J. P. Bagrow, D. Brockmann, Robustness of skeletons and salient features in networks. J. Complex Netw. 2, 110–120 (2014).

[27] A. Mercier, S. Scarpino, C. Moore, Effective resistance against pandemics: Mobility network sparsification for high-fidelity epidemic simulations. PLOS Comput. Biol. 18, e1010650 (2022). 10.1371/journal.pcbi.1010650 PMC9681106 36413581

[28] T. P. Peixoto, L. Gauvin, Change points, memory and epidemic spreading in temporal networks. Sci. Rep. 8, 15511 (2018). 10.1038/s41598-018-33313-1 PMC6195572 30341364

[29] M. De Domenico, V. Nicosia, A. Arenas, V. Latora, Structural reducibility of multilayer networks. Nat. Commun. 6, 6864 (2015). 10.1038/ncomms7864 25904309

[30] D. Gfeller, P. De Los Rios, Spectral coarse graining of complex networks. Phys. Rev. Lett. 99, 038701 (2007). 10.1103/PhysRevLett.99.038701 17678338

[31] D. Gfeller, P. De Los Rios, Spectral coarse graining and synchronization in oscillator networks. Phys. Rev. Lett. 100, 174104 (2008). 10.1103/PhysRevLett.100.174104 18518293

[32] V. Thibeault, G. St-Onge, L. J. Dubé, P. Desrosiers, Threefold way to the dimension reduction of dynamics on networks: An application to synchronization. Phys. Rev. Res. 2, 043215 (2020).

[33] E. Laurence, N. Doyon, L. J. Dubé, P. Desrosiers, Spectral dimension reduction of complex dynamical networks. Phys. Rev. X 9, 011042 (2019).

[34] S. Ghosh, P. Khanra, P. Kundu, P. Ji, D. Ghosh, C. Hens, Dimension reduction in higher-order contagious phenomena. Chaos 33, 053117 (2023). 10.1063/5.0152959 37229635

[35] J. Gao, Intrinsic simplicity of complex systems. Nat. Phys. 20, 184–185 (2024).

[36] A. J. Allen, C. Moore, L. Hébert-Dufresne, Compressing the chronology of a temporal network with graph commutators. Phys. Rev. Lett. 132, 077402 (2024). 10.1103/PhysRevLett.132.077402 PMC11223189 38427895

[37] V. Thibeault, A. Allard, P. Desrosiers, The low-rank hypothesis of complex systems. Nat. Phys. 20, 294–302 (2024).

[38] Y. Chen, G. Paul, S. Havlin, F. Liljeros, H. E. Stanley, Finding a better immunization strategy. Phys. Rev. Lett. 101, 058701 (2008). 10.1103/PhysRevLett.101.058701 18764435

[39] C. M. Schneider, T. Mihaljev, S. Havlin, H. J. Herrmann, Suppressing epidemics with a limited amount of immunization units. Phys. Rev. E 84, 061911 (2011). 10.1103/PhysRevE.84.061911 22304120

[40] M. Kitsak, L. K. Gallos, S. Havlin, F. Liljeros, L. Muchnik, H. E. Stanley, H. A. Makse, Identification of influential spreaders in complex networks. Nat. Phys. 6, 888–893 (2010).

[41] L. Xue, P. Zhang, A. Zeng, Maximizing spreading in complex networks with risk in node activation. Inf. Sci. 586, 1–23 (2022).

[42] R. A. Rossi, N. K. Ahmed, “The network data repository with interactive graph analytics and visualization,” in *Proceedings of the 29th AAAI Conference on Artificial Intelligence* (AAAI Press, 2015).

[43] T. Yabe, K. Tsubouchi, T. Shimizu, Y. Sekimoto, K. Sezaki, E. Moro, A. Pentland, YJMob100K: City scale and longitudinal dataset of anonymized human mobility trajectories. Sci. Data 11, 397 (2024). 10.1038/s41597-024-03237-9 PMC11026376 38637602

[44] P. Holme, Three faces of node importance in network epidemiology: Exact results for small graphs. Phys. Rev. E 96, 062305 (2017). 10.1103/PhysRevE.96.062305 PMC7217518 29347435

[45] W. Chen, Y. Wang, S. Yang, “Efficient influence maximization in social networks,” in *Proceedings of the 15th ACM SIGKDD International Conference on Knowledge Discovery Data Mining* (Association for Computing Machinery, 2009), pp. 199–208.

[46] F. Morone, H. A. Makse, Influence maximization in complex networks through optimal percolation. Nature 524, 65–68 (2015). 10.1038/nature14604 26131931

[47] P. Bonacich, Some unique properties of eigenvector centrality. Soc. Netw. 29, 555–564 (2007).

[48] J. Zhan, S. Gurung, S. P. K. Parsa, Identification of top-K nodes in large networks using Katz centrality. J. Big Data 4, 16 (2017).

[49] E. Estrada, J. A. Rodriguez-Velazquez, Subgraph centrality in complex networks. Phys. Rev. E 71, 056103 (2005). 10.1103/PhysRevE.71.056103 16089598

[50] T. Martin, X. Zhang, M. E. Newman, Localization and centrality in networks. Phys. Rev. E 90, 052808 (2014). 10.1103/PhysRevE.90.052808 25493835

[51] E. Colman, P. Holme, H. Sayama, C. Gershenson, Efficient sentinel surveillance strategies for preventing epidemics on networks. PLOS Comput. Biol. 15, e1007517 (2019). 10.1371/journal.pcbi.1007517 PMC6910701 31765382

[52] J. L. Herrera, R. Srinivasan, J. S. Brownstein, A. P. Galvani, L. A. Meyers, Disease surveillance on complex social networks. PLOS Comput. Biol. 12, e1004928 (2016). 10.1371/journal.pcbi.1004928 PMC4944951 27415615

[53] G. St-Onge, J.-G. Young, L. Hébert-Dufresne, L. J. Dubé, Efficient sampling of spreading processes on complex networks using a composition and rejection algorithm. Comput. Phys. Commun. 240, 30–37 (2019). 10.1016/j.cpc.2019.02.008 PMC6839824 31708586

[54] J. C. Miller, Epidemics on networks with large initial conditions or changing structure. PLOS ONE 9, e101421 (2014). 10.1371/journal.pone.0101421 PMC4086930 25004149

[55] E. Kenah, J. M. Robins, Second look at the spread of epidemics on networks. Phys. Rev. E 76, 036113 (2007). 10.1103/PhysRevE.76.036113 PMC2215389 17930312

[56] I. Iacopini, G. Petri, A. Barrat, V. Latora, Simplicial models of social contagion. Nat. Commun. 10, 2485 (2019). 10.1038/s41467-019-10431-6 PMC6554271 31171784

[57] F. Battiston, E. Amico, A. Barrat, G. Bianconi, G. Ferraz de Arruda, B. Franceschiello, I. Iacopini, S. Kéfi, V. Latora, Y. Moreno, M. M. Murray, T. P. Peixoto, F. Vaccarino, G. Petri, The physics of higher-order in teractions in complex systems. Nat. Phys. 17, 1093–1098 (2021).

[58] G. Ferraz de Arruda, A. Aleta, Y. Moreno, Contagion dynamics on higher-order networks. Nat. Rev. Phys. 6, 468–482 (2024).

[59] Y. Zhang, M. Lucas, F. Battiston, Higher-order interactions shape collective dynamics differently in hypergraphs and simplicial complexes. Nat. Commun. 14, 1605 (2023). 10.1038/s41467-023-37190-9 PMC10036330 36959174

[60] B. Gross, D. Vaknin, S. V. Buldyrev, S. Havlin, Two transitions in spatial modular networks. New J. Phys. 22, 053002 (2020).

[61] U. Alvarez-Rodriguez, F. Battiston, G. F. de Arruda, Y. Moreno, M. Perc, V. Latora, Evolutionary dynamics of higher-order interactions in social networks. Nat. Hum. Behav. 5, 586–595 (2021). 10.1038/s41562-020-01024-1 33398148

[62] F. Radicchi, Predicting percolation thresholds in networks. Phys. Rev. E 91, 010801 (2015). 10.1103/PhysRevE.91.010801 25679556

[63] J. Leskovec, A. Krevl, SNAP Datasets: Stanford large network dataset collection (2014); http://snap.stanford.edu/data.

[64] A.-L. Barabási, *Network Science* (Cambridge Univ. Press, 2016).

[65] Network Repository, Network Repository. An Interactive Scientific Network Data Repository, https://networkrepository.com/.

[66] B. Rozemberczki, Datasets, GitHub, https://github.com/benedekrozemberczki/datasets.

[67] Stanford University, Stanford Network Analysis Project, http://snap.stanford.edu/data/.

[68] ICON, Index of Complex Networks, https://icon.colorado.edu/#!/networks.

[69] A.-L. Barabási, Network Science: Data (2016); http://networksciencebook.com/translations/en/resources/data.html.

