# Supplementary material for: Contagion-preserving compression for multiscale epidemic modeling
Source: Sci Adv. 2026 Oct 9;12(41):eadz4710. doi: 10.1126/sciadv.adz4710 (PMC13651889; doi:10.1126/sciadv.adz4710)
Supplement: Supplementary file 1 [file sciadv.adz4710_sm.pdf]

Science Advances
AAAS

Supplementary Materials for

Contagion-preserving compression for multiscale epidemic modeling

Leyang Xue *et al.*

Corresponding author: An Zeng, anzeng@bnu.edu.cn

Sci. Adv. **12**, eadz4710 (2026)
DOI: 10.1126/sciadv.adz4710

This PDF file includes:

Sections S1 to S3
Table S1
Figs. S1 to S19
References

S1. NETWORKS

We apply the ISCG framework to a diverse set of real-world networks sourced from multiple publicly available repositories: (1) Network Repository[65] - NetRes *(42)*; (2) GitHub[66]; (3) Stanford Network Analysis Project[67] - SNAP *(63)*; (4) Index of Complex Networks[68] - ICN; (5) Network Science by Albert-László Barabási[69] - Barabasi *(64)*. These datasets span multiple domains—including scientific collaboration, communication, and social media—offering diverse topological structures for evaluating the generalizability of the ISCG framework. In each network, nodes represent individual entities (e.g., scientists, email accounts), and edges encode pairwise interactions or relational links. To ensure that contagion processes can percolate throughout the network, we extract and analyze only the largest connected component (LCC) from each dataset. Summary statistics and structural properties of all networks are provided in Supplementary Table I.

S2. SUPPLEMENTARY METHODS

S2.1. Transmission probability

In coarse-grained networks (CGNs), super-edges between super-nodes are assigned weights based on the number of original edges they aggregate. To preserve the fidelity of contagion dynamics under coarse-graining, it is essential to appropriately map these weights to effective transmission probabilities. This ensures that the aggregated structure accurately reflects the underlying transmission likelihood between groups of nodes.

Mapping edge weights to transmission probabilities. The effective infection probability $\beta_{w_{ij}(k)}$ for a super-edge with weight $w_{ij}(k)$ in a k-clique CGN is given by:

$$\beta_{w_{ij}(k)} = 1 - (1-\beta)^{w_{ij}(k)}, \tag{S1}$$

where β is the infection probability associated with a single edge in the original network. This formulation accounts for the union of $w_{ij}(k)$ independent transmission attempts, reflecting the cumulative probability that at least one pathway successfully transmits the contagion. It preserves the probabilistic equivalence between the original and coarse-grained representations.

An illustrative example is provided in Supplementary Figure 1**Aa**, where the edge between nodes i and j in a 4-clique CGN has a weight of 2, indicating the presence of two distinct transmission pathways in the original network. Applying the mapping yields an effective transmission probability $\beta_{w_{ij}(k)}$, which captures the cumulative likelihood that at least one of these independent pathways successfully transmits the contagion. This weighted mapping is fundamental to the ISCG framework. By assigning infection probabilities that accurately reflect the multiplicity of underlying interactions, the CGN retains the essential dynamics of the original network while enabling substantial reductions in computational and memory complexity. It thereby ensures that coarse-grained simulations remain faithful to the full contagion process, even at large scales.

S2.2. Recursive framework for computing complete infection probability in k-cliques

We develop a recursive framework to compute the probability that a contagion, initiated by a single infected node within a fully connected k-clique, eventually infects all other nodes in the clique. This probability is denoted by $\Lambda^k_{1\to(k-1)}(\beta)$, where β is the per-edge transmission probability. The key idea is to exploit the symmetry and completeness of clique structures: once a subset of nodes becomes infected, subsequent transmission within the remaining clique depends only on the number of infected and susceptible nodes, not on their identities. This allows the full infection probability to be constructed hierarchically from lower-order cliques, while accounting for all possible transmission cascades.

S2.2.1. Recursive formulation

To evaluate $\Lambda^k_{1\to(k-1)}(\beta)$, we decompose the infection process into successive transmission stages. Starting from a single infected seed, the first step consists of direct transmissions from the seed to its $k-1$ neighbors. Conditioned on the number of successful transmissions in this step, the remaining dynamics reduce to a smaller clique infection problem. This leads to the recurrence relation

$$\Lambda^k_{1\to(k-1)}(\beta) = \sum_{i=1}^{k-1} \binom{k-1}{i} \beta^i (1-\beta)^{k-1-i} \Lambda^{k-1}_{i\to(k-1-i)}(\beta), \tag{S2}$$

where:

- i is the number of nodes directly infected by the initial seed in the first transmission step;
- $\binom{k-1}{i}$ counts the number of ways to select these i nodes from the remaining $k-1$;
- $\beta^i(1-\beta)^{k-1-i}$ is the probability of exactly i successful transmissions and $k-1-i$ failures;
- $\Lambda^{k-1}_{i\to(k-1-i)}(\beta)$ is the probability that the i newly infected nodes jointly infect the remaining $k-1-i$ susceptible nodes within the induced $(k-1)$-clique.

This recursive structure explicitly enumerates all feasible infection pathways while avoiding overcounting. By iteratively reducing the problem size, it provides an exact and scalable method for computing full-infection probabilities in dense subgraphs, and forms the analytical basis for the clique-level saturation thresholds used throughout the paper.

S2.2.2. Example calculations for small cliques

To illustrate the recursive framework, we provide explicit calculations for cliques of size $k=2$ through $k=5$. These examples make transparent how the recursion enumerates all possible transmission pathways within fully connected subgraphs.

For a 2-clique—two nodes connected by a single edge—the probability of complete infection is trivial:

$$\Lambda^2_{1\to1}(\beta) = \binom{1}{1}\beta\Lambda^1_{1\to0}(\beta) = \beta. \tag{S3}$$

Supplementary Table I. **Structural properties of real-world networks used in this study.** For each network, we report the following characteristics: network name; number of nodes (N); number of edges (E); average degree ($\langle k \rangle$); average clustering coefficient ($\langle c \rangle$); degree assortativity coefficient (r_a); and degree exponent (γ), estimated via maximum likelihood fitting. We also report the k-clique exponent in the original network (α_k), and the k-clique exponent $\alpha_{\Delta k}$ characterizing the distribution of cliques generated during the coarse-graining process. The final column lists the source repository for each dataset.

**Network**	N	E	$\langle k \rangle$	$\langle c \rangle$	r_a	γ	α_k	$\alpha_{\Delta k}$	**Source**
AstroPh	17,903	196,972	22.00	0.63	0.20	4.50	-7.36	-3.00	NetRes
Bio-celegans	453	2,025	8.94	0.65	-0.23	2.60	-11.18	-5.50	NetRes
Bio-wormnet	2,274	78,328	68.89	0.85	0.30	4.78	-3.62	-3.04	NetRes
CondMat	21,363	91,286	8.55	0.64	0.13	3.35	-6.50	-4.03	NetRes
DBLP	12,495	49,563	7.93	0.12	-0.05	3.35	-5.39	-4.91	NetRes
Deezer-europe	28,281	92,752	6.56	0.14	0.10	4.86	-8.13	-6.10	GitHub
Email-Enron	33,696	180,811	10.73	0.51	-0.12	1.97	-11.40	-7.08	NetRes
GrQc	4,158	13,422	6.46	0.56	0.64	2.04	-3.78	-5.84	NetRes
HepPh	11,204	117,619	21.00	0.62	0.63	2.08	-2.32	-3.85	NetRes
Inf-openflights	2,905	15,645	10.77	0.46	0.05	1.71	-8.06	-5.40	NetRes
Internet	190,914	607,610	6.37	0.16	0.02	3.40	-11.75	-11.74	Barabasi
Musae-DE	9,498	153,138	32.25	0.20	-0.12	2.55	-11.73	-7.28	SNAP
Musae-facebook	22,470	170,823	15.20	0.36	0.08	3.19	-8.18	-4.04	SNAP
Musae-git	37,700	289,003	15.33	0.17	-0.08	2.54	-8.72	-5.89	SNAP
NetSci2019	32,904	296,876	18.04	0.80	0.99	2.02	-4.34	-4.36	ICN
Phonecalls	30,420	52,841	3.47	0.15	0.17	4.71	-5.67	-8.12	Barabasi
Power-eris	1,174	8,687	14.80	0.43	0.89	1.67	-3.12	-7.13	SNAP
Power-bus	685	1,282	3.74	0.17	0.18	6.26	-6.27	-6.09	SNAP
Tech-pgp	10,680	24,316	4.55	0.27	0.24	4.26	-2.13	-4.98	SNAP
USairport2010	1,572	17,214	21.90	0.50	-0.11	1.85	-7.07	-4.96	ICN
WWW	325,729	1,090,108	6.69	0.23	-0.05	2.15	-4.42	-3.33	Barabasi

since infection can occur only through the single available link.

For a 3-clique, two distinct infection pathways contribute to complete contagion, as illustrated in Supplementary Fig. 1(Bb): (i) the initially infected node transmits to both neighbors in the first step; (ii) it infects exactly one neighbor, which then infects the remaining node in a subsequent step. Accounting for both possibilities yields

$$\begin{aligned}\Lambda^3_{1\to2}(\beta) &= \binom{2}{2}\beta^2(1-\beta)^0\Lambda^2_{2\to0}(\beta) + \binom{2}{1}\beta(1-\beta)\Lambda^2_{1\to1}(\beta)\\ &= -2\beta^3 + 3\beta^2.\end{aligned} \tag{S4}$$

For a 4-clique, we condition on the number of nodes infected by the initial seed in the first transmission step. Specifically, the seed may infect one, two, or all three of the remaining nodes. Each case is resolved recursively, as illustrated in Supplementary Fig. 1(Bc):

$$\begin{aligned}\Lambda^4_{1\to3}(\beta) &= \binom{3}{3}\beta^3(1-\beta)^0\Lambda^3_{3\to0}(\beta)\\ &+ \binom{3}{2}\beta^2(1-\beta)\Lambda^3_{2\to1}(\beta) + \binom{3}{1}\beta(1-\beta)^2\Lambda^3_{1\to2}(\beta)\\ &= -6\beta^6 + 24\beta^5 - 33\beta^4 + 16\beta^3.\end{aligned} \tag{S5}$$

Here, $\Lambda^3_{2\to1}(\beta)$ denotes the probability that two infected nodes in a 3-clique collectively infect the remaining susceptible node. This quantity is evaluated via the complementary probability that all transmission attempts fail:

$$\Lambda^3_{2\to1}(\beta) = 1 - \binom{1}{0}\Lambda^2_{2\to0}(\beta)(1-\beta)^{2\times1}(1-\beta)^{0\times1}.$$

Extending the same procedure to a 5-clique (Supplementary Fig. 1(Bd)), we obtain:

$$\begin{aligned}\Lambda^5_{1\to4}(\beta) &= \binom{4}{4}\beta^4(1-\beta)^0\Lambda^4_{4\to0}(\beta) + \binom{4}{3}\beta^3(1-\beta)\Lambda^4_{3\to1}(\beta)\\ &+ \binom{4}{2}\beta^2(1-\beta)^2\Lambda^4_{2\to2}(\beta) + \binom{4}{1}\beta(1-\beta)^3\Lambda^4_{1\to3}(\beta)\\ &= 24\beta^{10} - 180\beta^9 + 570\beta^8 - 980\beta^7 + 970\beta^6\\ &\quad - 528\beta^5 + 125\beta^4.\end{aligned} \tag{S6}$$

In this expression, $\Lambda^4_{3\to1}(\beta)$ represents the probability that three infected nodes jointly infect the remaining susceptible node in a 4-clique, given by

$$\Lambda^4_{3\to1}(\beta) = 1 - \Lambda^3_{3\to0}(\beta)(1-\beta)^3.$$

Similarly, $\Lambda^4_{2\to2}(\beta)$ accounts for the probability that two infected nodes succeed in infecting the remaining two susceptible nodes:

$$\begin{aligned}\Lambda^4_{2\to2}(\beta) = 1 - \Bigg[&\binom{2}{0}\Lambda^2_{2\to0}(\beta)(1-\beta)^4\\ &+ \binom{2}{1}\Lambda^3_{2\to1}(\beta)(1-\beta)^3\Bigg].\end{aligned}$$

These examples demonstrate how complete infection probabilities for larger cliques are constructed systematically by conditioning on early transmission events and recursively resolving the remaining subproblems.

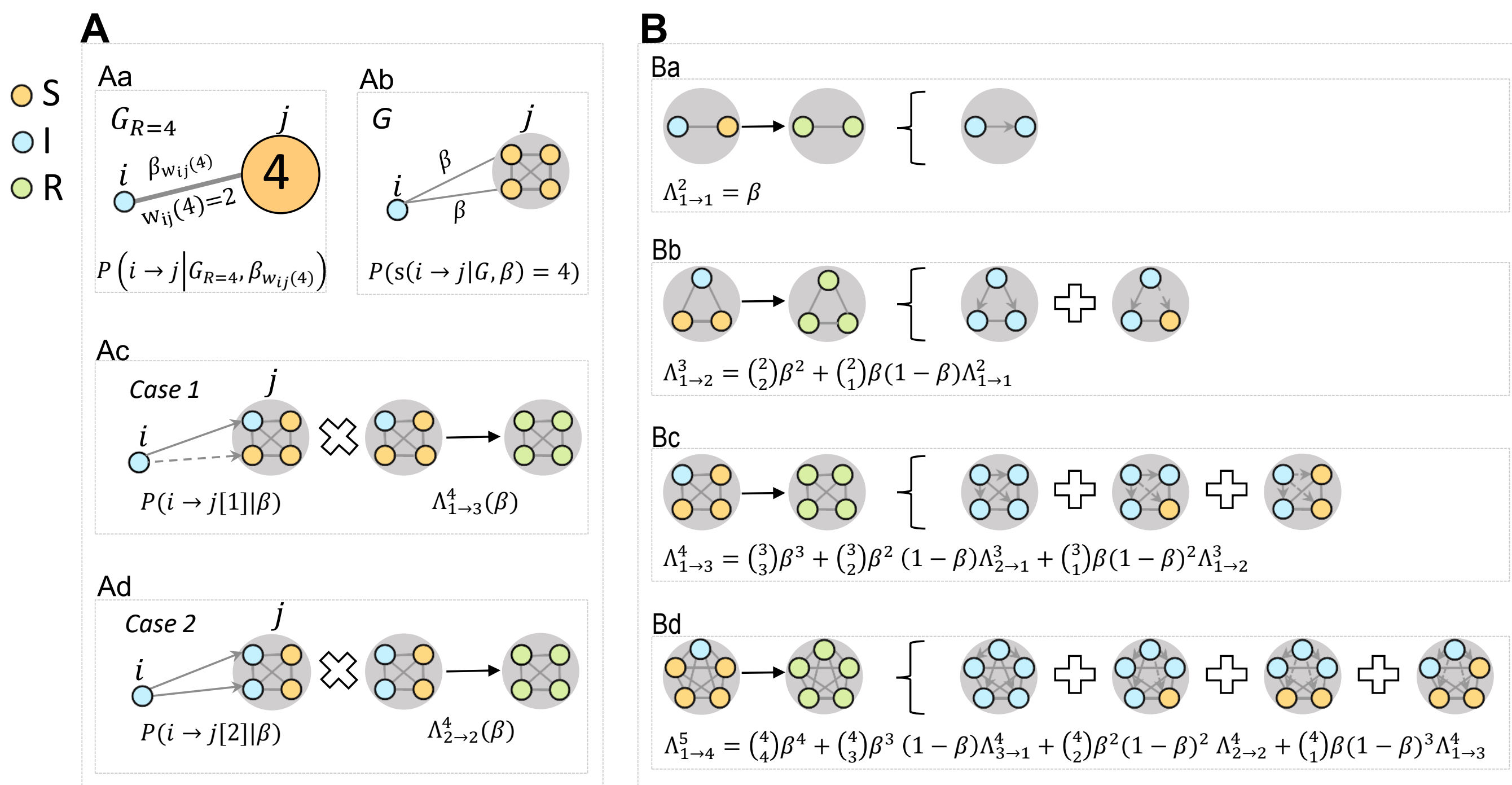

Supplementary Figure 1. **Illustration of infection probabilities in coarse-grained networks (CGNs) and k-clique structures.** **A**, Infection probability mapping in a 4-clique CGN. **Aa**, Schematic of a 4-clique CGN, where super-node i transmits infection to super-node j via a weighted edge ($e^w_{ij} = 2$). **Ab**, Corresponding configuration in the original network, where infection reaches all nodes in the subgraph associated with $v_j(k)$ once any node is infected. **Ac**, Case 1: Node i infects a single node in j's clique; contagion then propagates internally to the remaining nodes. **Ad**, Case 2: Node i infects two nodes simultaneously in j's clique, which jointly trigger full contagion within the subgraph. **B**, Infection dynamics in k-clique structures for $k = 2$ to 5. **Ba–Bd**, For each k, we show how a single seed node can lead to complete contagion within the clique. Each panel illustrates the minimal configurations required to achieve full spread. Solid gray arrows denote successful transmission events, while dashed gray arrows indicate failed attempts. Left-hand diagrams depict the final fully infected clique; right-hand diagrams enumerate the possible transmission pathways.

S2.2.3. Extending to larger cliques

The recursive formulation introduced above extends straightforwardly to cliques of arbitrary size. Contagion within a k-clique unfolds hierarchically: an initially infected node infects a subset of its neighbors, after which the newly infected nodes continue the spreading among the remaining susceptible ones. As a result, the probability of complete infection in a k-clique can be expressed recursively in terms of infection probabilities of smaller cliques.

As an example, the probability that a 6-clique becomes fully infected from a single initially infected node is

$$\begin{aligned}\Lambda^6_{1\to5}(\beta) &= \sum_{i=1}^{5} \binom{5}{i} \beta^i (1-\beta)^{5-i} \Lambda^5_{i\to(5-i)}(\beta) \\ &= 120\beta^{15} - 1440\beta^{14} + 7830\beta^{13} - 25440\beta^{12} \\ &\quad + 54780\beta^{11} - 81840\beta^{10} + 86110\beta^9 - 63195\beta^8 \\ &\quad + 31080\beta^7 - 9300\beta^6 + 1296\beta^5,\end{aligned} \tag{S7}$$

where each term $\Lambda^5_{i\to(5-i)}(\beta)$ corresponds to a subconfiguration involving i initially infected nodes in the underlying 5-clique and is evaluated recursively.

Applying the same construction to a 7-clique yields

$$\begin{aligned}\Lambda^7_{1\to6}(\beta) &= \sum_{i=1}^{6} \binom{6}{i} \beta^i (1-\beta)^{6-i} \Lambda^6_{i\to(6-i)}(\beta) \\ &= -720\beta^{21} + 12600\beta^{20} - 103320\beta^{19} + 526890\beta^{18} \\ &\quad - 1869840\beta^{17} + 4894680\beta^{16} - 9770810\beta^{15} \\ &\quad + 15159120\beta^{14} - 18448710\beta^{13} + 17633945\beta^{12} \\ &\quad - 13150032\beta^{11} + 7527471\beta^{10} - 3209430\beta^9 \\ &\quad + 965160\beta^8 - 183810\beta^7 + 16807\beta^6,\end{aligned} \tag{S8}$$

where each $\Lambda^6_{i\to(6-i)}(\beta)$ is obtained from the previously derived 6-clique expressions. For clarity, consider the subcase $\Lambda^6_{5\to1}(\beta)$, corresponding to five infected nodes transmitting to the single remaining susceptible node in a 6-clique. This probability is given by

$$\Lambda^6_{5\to1}(\beta) = 1 - \binom{1}{0} \Lambda^5_{5\to0}(\beta)(1-\beta)^{5\times1}(1-\beta)^{0\times1}.$$

which simply reflects the complement of the event that all five infected nodes fail to transmit across their independent edges.

The recursive construction provides a systematic and scalable route for computing full infection probabilities in k-cliques of increasing size. By iteratively applying the expressions derived for smaller cliques, the probability of complete infection in a k-clique can be obtained exactly as a polynomial in the transmission probability β. While these expressions rapidly grow in degree and complexity with k, they encode the full combinatorics of intra-clique transmission pathways and thus provide an exact characterization of contagion saturation in densely connected subgraphs. For an 8-clique, 9-clique, 10-clique, the probability that a single initially infected node leads to full infection of the remaining nodes is

$$
\begin{aligned}
\Lambda^{8}_{1\to 7}(\beta) = {} & \sum_{i=1}^{7} \binom{7}{i} \beta^{i} (1-\beta)^{7-i} \Lambda^{7}_{i\to 7-i}(\beta) \\
= {} & -5040\beta^{28} + 120960\beta^{27} - 1386000\beta^{26} + 10086720\beta^{25} - 52319190\beta^{24} + 205732800\beta^{23} \\
& - 636845160\beta^{22} + 1590501640\beta^{21} - 3258291120\beta^{20} + 5536123600\beta^{19} - 7856193296\beta^{18} + 9345271992\beta^{17} \\
& - 9324568001\beta^{16} + 7786027816\beta^{15} - 5410382880\beta^{14} + 3098951072\beta^{13} - 1441519296\beta^{12} + 532354536\beta^{11} \\
& - 150657080\beta^{10} + 30802240\beta^{9} - 4068456\beta^{8} + 262144\beta^{7}
\end{aligned}
\tag{S9}
$$

$$
\begin{aligned}
\Lambda^{9}_{1\to 8}(\beta) = {} & \sum_{i=1}^{8} \binom{8}{i} \beta^{i} (1-\beta)^{8-i} \Lambda^{8}_{i\to 8-i}(\beta) \\
= {} & 40320\beta^{36} - 1270080\beta^{35} + 19323360\beta^{34} - 189090720\beta^{33} + 1337084280\beta^{32} - 7276802400\beta^{31} \\
& + 31699531080\beta^{30} - 113494862880\beta^{29} + 340283488860\beta^{28} - 866124834680\beta^{27} + 1890561025584\beta^{26} \\
& - 3565563257088\beta^{25} + 5841996257286\beta^{24} - 8346992077872\beta^{23} + 10424251015920\beta^{22} - 11390487268104\beta^{21} \\
& + 10886907107538\beta^{20} - 9087542239104\beta^{19} + 6604921452864\beta^{18} - 4160649753288\beta^{17} + 2256642859464\beta^{16} \\
& - 1044267039720\beta^{15} + 407158789500\beta^{14} - 131447465856\beta^{13} + 34273176588\beta^{12} - 6952118544\beta^{11} \\
& + 1032288516\beta^{10} - 100143792\beta^{9} + 4782969\beta^{8}
\end{aligned}
\tag{S10}
$$

$$
\begin{aligned}
\Lambda^{10}_{1\to 9}(\beta) = {} & \sum_{i=1}^{9} \binom{9}{i} \beta^{i} (1-\beta)^{9-i} \Lambda^{9}_{i\to 9-i}(\beta) \\
= {} & 362880\beta^{45} - 14515200\beta^{44} + 282592800\beta^{43} - 3567715200\beta^{42} + 32833495800\beta^{41} \\
& - 234748765440\beta^{40} + 1357020856800\beta^{39} - 6517548349200\beta^{38} + 26521978127400\beta^{37} \\
& - 92792729053500\beta^{36} + 282287441908080\beta^{35} - 753273866698920\beta^{34} \\
& + 1775448575926410\beta^{33} - 3716558335019880\beta^{32} + 6939551178972720\beta^{31} \\
& - 11596879696617600\beta^{30} + 17388982649046960\beta^{29} - 23437879996999860\beta^{28} \\
& + 28429756177413360\beta^{27} - 31050312703343640\beta^{26} + 30532209914200806\beta^{25} \\
& - 27011077082801580\beta^{24} + 21469710851551800\beta^{23} - 15300758477189520\beta^{22} \\
& + 9748958193896580\beta^{21} - 5532426738592740\beta^{20} + 2782630494934920\beta^{19} \\
& - 1232671556293800\beta^{18} + 477077447178540\beta^{17} - 159642667620135\beta^{16} \\
& + 45558310696800\beta^{15} - 10884316965480\beta^{14} + 2121183237600\beta^{13} \\
& - 324496267200\beta^{12} + 36628300800\beta^{11} - 2719892160\beta^{10} + 100000000\beta^{9}
\end{aligned}
\tag{S11}
$$

Each term $\Lambda^{k}_{i\to(k-i)}(\beta)$ denotes the probability that a group of i infected nodes jointly transmits the contagion to the remaining $k-i$ susceptible nodes within a k-clique. These probabilities are obtained recursively from lower-order cliques, so that higher-order contagion dynamics are built compositionally from simpler infection events. This hierarchical construction preserves the full combinatorial structure of intra-clique transmission while enabling exact evaluation of saturation behavior in dense subgraphs.

S2.2.4. Threshold for complete contagion in k-cliques

To ensure full infection within a k-clique, we define the critical transmission threshold $\hat{\beta}_k$ as the minimum value of β for which the infection probability satisfies $\Lambda^k_{1\to(k-1)}(\beta) = 1$. At this threshold, a single infected node is guaranteed to transmit the contagion to all other nodes in the clique with certainty. The parameter $\hat{\beta}_k$ thus serves as a key benchmark for the feasibility of complete local spreading and is foundational for preserving dynamic fidelity in the ISCG framework. Conceptually, $\hat{\beta}_k$ parallels the fixation threshold in stochastic birth-death processes, marking the point at which a system reaches an absorbing state. Analogously, when $\beta \geq \hat{\beta}_k$, the infection process becomes deterministically complete within the clique, ensuring that the coarse-grained representation remains dynamically equivalent to the original. By identifying and applying $\hat{\beta}_k$ in the construction of coarse-grained networks, the ISCG framework guarantees consistency in contagion dynamics across scales. This threshold condition plays a central role in aligning local transmission properties with the global behavior of the system, thereby supporting accurate and scale-resilient modeling of complex network spreading processes.

S2.3. Accuracy analysis of ISCG framework

The accuracy of the ISCG framework lies in its ability to faithfully preserve contagion dynamics during structural coarse-graining. To evaluate this, we analyze a toy network G consisting of six nodes: a 4-clique comprising nodes B, C, D, and E, connected to nodes A and F (Supplementary Figure 2**A**). We compute the contagion probability $P(A \to F|G)$ in the original network and compare it with the corresponding probability $P(A \to F|G_{R=4})$ in its 4-clique CGN representation (Supplementary Figure 2**B**). This comparison highlights when the ISCG framework provides an accurate approximation and reveals the potential sources of coarse-graining error.

Contagion in the original network. To calculate $P(A \to F|G)$, we consider all feasible contagion pathways through the 4-clique. These can be categorized into three distinct types: (i) *Complete clique infection pathway.*

- A infects a node in the clique (e.g., B);
- The infection spreads to all nodes in the 4-clique (B, C, D, E);
- At least one infected clique node transmits the infection to F.

The associated probability is:

$$P_{\text{complete}}(A \to F|G) = \beta\Lambda^4_{1\to3}(\beta)\left[1-(1-\beta)^2\right], \quad \text{(S12)}$$

where $\Lambda^4_{1\to3}(\beta)$ denotes the probability of full clique infection from a single seed (see Eq. S5).

(ii) *Partial clique infection pathway: single node.*

- B infects one additional node in the clique (e.g., C), without triggering full clique infection;
- The infected node directly transmits the contagion to F.

The corresponding probability is:

$$P_{\text{single}}(A \to F|G) = 2\beta^3(1-\beta)^4. \quad \text{(S13)}$$

(iii) *Partial clique infection pathway: multiple nodes.*

- B infects two other clique nodes (e.g., C and D);
- These nodes attempt to infect F, without infecting the rest of the clique.

The associated probability is:

$$P_{\text{multiple}}(A \to F|G) = 2\beta^2\Lambda^3_{1\to2}(\beta)(1-\beta)^3. \quad \text{(S14)}$$

Summing all pathways, the total contagion probability in the original network is:

$$P(A \to F|G) = 4\beta^9 - 19\beta^8 + 32\beta^7 - 19\beta^6 - 3\beta^5 + 4\beta^4 + 2\beta^3. \quad \text{(S15)}$$

Contagion in the coarse-grained network. In the 4-clique CGN $G_{R=4}$, the fully connected subgraph is abstracted into a super-node H, resulting in a simplified contagion process:

- A infects H with probability β;
- H subsequently infects F with effective probability $\beta_{\text{eff}} = \beta[1-(1-\beta)^2]$, reflecting two outgoing connections.

The total contagion probability is:

$$P(A \to F|G_{R=4}) = \beta \cdot \beta_{\text{eff}} = 2\beta^2 - \beta^3. \quad \text{(S16)}$$

This abstraction dramatically reduces computational complexity while maintaining reasonable accuracy when the coarse-graining condition $\beta \geq \hat{\beta}_4$ is satisfied. The analysis illustrates that the ISCG framework can effectively preserve contagion behavior even under structural simplification, provided that critical dynamics within cliques are properly accounted for.

Accuracy and source of error. The ISCG framework assumes that when the transmission probability β exceeds a critical threshold $\hat{\beta}_4$, the contagion process within a 4-clique becomes complete—that is, all nodes in the clique are eventually infected. Under this condition, the coarse-grained representation accurately preserves the contagion dynamics of the original network. Specifically, the probability of contagion between nodes A and F in the original network, denoted by $P(A \to F \mid G)$, becomes equivalent to the probability computed on the 4-clique CGN, $P(A \to F \mid G_{R=4})$.

To understand this behavior, we recall the decomposition:

$$P(A \to F \mid G) = P_{\text{complete}} + P_{\text{single}} + P_{\text{multiple}}. \quad \text{(S17)}$$

When $\beta \geq \hat{\beta}_4$, the terms P_{single} and P_{multiple} become negligible, as they scale with high powers of $(1-\beta)$, which decay rapidly. Quantitatively,

$$P_{\text{single}} = 2\beta^3(1-\beta)^4 \to 0,$$
$$P_{\text{multiple}} = 2\beta^2\Lambda^3_{1\to2}(\beta)(1-\beta)^3 \to 0$$

as $(1-\beta) \to 0$. Conversely, P_{complete} becomes dominant, since $\Lambda^4_{1\to3}(\beta) \to 1$ and $1-(1-\beta)^2 \to 1$. Thus, we have

$$P(A \to F \mid G) \to P_{\text{complete}} = P(A \to F \mid G_{R=4}). \quad \text{(S18)}$$

This convergence is illustrated in Supplementary Figure 2**C**, where the theoretical and simulated probabilities for both G and $G_{R=4}$ align precisely at $\beta = \hat{\beta}_4$. Below this threshold ($\beta < \hat{\beta}_4$), the CGN tends to overestimate $P(A \to F)$, as it assumes that internal clique contagion is always complete. However, in the original network, partial contagion pathways (P_{single} and P_{multiple}) dominate under low

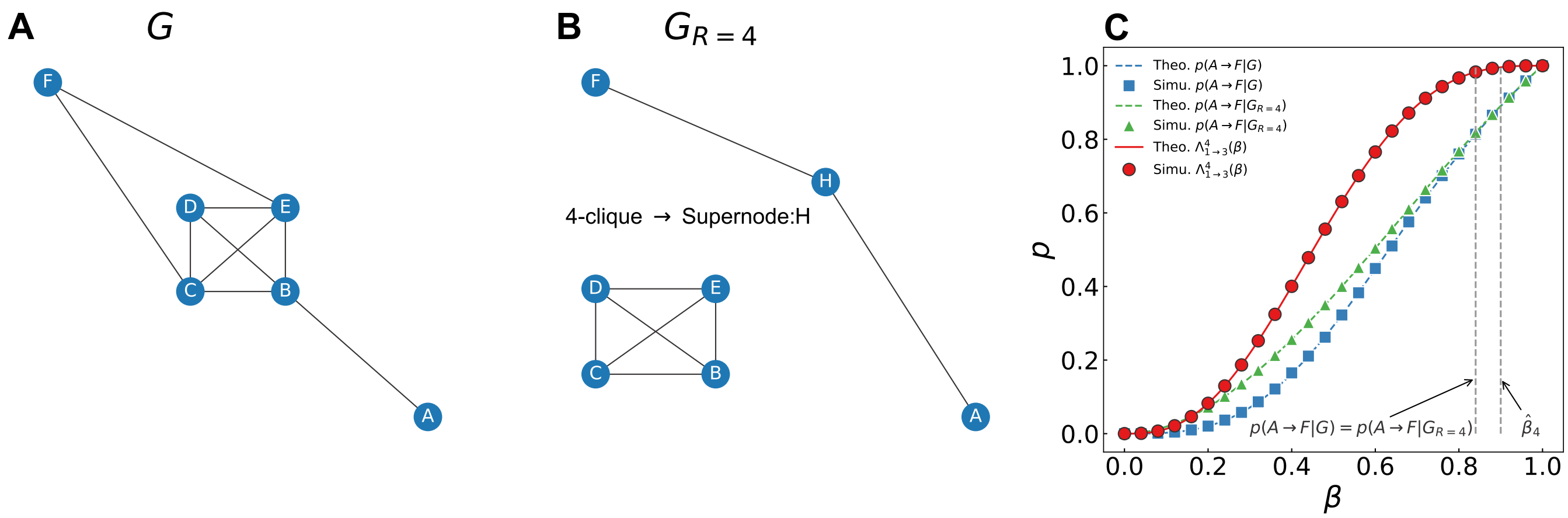

Supplementary Figure 2. **Validation of the Iterative Structural Coarse-Graining (ISCG) framework in preserving contagion dynamics.** **A**, A toy network G, composed of two isolated nodes (A and F) and a 4-clique substructure consisting of nodes B, C, D, and E. **B**, The corresponding 4-clique coarse-grained network (CGN) $G_{R=4}$, where the 4-clique is reduced to a super-node H via the ISCG process. **C**, Comparison of the infection probability $P(A \to F)$ between the original network G and its coarse-grained representation $G_{R=4}$ as a function of the transmission probability β. The figure also shows $\Lambda^4_{1\to3}(\beta)$, the theoretical probability that a single infected node leads to full contagion within a 4-clique. Simulation results (markers) exhibit strong agreement with theoretical predictions (dashed lines). Vertical dashed lines indicate key thresholds: the transmission probability $\hat{\beta}_4$ at which $\Lambda^4_{1\to3}(\beta) = 1$, and the value of β at which $P(A \to F \mid G) = P(A \to F \mid G_{R=4})$, confirming the validity of the ISCG framework in preserving contagion dynamics.

transmission probability, leading to observable discrepancies between the two representations. These discrepancies arise because, at lower β, the full-spread assumption embedded in the CGN does not hold. As β increases and approaches $\hat{\beta}_4$, the complete contagion pathway (P_{complete}) becomes increasingly dominant, and the contributions from partial pathways diminish, ensuring convergence of the full and reduced models:

$$P(A \to F \,|\, G) = P(A \to F \,|\, G_{R=4}) \quad \text{for} \quad \beta \geq \hat{\beta}_4. \tag{S19}$$

Interestingly, we also observe an empirical threshold β_{eq} at which the contagion probabilities in the full and coarse-grained networks become equal:

$$P(A \to F \,|\, G) = P(A \to F \,|\, G_{R=4}) \quad \text{at} \quad \beta = \beta_{\text{eq}}.$$

Surprisingly, $\beta_{\text{eq}} \ll \hat{\beta}_4$, indicating that the ISCG framework performs better in practice than predicted by the theoretical critical threshold. This empirical robustness highlights the practical utility and flexibility of the ISCG method, which not only achieves theoretical accuracy under ideal conditions but also maintains strong performance across a broader range of parameters, exceeding conservative estimates from analytical thresholds.

S2.4. Clique-selection strategies and computational scaling

Although the ISCG framework is conceptually formulated in terms of maximal cliques, its practical implementation does not intrinsically rely on identifying the exact largest clique at each iteration. The largest-clique–first strategy introduced in the main text serves primarily as a conceptual illustration, highlighting the self-similar nature of the iterative coarse-graining process and clarifying why contagion dynamics can be preserved across scales. More generally, the framework only requires the contraction of cliques of size at least k, and the order in which such cliques are identified and merged can be flexibly chosen. To assess the robustness and scalability of ISCG with respect to the choice of contraction strategy, we systematically compare three representative approaches illustrated in Supplementary Fig. 3: (i) *Largest-Maximal Clique Contraction (LMC)*, which prioritizes contraction of the largest available maximal cliques; (ii) *All-Maximal* $\geq k$ *Clique Contraction (AMkC)*, which contracts all maximal cliques above a fixed size threshold in parallel; and (iii) *Random Fixed-k Clique Contraction (RFkC)*, which randomly contracts fixed-size k-cliques and avoids explicit enumeration of maximal cliques.

Compression trajectories and final reduction levels. Unless otherwise stated, all results in this subsection are obtained for the GrQc collaboration network, with coarse-graining performed to k-clique coarse-grained networks. Supplementary Figures 4**A**,**E** show the evolution of node and edge compression ratios (N_r/N_0 and E_r/E_0) as functions of the coarse-graining step t for $k = 3$. The three clique-contraction strategies exhibit clearly distinct reduction trajectories, reflecting differences in how aggressively cliques are identified and merged during the iterative process. All-Maximal $\geq k$ Clique Contraction (AMkC) leads to the most rapid reduction in both nodes and edges, as contracting all eligible cliques in each iteration accelerates structural simplification. In contrast, Largest-Maximal Clique Contraction (LMC) produces a more gradual, stepwise reduction, resulting from its sequential prioritization of individual large cliques. Random Fixed-k Clique Contraction (RFkC) exhibits intermediate behavior, balancing the total number of contraction steps against the computational cost per step. Despite these pronounced differ-

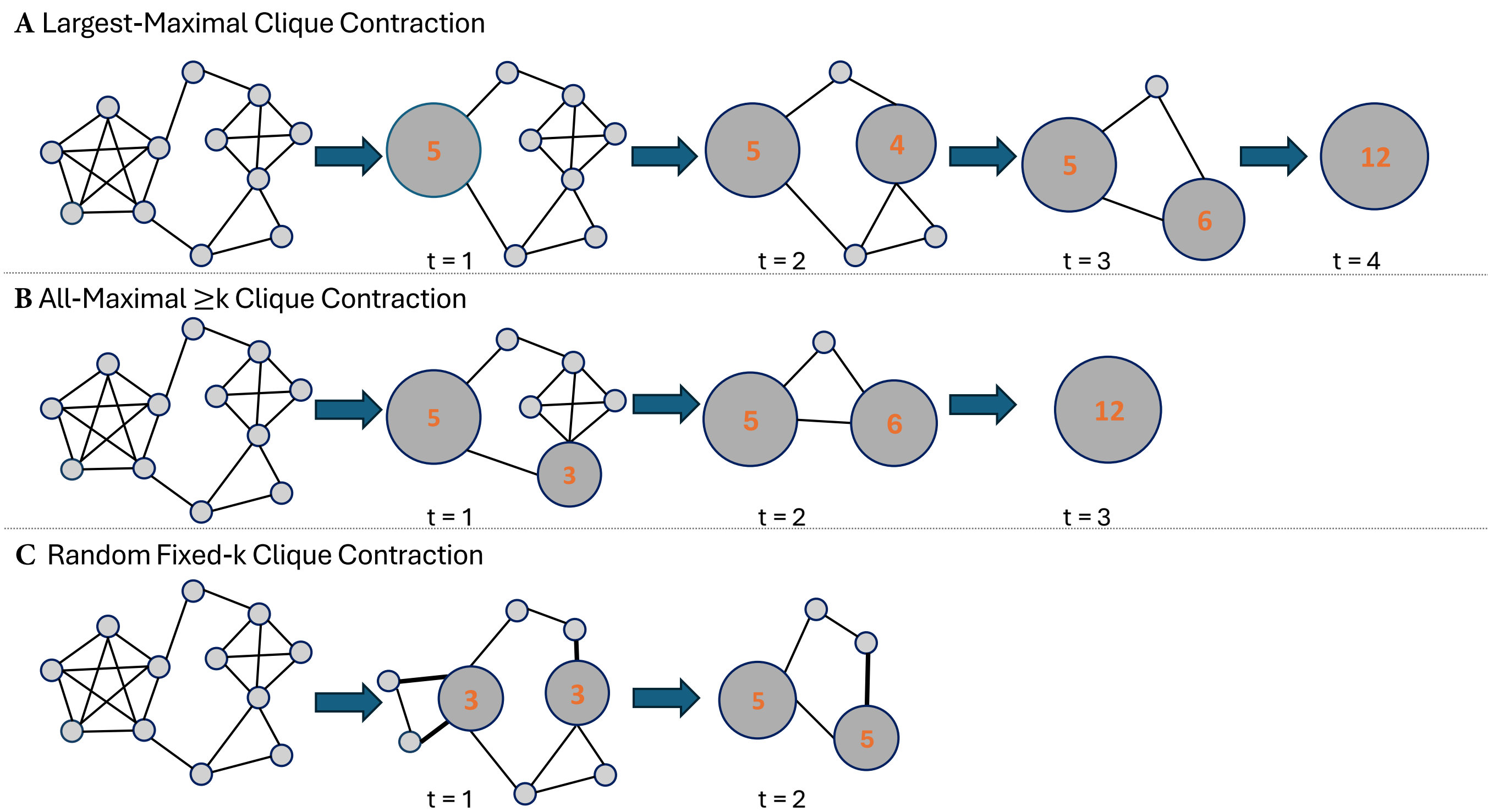

Supplementary Figure 3. **Illustration of three contraction strategies for k-clique coarse-graining ($k = 3$).** A toy network containing overlapping cliques of different sizes (including a 5-clique, a 4-clique, and multiple 3-cliques) is used to illustrate how different contraction strategies operate within the same coarse-graining framework. Numbers inside super-nodes indicate the number of original nodes represented. **A, Largest-Maximal Clique Contraction (LMC)**. This is the strategy used in the original formulation of our method. At each iteration, the algorithm identifies and contracts the current largest maximal clique in the network, proceeding in a top–down manner (5-clique → 4-clique → 3-clique). This produces a sequence of intermediate networks in which large dense subgraphs are prioritized for contraction. **B, All-Maximal $\geq k$ Clique Contraction (AMkC)**. At each step, all maximal cliques of size at least $k = 3$ are identified and contracted simultaneously. If a node belongs to multiple eligible cliques, one clique is selected at random for contraction, ensuring that each node is merged only once per step. Although this strategy follows a different contraction order and yields a different sequence of intermediate graphs than panel **A**, it converges to the same final coarse-grained structure in this example. **C, Random Fixed-k Clique Contraction (RFkC)**. This strategy contracts only k-cliques (here, 3-cliques), selected at random, without explicitly detecting larger maximal cliques. By avoiding maximal-clique enumeration, this approach yields coarse-grained networks that may differ in their detailed connectivity from those produced by strategies **A** and **B**. However, these structural differences are limited and do not affect the resulting contagion dynamics: in all cases, the coarse-grained networks satisfy the same clique-elimination constraint (i.e., no remaining k-cliques) and exhibit indistinguishable spreading behavior.

ences in compression trajectories, all three strategies converge to similar final reduction levels. As shown in panels **B,F**, the final node and edge compression ratios for k-clique coarse-grained networks with $k = 3$–6 differ only marginally across strategies. This demonstrates that, for a fixed coarse-graining scale k, the overall degree of structural reduction achieved by ISCG is largely insensitive to the specific clique-contraction strategy, even though the intermediate reduction pathways differ substantially.

Preservation of contagion dynamics. To assess dynamical fidelity, contagion outcomes on the coarse-grained networks are compared with those on the original network at $\beta = \hat{\beta}_k$. Supplementary Figure 4**C** shows the final infected fraction in the coarse-grained networks plotted against that of the original network. Across all values of k and all three contraction strategies, data points lie close to the identity line, indicating accurate preservation of the macroscopic outbreak size. The inset confirms that deviations in final outbreak size remain small and comparable across strategies. Microscopic dynamical fidelity is quantified in Supplementary Figure 4**G**, which reports the RMSE between node-level infection probability vectors together with their Pearson correlation coefficient. All three strategies yield similarly low RMSE values and near-unity correlations, demonstrating that node-level infection patterns are preserved with comparable accuracy. Together, panels **C** and **G** show that the choice of clique-contraction strategy has negligible impact on the preservation of contagion dynamics, at both macroscopic and microscopic levels.

Runtime behavior and computational cost. Supplementary Figures 4 **D,H** compare the computational costs associated with the three contraction strategies. Panel **D** reports the runtime per coarse-graining step as a function of step index t. While LMC and AMkC exhibit relatively stable per-step runtimes, RFkC displays intermittent spikes. These fluctuations arise from the batched clique-detection scheme

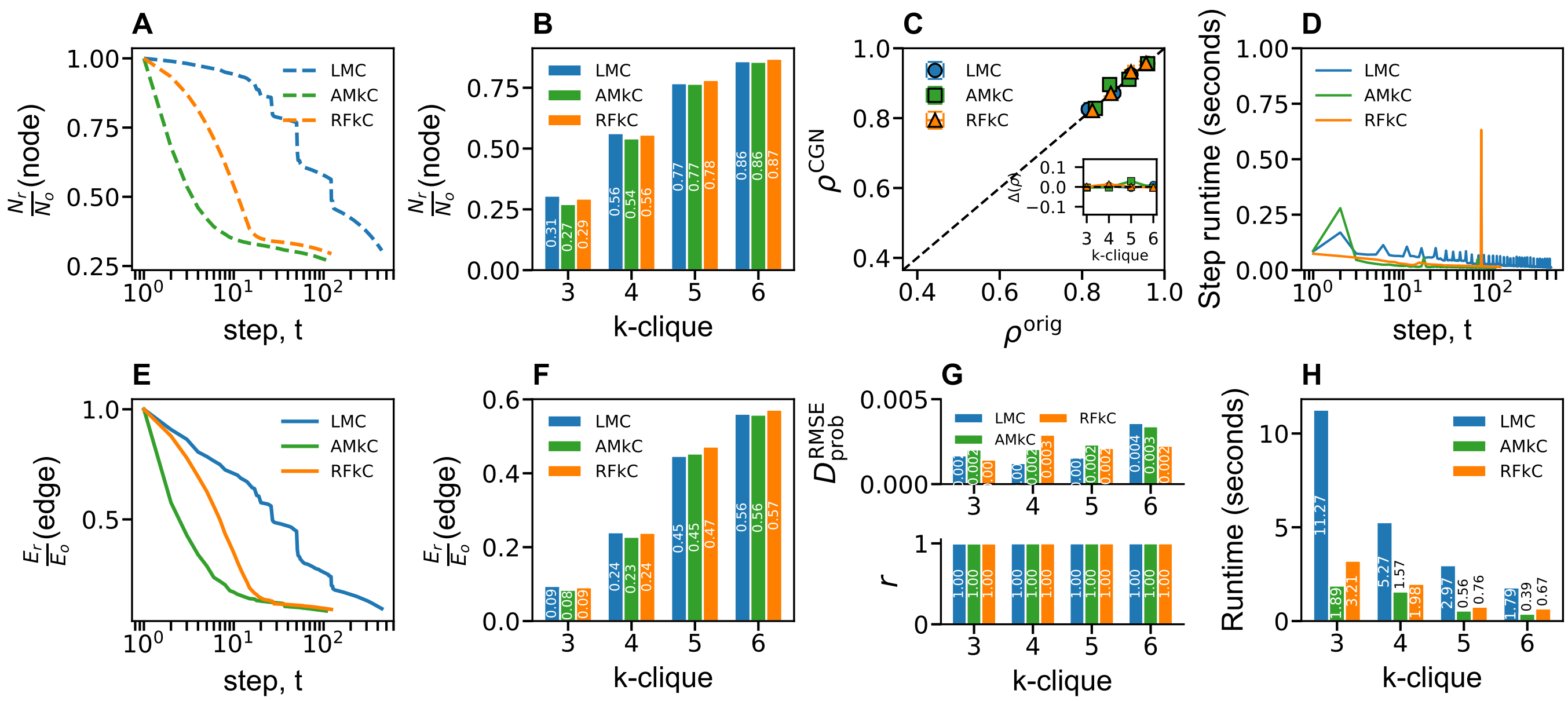

Supplementary Figure 4. **Comparison of compression efficiency, dynamical fidelity, and runtime across three clique-contraction strategies applied to the GrQc dataset.** Results are shown for the three coarse-graining strategies illustrated in Supplementary Fig. 3: Largest-Maximal Clique Contraction (LMC), All-Maximal $\geq k$ Clique Contraction (AMkC), and Random Fixed-k Clique Contraction (RFkC). **A,E**, Evolution of node and edge compression ratios (N_r/N_0 and E_r/E_0) as a function of coarse-graining step t for $k = 3$ clique coarse-grained networks, illustrating the different contraction trajectories induced by the three strategies. **B,F**, Final node and edge compression ratios for k-clique coarse-grained networks ($k = 3$ to 6) obtained using the three strategies, showing comparable levels of network size reduction across strategies. **C**, Scatter plot of the final infected fraction in the coarse-grained networks versus that of the original network at $\beta = \hat{\beta}_k$. The inset shows the deviation in final outbreak size between the coarse-grained and original networks for $k = 3$–6. **G**, Node-level dynamical fidelity quantified by the root-mean-square error (RMSE) between infection probability vectors (top) and their Pearson correlation coefficient (bottom). Panels C and G indicate that the three contraction strategies yield comparable accuracy in preserving contagion dynamics. **D**, Runtime per coarse-graining step as a function of step t. The occasional spikes observed for the RFkC strategy (orange) reflect its batched clique-detection scheme: to avoid the high cost of exhaustively identifying all small k-cliques in a single step, only a bounded number of cliques are processed per iteration. As a result, steps in which contraction induces a sudden proliferation of new k-cliques can lead to transient increases in search time. **H**, Total runtime required to construct k-clique coarse-grained networks. In contrast to the similar compression and dynamical accuracy observed in panels A–C, the three strategies exhibit substantial differences in computational cost.

used in RFkC, in which only a bounded number of fixed-k cliques are processed per iteration to avoid the high cost of exhaustive clique enumeration. When contraction induces a temporary proliferation of eligible k-cliques, additional search effort is required, leading to transient increases in runtime. Panel **H** summarizes the total runtime required to construct the k-clique coarse-grained networks. In contrast to the similar compression efficiency and dynamical fidelity observed above, the three strategies exhibit substantial quantitative differences in computational cost. This highlights that, while contraction order has little effect on the structural and dynamical outcomes of ISCG, it plays an important role in determining practical runtime performance.

Cross-dataset comparison. To examine the generality of these observations, we apply the three contraction strategies to four real-world networks: GrQc, CondMat, HepPh, and NetSci2019. Results for $k = 3, 4, 5$, and 6 are summarized in Supplementary Fig. 5. Across all datasets, the three strategies achieve comparable edge compression ratios (top row) and similarly high Pearson correlations between node-level infection probability vectors in the original and coarse-grained networks (middle row), indicating consistent preservation of contagion dynamics. In contrast, the total runtime (bottom row) varies substantially across strategies, with LMC generally incurring the highest computational cost. Together, these results show that the equivalence of compression efficiency and dynamical fidelity across contraction strategies is robust across network domains and sizes, whereas computational cost remains strongly strategy-dependent.

Empirical scaling of computational cost. Finally, we quantify the scaling behavior of the three contraction strategies with network size when coarse-graining to $k = 3$ (Supplementary Fig. 6). The number of coarse-graining steps t scales with network size as $t \sim CN^{\alpha}$ (top row). LMC and AMkC exhibit approximately linear scaling ($\alpha \approx 1.0$), whereas RFkC shows sublinear scaling in the number of steps ($\alpha \approx 0.6$), reflecting its more aggressive reduction per iteration. The mean runtime per step $\langle s \rangle$ scales as $\langle s \rangle \sim CN^{\beta}$ (middle row). LMC and AMkC display similar per-step scaling ($\beta \approx 1.1$), while RFkC incurs a higher per-step cost with a steeper exponent ($\beta \approx 1.6$), consistent with its batched clique-processing scheme. Combining these effects, the total runtime scales as $S \sim CN^{\gamma}$ (bottom row). All three strategies exhibit polynomial scaling with network size, with $\gamma \approx 2.0$ for AMkC,

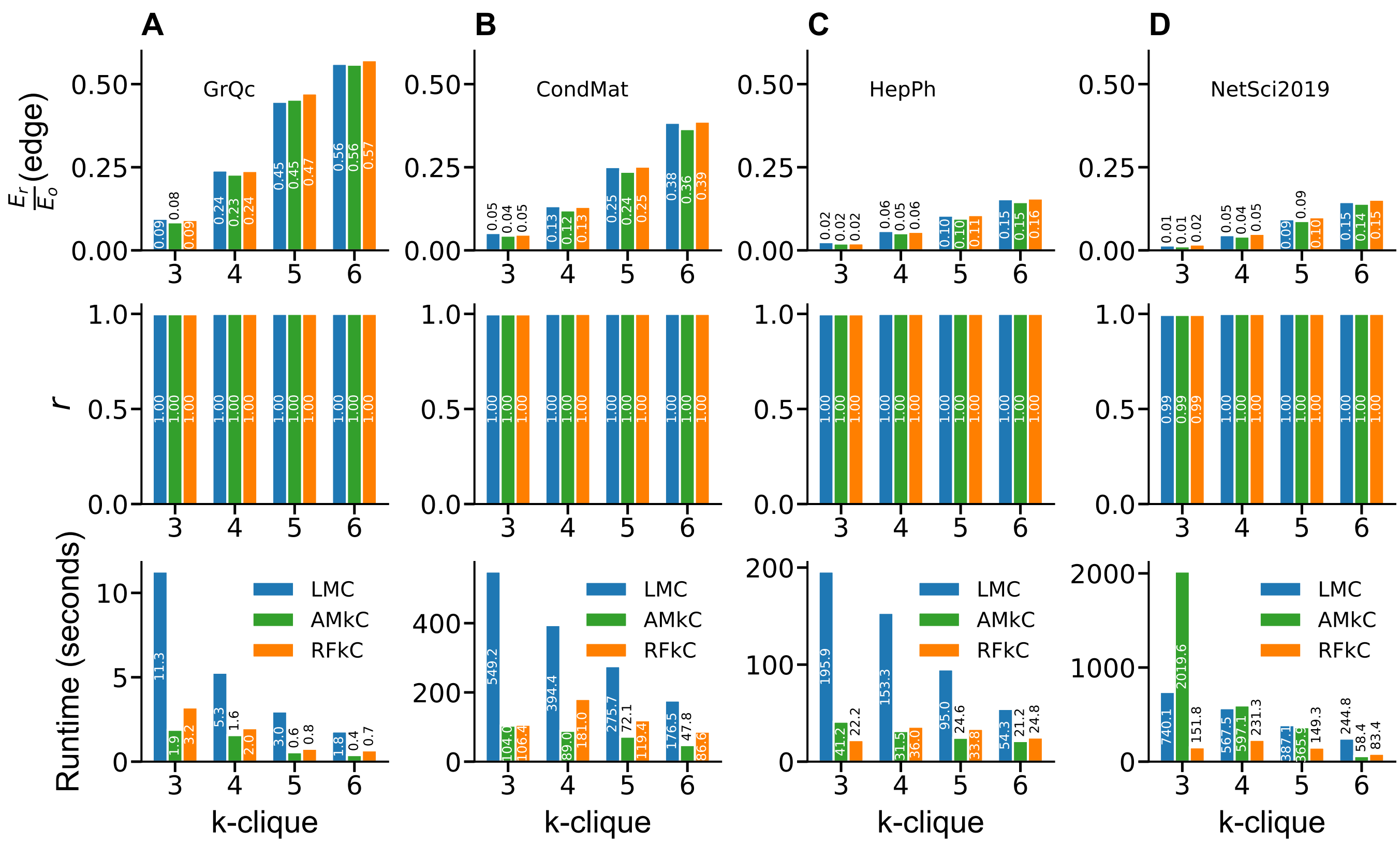

Supplementary Figure 5. **Cross-dataset comparison of compression efficiency, dynamical fidelity, and runtime for different clique-contraction strategies.** The three contraction strategies introduced in Supplementary Fig. 4—Largest-Maximal Clique Contraction (LMC), All-Maximal $\geq k$ Clique Contraction (AMkC), and Random Fixed-k Clique Contraction (RFkC)—are applied to four real-world networks: GrQc (**A**), CondMat (**B**), HepPh (**C**), and NetSci2019 (**D**), to construct k-clique coarse-grained networks with $k = 3, 4, 5,$ and 6. Top row: edge compression ratio E_r/E_0 achieved by the three strategies for different values of k. Middle row: Pearson correlation coefficient r between node-level infection probability vectors in the original and coarse-grained networks, quantifying the preservation of contagion dynamics. Bottom row: total runtime required to construct the corresponding k-clique coarse-grained networks (in seconds). Across all datasets and values of k, the three strategies yield comparable levels of network compression and dynamical fidelity, while exhibiting pronounced differences in computational cost.

$\gamma \approx 2.1$ for LMC, and $\gamma \approx 2.2$ for RFkC. Importantly, no exponential growth of runtime with N is observed for any strategy, confirming the practical scalability of the ISCG framework despite its formulation in terms of clique-based operations.

S3. SUPPLEMENTARY RESULTS

S3.1. Transmission-dependent structural compression

The exact-preservation framework described in the main text generalizes across a broad range of empirical networks. To quantify the amount of admissible structural reduction, we examine how the maximum compression permitted by the theoretically predicted matching condition varies with the transmission probability β for networks spanning biological, social, technological, communication, and transportation systems (Supplementary Fig. 7). Supplementary Fig. 7**A**,**B** show the retained node and edge fractions (N_r/N_0 and E_r/E_0) corresponding to the smallest clique size satisfying the matching condition at each transmission probability. Across all networks, increasing transmission probability permits progressively stronger structural compression, because smaller cliques become dynamically saturated and can therefore be safely contracted while maintaining exact preservation. Although the achievable compression varies quantitatively across datasets, reflecting differences in mesoscale organization, the same qualitative trend is consistently observed. Representative networks exhibiting strong, intermediate, and weak compression are highlighted, while the remaining datasets are shown in gray. Supplementary Fig. 7**C** further compares node and edge compression directly. Across all empirical networks, edge reduction consistently exceeds node reduction, indicating that ISCG preferentially removes structurally redundant intra-clique connectivity while retaining the backbone most relevant to contagion spreading. These results demonstrate that the amount of admissible compression is jointly determined by the transmission regime and the mesoscale organization of the underlying network. Rather

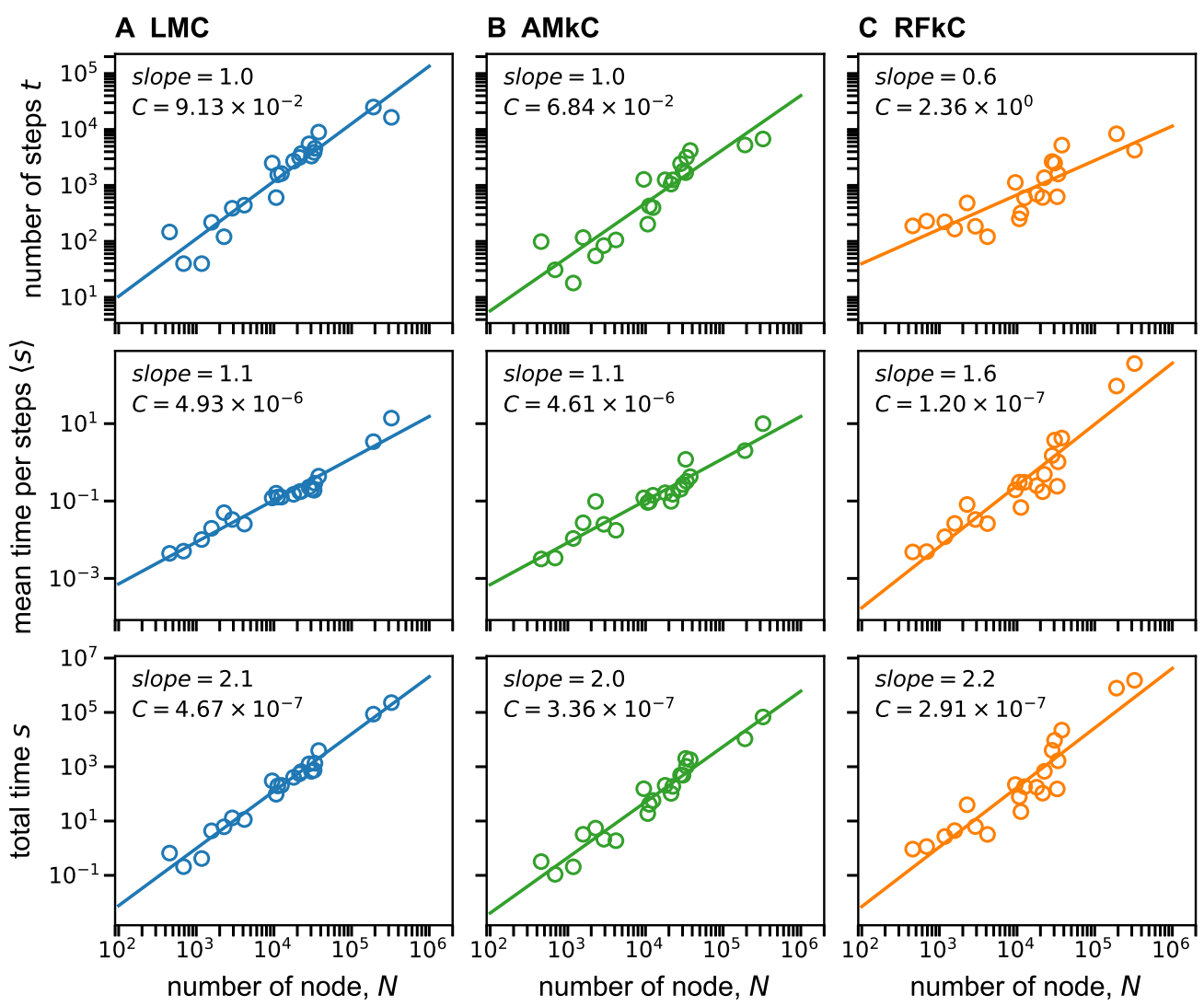

Supplementary Figure 6. **Empirical scaling of computational cost for different clique-contraction strategies.** Shown are the scaling behaviors of three contraction strategies—Largest-Maximal Clique Contraction (LMC, **A**), All-Maximal $\geq k$ Clique Contraction (AMkC, **B**), and Random Fixed-k Clique Contraction (RFkC, **C**)—when coarse-graining networks to $k = 3$ clique coarse-grained networks. **Top row:** number of coarse-graining steps t as a function of network size N, fitted as $t \sim CN^{\alpha}$. LMC and AMkC exhibit approximately linear scaling ($\alpha \approx 1.0$), while RFkC shows sublinear scaling in the number of steps ($\alpha \approx 0.6$). **Middle row:** mean runtime per coarse-graining step $\langle s \rangle$ as a function of N, fitted as $\langle s \rangle \sim CN^{\beta}$. Both LMC and AMkC display similar scaling ($\beta \approx 1.1$), whereas RFkC incurs a substantially higher per-step cost with a steeper scaling exponent ($\beta \approx 1.6$). **Bottom row:** total runtime S as a function of N, fitted as $S \sim CN^{\gamma}$. All three strategies exhibit polynomial scaling with network size, with $\gamma \approx 2.0$ for AMkC, $\gamma \approx 2.1$ for LMC, and $\gamma \approx 2.2$ for RFkC. Despite quantitative differences in scaling exponents and prefactors, no exponential growth of runtime with N is observed for any strategy.

than producing a single coarse-grained representation, the matching condition defines a continuum of exact-preservation scales, enabling the level of structural reduction to adapt naturally to both the spreading dynamics and the network architecture.

S3.2. Structural basis of dynamical fidelity

The high dynamical fidelity observed throughout the main text suggests that clique-based coarse-graining preserves structural characteristics that are most relevant to contagion spreading. To identify these characteristics, we systematically examine how network organization evolves across coarse-graining scales. Rather than assessing reconstruction accuracy, the objective is to determine which structural features remain invariant or approximately self-similar under iterative clique contraction and may therefore provide the structural basis for the observed preservation of contagion dynamics.

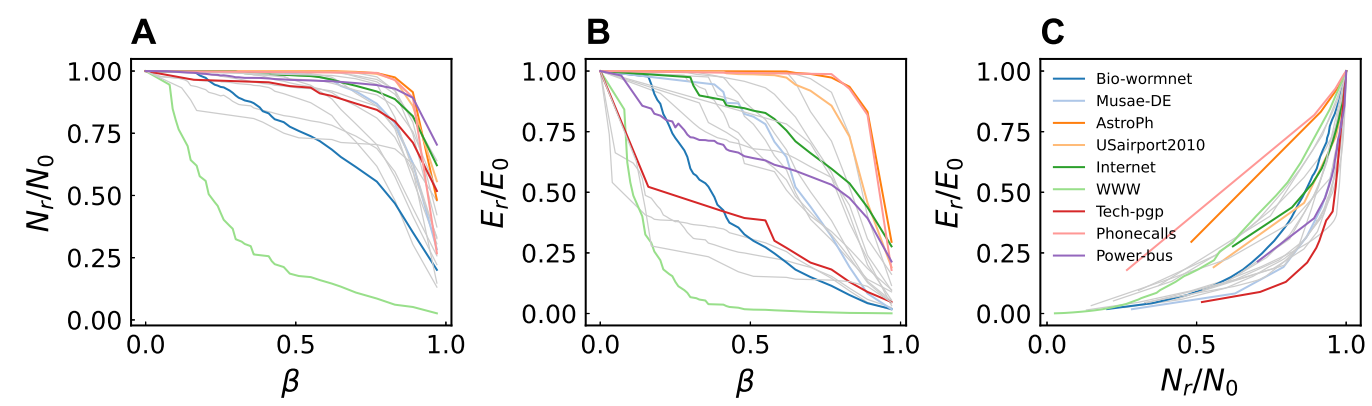

Supplementary Figure 7. **Transmission-dependent structural compression across real-world networks.** Structural compression permitted by the theoretically predicted matching condition as a function of the transmission probability across representative empirical networks. (**A,B**) Retained node fraction (N_r/N_0) and retained edge fraction (E_r/E_0) corresponding to the smallest clique size satisfying the matching condition at each transmission probability. Colored curves highlight representative networks exhibiting strong, intermediate, and weak compression, while gray curves denote the remaining datasets. (**C**) Relationship between retained edge and node fractions as the transmission probability varies. Across all networks, increasing transmission probability permits progressively stronger structural compression because smaller cliques satisfy the matching condition. Although the achievable compression depends quantitatively on network structure, the same qualitative behavior is consistently observed, with edge reduction exceeding node reduction throughout.

Supplementary Fig. 8 reveals two complementary aspects of the structural evolution. Panels **A-D** show the evolution of global network averages, including the mean degree $\langle d_k \rangle$, mean node strength $\langle w_k \rangle$, mean shortest-path length $\langle l_k \rangle$, and mean clustering coefficient $\langle C_k \rangle$, as functions of the retained node fraction. All four quantities vary systematically as coarse-graining proceeds, reflecting the expected changes in network size and density. These global shifts, however, do not by themselves determine whether the essential structural organization of the network is preserved.

A different picture emerges from the corresponding distributional analysis. Panels **E-H** show the complementary cumulative distributions of node degree, node strength, shortest-path length, and clustering coefficient at different clique scales. Although the characteristic scales of these quantities change under compression, their overall distributional forms remain remarkably similar. After normalizing node degree, node strength, and shortest-path length by their corresponding coarse-grained averages, the distributions collapse onto nearly universal curves across coarse-graining scales (panels **I-K**). Among these quantities, the collapse is strongest for shortest-path length, indicating that the effective distance structure of the network remains highly self-similar despite substantial structural reduction. The clustering statistics exhibit a similar degree of robustness. Although the relative abundance of nodes with extreme clustering coefficients ($c = 0$ or $c = 1$) changes as dense cliques are merged or removed, the distribution over intermediate clustering values remains largely unchanged (panel **L**). This suggests that the local mixing environment experienced by most nodes is largely preserved throughout the coarse-graining process.

Taken together, these results show that clique-based coarse-graining preserves the multiscale organization of the network rather than its exact microscopic structure. In particular, the

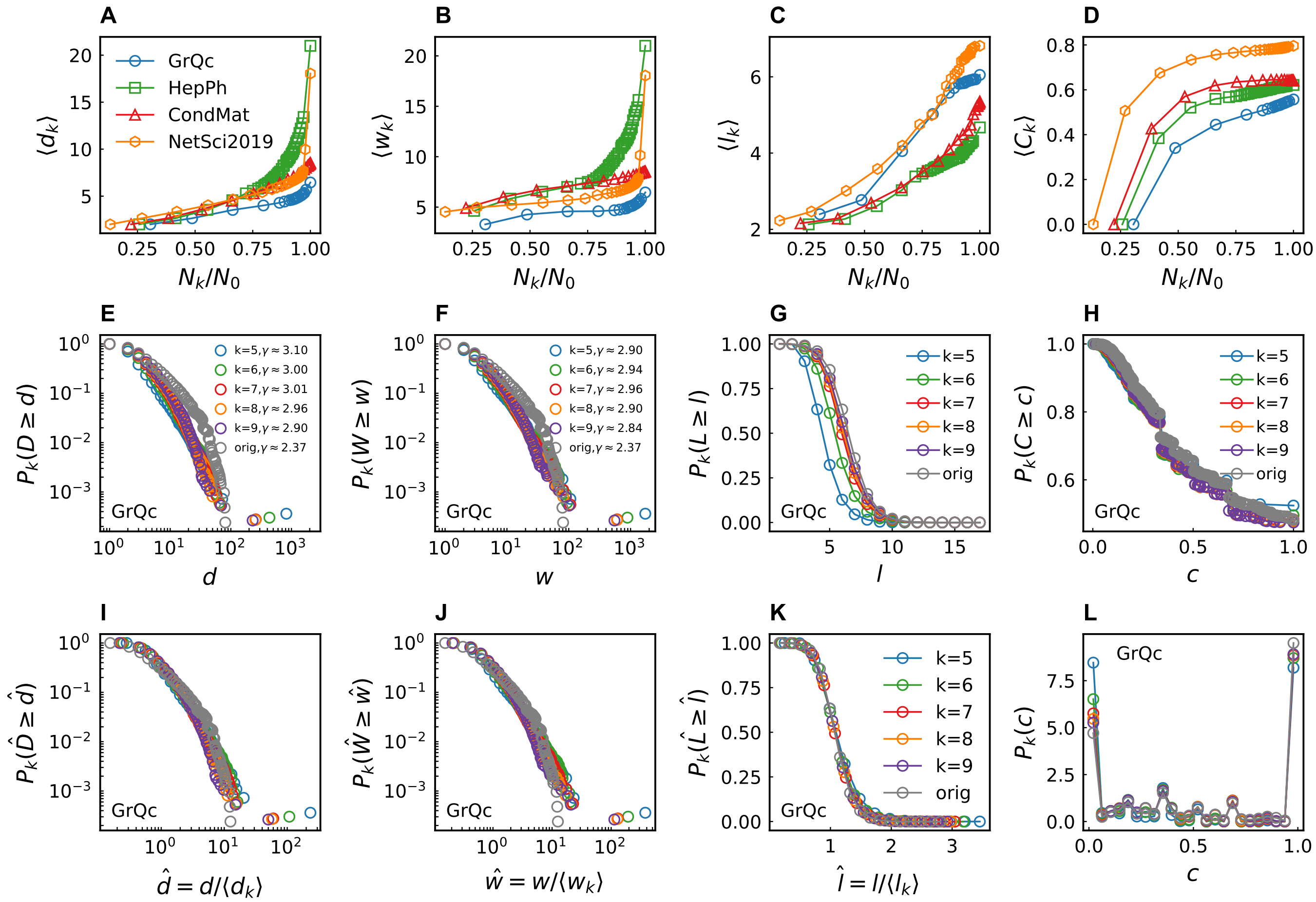

Supplementary Figure 8. **Structural properties under clique-based coarse-graining. A–D** Evolution of global structural measures as a function of the compression ratio N_k/N_0 for GrQc networks: average degree $\langle d_k \rangle$, average node strength $\langle w_k \rangle$, average shortest-path length $\langle l_k \rangle$, and average clustering coefficient $\langle C_k \rangle$. As expected, these global metrics vary systematically with the degree of compression, reflecting changes in overall network scale. **E–H** Complementary cumulative distributions of node degree d_k, node strength w_k, shortest-path length l, and clustering coefficient c for coarse-grained networks at different clique scales k, compared with the original network. **I–K** Cumulative distributions of rescaled structural quantities $\hat{d} = d/\langle d_k \rangle$, $\hat{w} = w/\langle w_k \rangle$, and $\hat{l} = l/\langle l_k \rangle$. After rescaling by the corresponding mean values, distributions at different coarse-graining scales collapse onto a common curve, most prominently for shortest-path lengths, indicating approximate self-similarity across scales. **L** Probability density of the clustering coefficient across different coarse-graining levels. While the weights of nodes with $c = 0$ or $c = 1$ change with compression, the distribution for intermediate clustering values remains largely unchanged, indicating that the coarse-graining preserves the local clustering structure of nodes not belonging to k-cliques.

approximate self-similarity of shortest-path and local clustering statistics provides a structural explanation for the high dynamical fidelity observed throughout the main text. Because epidemic spreading is governed primarily by effective transmission distances and local mixing, preserving these structural characteristics naturally leads to accurate reproduction of epidemic outcomes and spatiotemporal contagion dynamics across coarse-graining scales.

S3.3. Approximate reduction

We extend the ISCG framework by relaxing the strict structural constraint of k-cliques and adopting k-plexes as the basis for coarse-graining. Unlike k-cliques, which require complete connectivity among all nodes in a subgraph, k-plexes allow each node to miss up to k links within the group. This relaxation offers greater structural flexibility, reducing computational overhead and improving the scalability of the coarse-graining process for large networks. To balance computational efficiency with the preservation of contagion dynamics, we adopt an adaptive strategy for setting the value of k in the k-plex structure. Specifically, we define $k = 0.5 \cdot m$, where m is the maximum clique size detected in the current network at each reduction step. This ensures that the k-plexes approximate clique structures closely during early reduction stages while remaining tractable for large-scale networks.

Supplementary Figure 9 compares the performance of the approximate ISCG method with the original ISCG framework on the GrQc network. As shown in Supplementary Figure 9**A**, both methods follow similar reduction trajectories, with the k-

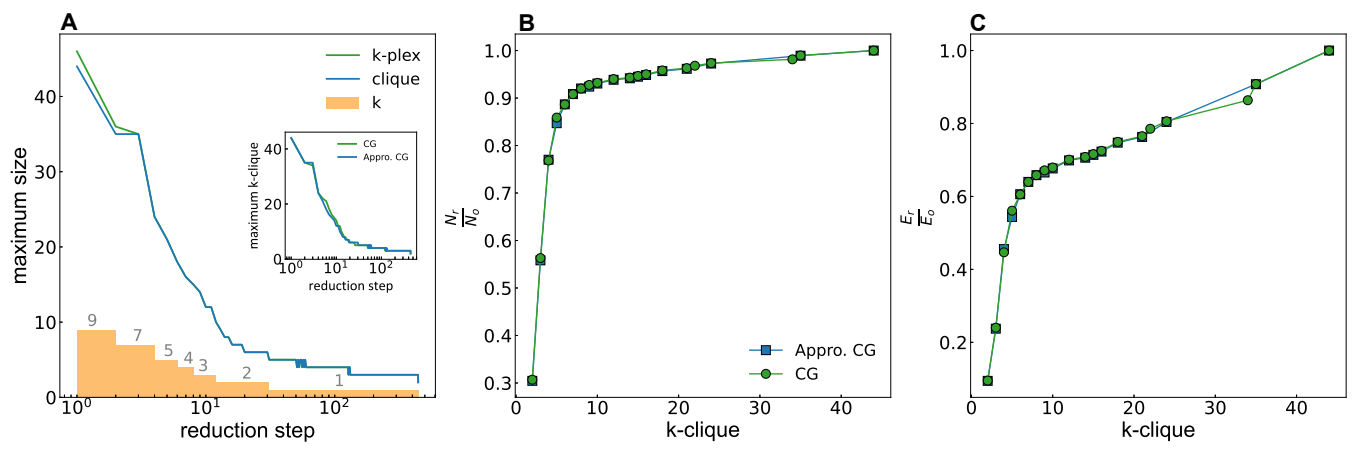

Supplementary Figure 9. **Comparison of Iterative Structural Coarse-Graining (ISCG) and approximate ISCG methods for network reduction.** **A**, Evolution of the maximal clique size (ISCG) and maximal k-plex size (approximate ISCG) at each iteration of the coarse-graining process. Orange bars indicate the k-plex values used in the approximate ISCG method, while solid curves represent the corresponding reduction trajectories for both methods. **B**, Fraction of nodes retained in the reduced networks (N_r/N_o) as a function of k. **C**, Fraction of edges retained (E_r/E_o) under both ISCG and approximate ISCG. Results for the GrQc network show that the approximate ISCG method—based on relaxed k-plex structures instead of strict k-cliques—achieves comparable reduction efficiency to the standard ISCG framework, while offering greater flexibility in structural constraints.

plex-based approach identifying larger reducible subgraphs in early iterations. The adaptively chosen k-values, shown as orange bars, highlight how the method transitions from broader to stricter structural patterns. In the later stages, the subgraphs selected by the approximate method converge to structures increasingly similar to strict k-cliques (e.g., 1-plexes), maintaining consistency with contagion dynamics. Supplementary Figures 9**B** and **C** report the retained proportions of nodes (N_r/N_o) and edges (E_r/E_o) throughout the reduction process. The approximate ISCG method achieves comparable reduction performance to the original framework, with only minor deviations observed across all reduction stages. While reduction effectiveness varies depending on the network's structural characteristics, the approximate method consistently delivers robust simplification with minimal loss of fidelity.

By leveraging the structural flexibility of k-plexes, the approximate ISCG framework extends the applicability of structural coarse-graining to networks with diverse topologies. Its adaptive nature enables efficient simplification without compromising dynamic preservation, making it a practical and scalable alternative for large-scale network reduction.

S3.4. Dynamical fidelity under coarse-graining

To complement the dynamical validation presented in the main text, we further evaluate the ISCG framework across additional empirical networks, coarse-graining scales, and temporal-alignment conditions. These analyses examine four complementary aspects of the framework: the structural origin of the global temporal shift, the robustness of spatiotemporal contagion preservation, the generality of epidemic-outcome preservation, and the robustness of the compression–accuracy trade-off. Unless otherwise stated, all results are obtained from stochastic SIR simulations on the original net-

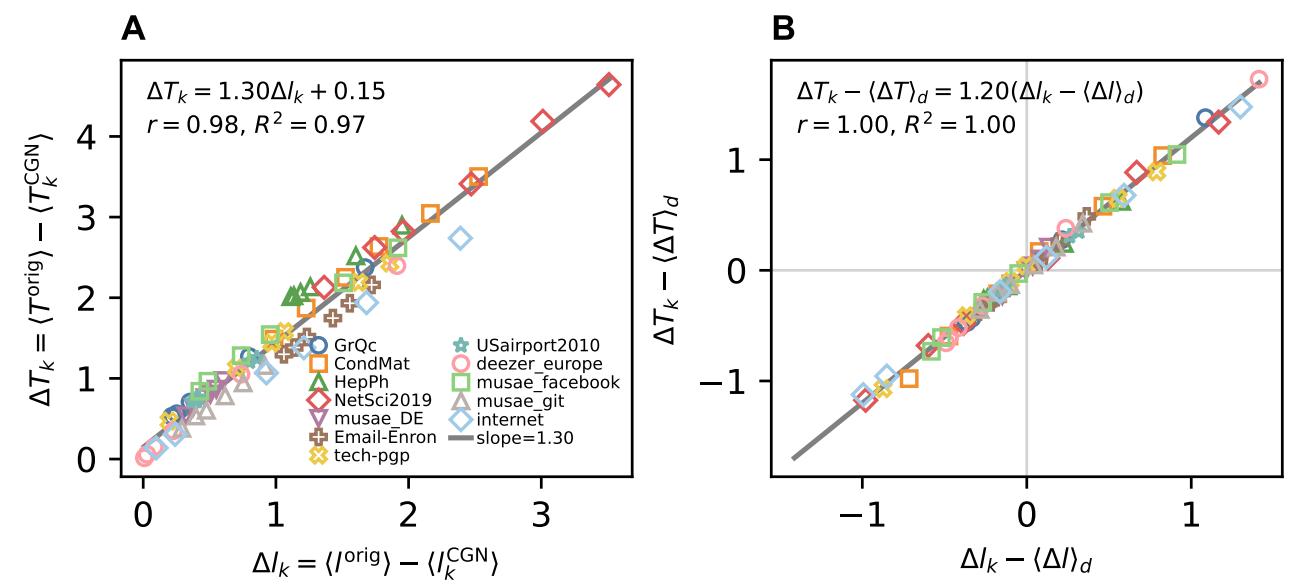

Supplementary Figure 10. **Structural origin of the global temporal shift induced by coarse-grained networks (CGNs).** Relationship between the temporal shift introduced by coarse-graining and the corresponding reduction in average shortest-path length. Each point represents one empirical network at a given k-clique coarse-graining scale ($k = 5, \ldots, 10$). **A**, Absolute temporal shift, $\Delta T_k = \langle T^{\mathrm{orig}} \rangle - \langle T_k^{\mathrm{CGN}} \rangle$, as a function of the reduction in average shortest-path length, $\Delta l_k = \langle l^{\mathrm{orig}} \rangle - \langle l_k^{\mathrm{CGN}} \rangle$, evaluated at the theoretically predicted matching rate $\beta = \hat{\beta}_k$. Across all networks and coarse-graining scales, the temporal shift is accurately described by a linear relation, $\Delta T_k = 1.30\Delta l_k + 0.15$ ($r = 0.98$, $R^2 = 0.97$), indicating that the advancement of contagion dynamics is primarily determined by path-length compression. **B**, Dataset-centered representation obtained by subtracting the mean values of Δl_k and ΔT_k for each network. Removing dataset-specific offsets collapses all measurements onto a common linear relation, $\Delta T_k - \langle \Delta T \rangle_d = 1.20(\Delta l_k - \langle \Delta l \rangle_d)$ ($r = 1.00$, $R^2 = 1.00$), demonstrating that the scale-dependent component of the temporal shift is governed by the reduction in shortest-path length. These results identify path-length compression as the dominant structural origin of the global temporal shift and provide a simple structural predictor for temporal alignment without requiring simulations on the original network.

works and their corresponding k-clique coarse-grained networks (CGNs).

Structural origin of the global temporal shift. The temporal alignment introduced in the main text raises a natural question: what determines the magnitude of the global temporal shift induced by coarse-graining? Supplementary Figure 10 shows that this shift has a simple structural origin. Across empirical networks and coarse-graining scales, the reduction in mean infection time is strongly correlated with the corresponding reduction in average shortest-path length. At the theoretically predicted matching rate, the temporal shift ΔT_k is accurately described by a linear function of the path-length reduction Δl_k, indicating that contracting densely connected subgraphs systematically shortens the effective transmission paths available for contagion.

To separate this scale-dependent effect from network-specific structural differences, we center both quantities by subtracting their dataset averages. After this normalization, measurements from all empirical networks collapse onto a common linear relation, demonstrating that the variation of the temporal shift across coarse-graining scales is governed predominantly by path-length compression. These observations provide a structural explanation for the global temporal shift introduced throughout the main text. More importantly,

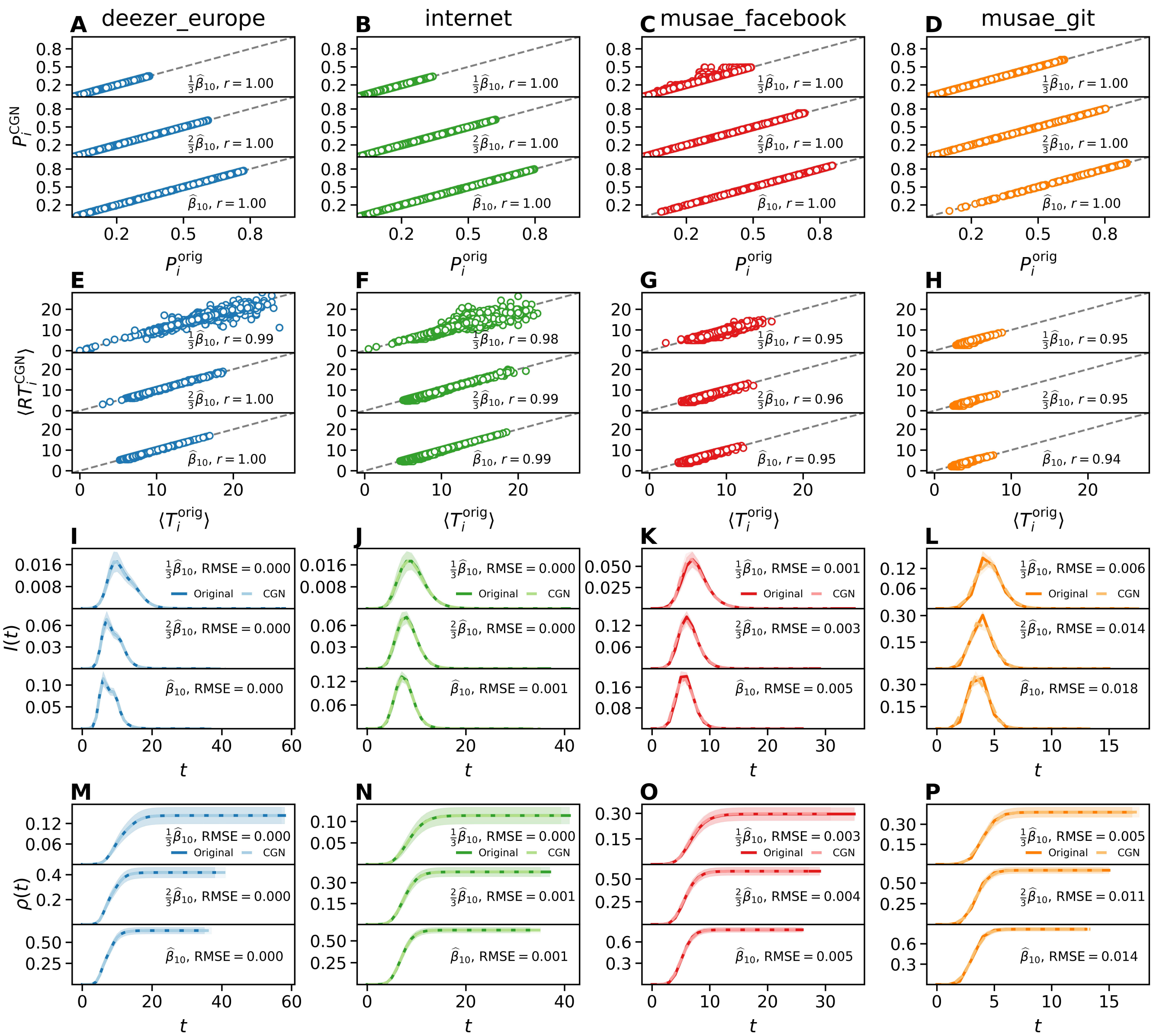

Supplementary Figure 11. **Preservation of spatiotemporal contagion dynamics in additional non-collaboration networks.** Results compare the original networks with their corresponding $k = 10$ clique coarse-grained networks (CGNs): (**A,E,I,M**) deezer_europe, (**B,F,J,N**) internet, (**C,G,K,O**) musae_facebook, and (**D,H,L,P**) musae_git. Unlike the collaboration networks analyzed in the main text, these networks are not derived from bipartite projections and therefore provide an independent test of the coarse-graining framework. For each network, results are shown for three transmission rates: $\frac{\hat{\beta}_{10}}{3} = 0.193$, $\frac{2\hat{\beta}_{10}}{3} = 0.387$, and $\hat{\beta}_{10} = 0.58$. Rows from top to bottom show node-level infection probabilities P_i^{orig} versus P_i^{CGN}, mean infection times after applying the global temporal shift ΔT, transient infected fractions $I(t)$, and cumulative outbreak trajectories $\rho(t)$. The CGN trajectories are shifted according to $t \to t + \Delta T$ before comparison. Panels **A-P** were computed from 100 randomly selected outbreak seeds, each averaged over 500 independent epidemic realizations; shaded regions in panel **I-P** denote the standard error across outbreak seeds. Across all datasets and transmission regimes, the coarse-grained networks accurately reproduce both the spatial distribution of infection risk and the temporal evolution of contagion, providing additional evidence that spatiotemporal contagion preservation extends beyond projection-derived collaboration networks.

this structural relation provides a practical means of estimating the temporal shift directly from the coarse-grained network, substantially reducing the need for simulations on the original system.

Robustness of spatiotemporal contagion preservation. The preservation of the spatiotemporal organization of contagion demonstrated in the main text is robust across both network classes and coarse-graining scales. To

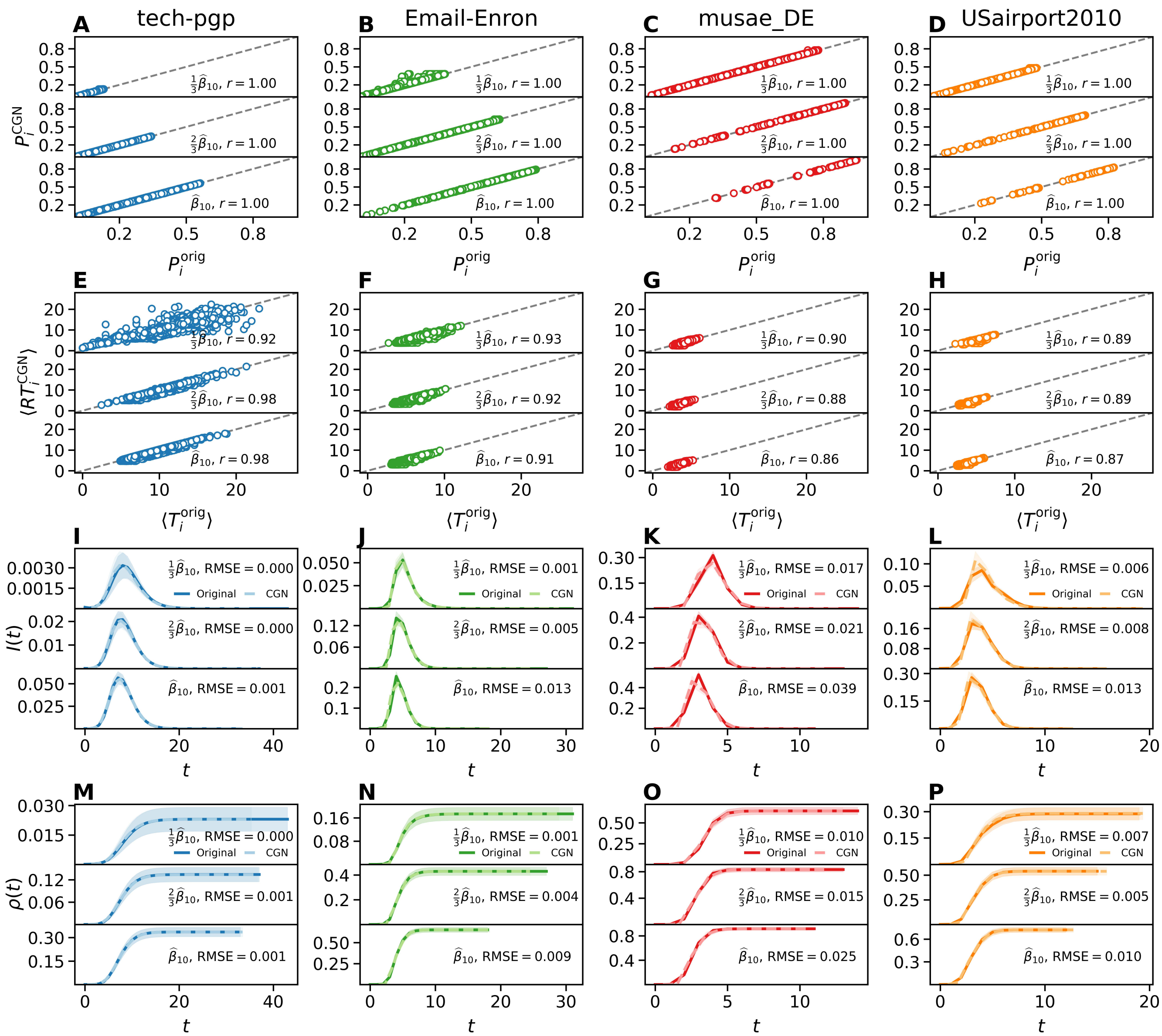

Supplementary Figure 12. **Preservation of spatiotemporal contagion dynamics in additional non-collaboration networks.** Results compare the original networks with their corresponding $k = 10$ clique coarse-grained networks (CGNs) for a second set of representative non-collaboration networks: (**A,E,I,M**) tech-PGP, (**B,F,J,N**) Email-Enron, (**C,G,K,O**) musae_DE, and (**D,H,L,P**) USairport2010. For each network, results are shown at three transmission rates: $\frac{\hat{\beta}10}{3} = 0.193$, $\frac{2\hat{\beta}10}{3} = 0.387$, and $\hat{\beta}_{10} = 0.58$. Rows from top to bottom show node-level infection probabilities, mean infection times after applying the global temporal shift ΔT, transient infected fractions $I(t)$, and cumulative outbreak trajectories $\rho(t)$. Panels **A-P** were computed from 100 randomly selected outbreak seeds, each averaged over 500 independent epidemic realizations; shaded regions in panel **I-P** denote the standard error across outbreak seeds. Consistently high agreement is observed across all datasets and transmission regimes, further demonstrating that the preservation of spatiotemporal contagion dynamics by Iterative Structural Coarse-Graining (ISCG) is robust across diverse empirical network classes.

evaluate its generality, we examine eight additional empirical networks spanning communication, technological, transportation, and online social systems: deezer_europe, internet, musae_facebook, musae_git, tech-PGP, Email-Enron, musae-DE, and USairport2010 (Supplementary Figs. 11 and 12). Across all eight datasets, node-level infection probabilities remain in near-perfect agreement ($r \approx 1$) with those of the original networks, mean infection times remain highly correlated ($r = 0.85$–1.00) after applying the global temporal shift introduced in the main text, and both transient infected fractions $I(t)$ and cumulative outbreak trajectories $\rho(t)$ exhibit consistently small RMSE values (RMSE $= 0.00$–0.04) after

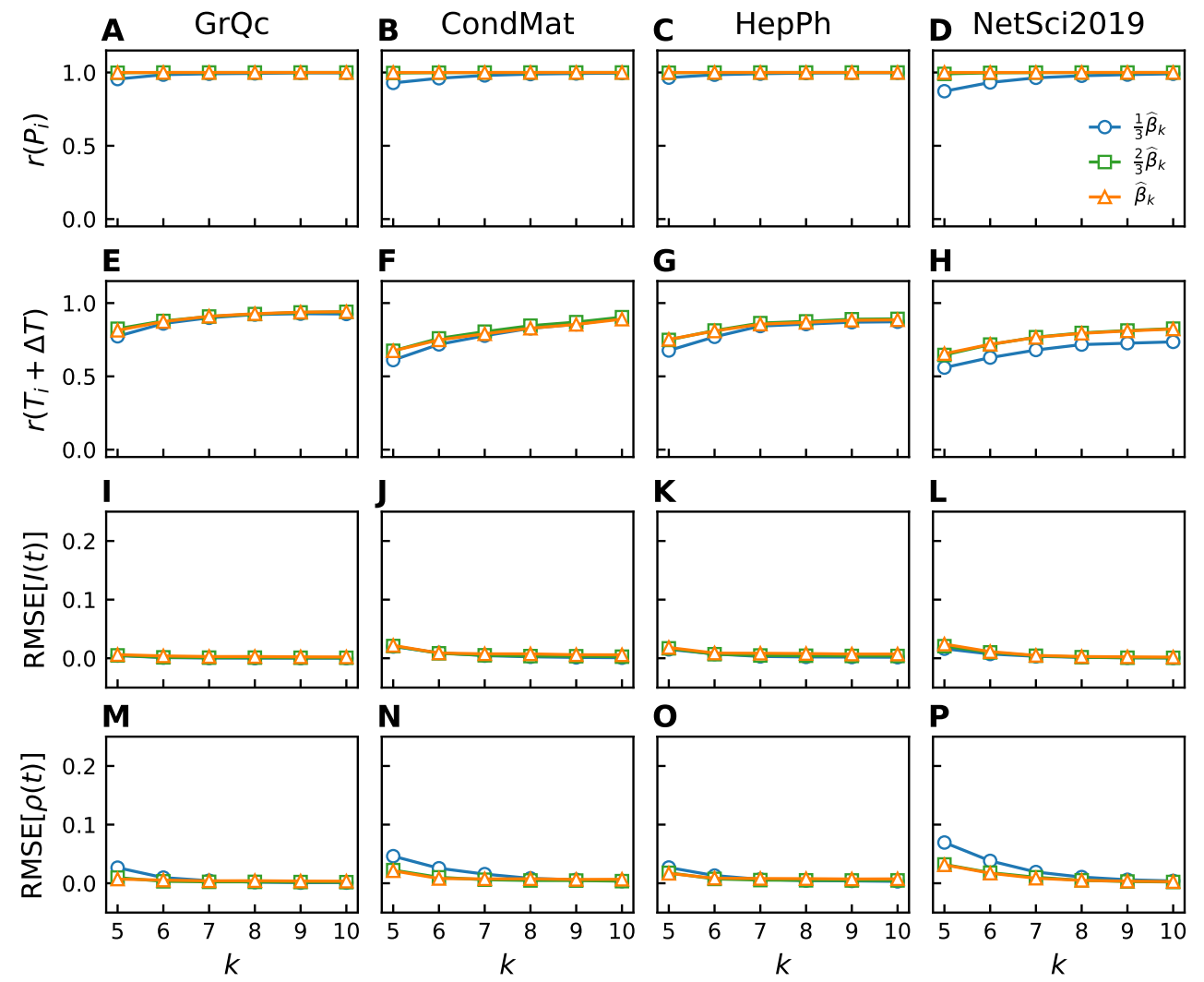

Supplementary Figure 13. **Preservation of spatiotemporal contagion dynamics across coarse-graining scales.** Temporal-dynamics accuracy of k-clique coarse-grained networks (CGNs) as the coarse-graining scale varies from $k = 5$ to 10 for four representative collaboration networks: (**A,E,I,M**) GrQc, (**B,F,J,N**) CondMat, (**C,G,K,O**) HepPh, and (**D,H,L,P**) NetSci2019. Each curve corresponds to one of three transmission regimes, $\hat{\beta}_k/3$, $2\hat{\beta}_k/3$, or $\hat{\beta}_k$, with $\hat{\beta}_k = (0.83, 0.76, 0.71, 0.66, 0.61, 0.58)$ for $k = 5, \ldots, 10$. (**A**–**D**) Pearson correlation between node-level infection probabilities P_i^{orig} and P_i^{CGN}. (**E**–**H**) Pearson correlation between mean infection times T_i^{orig} and the temporally aligned coarse-grained values $T_i^{\text{CGN}} + \Delta T$, where $\Delta T = \langle T^{\text{orig}} \rangle - \langle T^{\text{CGN}} \rangle$. (**I**–**L**) Root-mean-square error (RMSE) between the transient infected fractions $I^{\text{orig}}(t)$ and $I^{\text{CGN}}(t + \Delta T)$. (**M**–**P**) RMSE between the cumulative outbreak trajectories $\rho^{\text{orig}}(t)$ and $\rho^{\text{CGN}}(t + \Delta T)$. Across all four observables, high agreement is maintained over the entire range of coarse-graining scales. The theoretically predicted matching rate $\hat{\beta}_k$ consistently yields the highest fidelity, while comparably accurate agreement persists across a broad range of transmission regimes and clique thresholds, demonstrating that the preservation of spatiotemporal contagion dynamics is robust to the choice of coarse-graining scale.

temporal alignment. These observations demonstrate that the preservation of the spatiotemporal organization of contagion is not limited to collaboration networks but extends across diverse empirical network classes.

The same robustness is observed across coarse-graining scales. Supplementary Figure 13 summarizes the corresponding accuracy metrics as the clique threshold varies from $k = 5$ to 10. Across all coarse-graining scales, node-level infection probabilities remain nearly identical to those of the original networks, mean infection times remain highly correlated after temporal alignment, and both transient and cumulative epidemic trajectories remain accurately reproduced. Although the highest fidelity is consistently achieved at the theoretically predicted matching rate $\hat{\beta}_k$, comparable agreement is maintained over a broad range of transmission probabilities and coarse-graining scales. Taken together, Supplementary Figures 11-13 provide additional evidence that the preservation of the spatiotemporal organization of contagion by ISCG is robust across both network classes and coarse-graining scales.

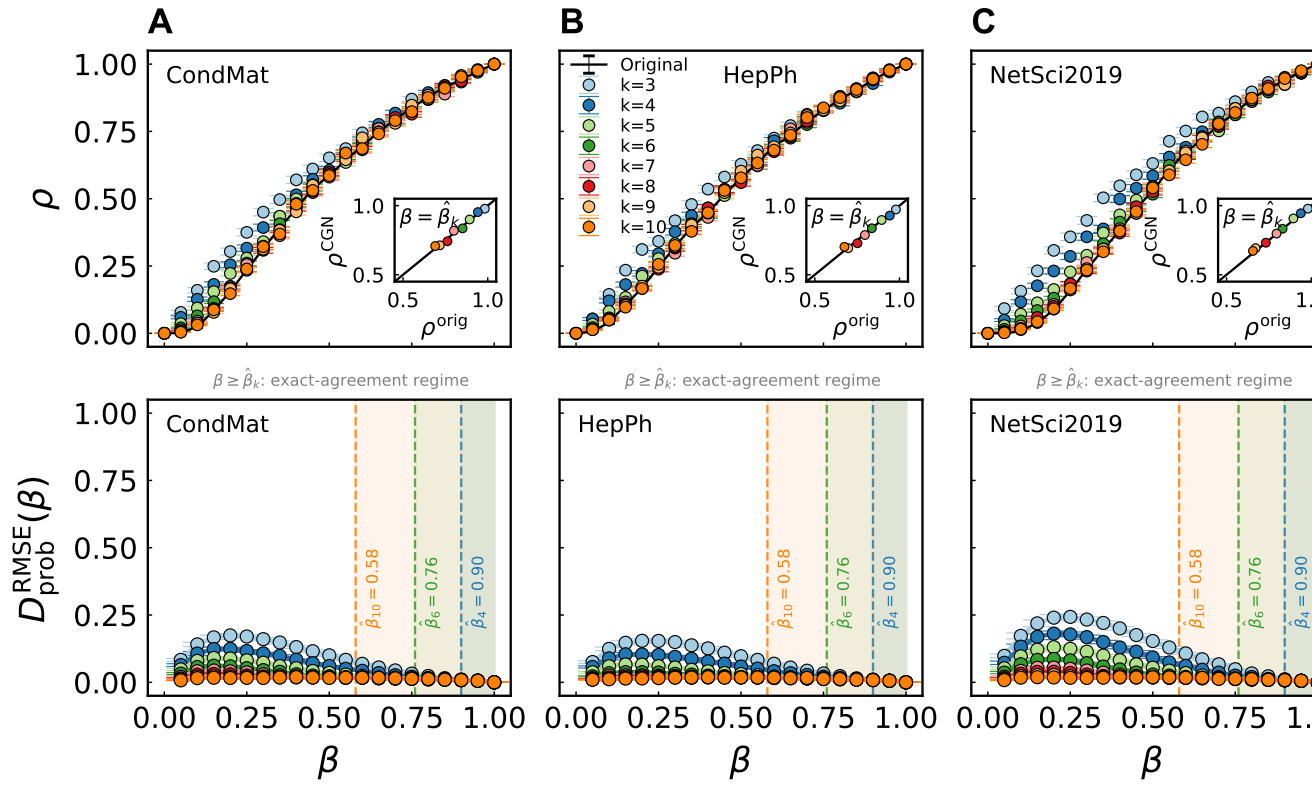

Supplementary Figure 14. **Preservation of epidemic outcomes across multiple collaboration networks.** Comparison between the original networks and their corresponding k-clique coarse-grained networks (CGNs) for three representative collaboration networks: CondMat (**A**), HepPh (**B**), and NetSci2019 (**C**). **Top panels (A-C, top)**: Final outbreak size ρ as a function of the transmission probability β for the original network (solid line) and the corresponding k-clique CGNs (k = 3-10, circle markers). Error bars represent the standard error over 1000 stochastic simulations. **Insets**: Comparison of ρ^{CGN} and ρ^{orig} at the matching rates $\beta = \hat{\beta}_k$, with $\hat{\beta}_k = (0.96, 0.90, 0.83, 0.76, 0.71, 0.66, 0.61, 0.58)$ for $k = 3, \ldots, 10$. The near-perfect agreement confirms the theoretically predicted exact-preservation regime across coarse-graining scales. **Bottom panels (A-C, bottom)**: Node-level infection-risk root-mean-square error (RMSE), $D_{\text{prob}}^{\text{RMSE}}(\beta) = \sqrt{\frac{1}{N}\sum_{i=1}^{N}(P_i^{\text{orig}}(\beta) - P_i^{\text{CGN}}(\beta))^2}$ where P_i^{orig} and P_i^{CGN} denote the infection probabilities of node i in the original network and coarse-grained networks, respectively. Each point is averaged over 100 initial seeds, with 1000 epidemic realizations per seed. For each coarse-graining scale, the microscopic error rapidly approaches zero as β reaches the corresponding matching transmission rate $\hat{\beta}_k$, demonstrating the transition from approximate to exact preservation. Similar behavior is observed across all three collaboration networks.

Generality of epidemic-outcome preservation. The exact- and approximate-preservation regimes identified in the main text are consistently reproduced across additional collaboration networks. Supplementary Figure 14 presents corresponding results for CondMat, HepPh, and NetSci2019 over coarse-graining scales ranging from $k = 3$ to 10. Across all three datasets, the final outbreak size of the coarse-grained networks closely follows that of the original networks throughout the full range of transmission probabilities. At the theoretically predicted matching rate, $\beta = \hat{\beta}_k$, the agreement becomes exact within numerical precision for every coarse-graining scale, as confirmed by the insets showing ρ^{CGN} versus ρ^{orig} collapsing onto the identity line. These observations demonstrate that the theoretically predicted matching condition identifies the exact-preservation regime across diverse collaboration networks.

The same preservation regimes are observed at the micro-

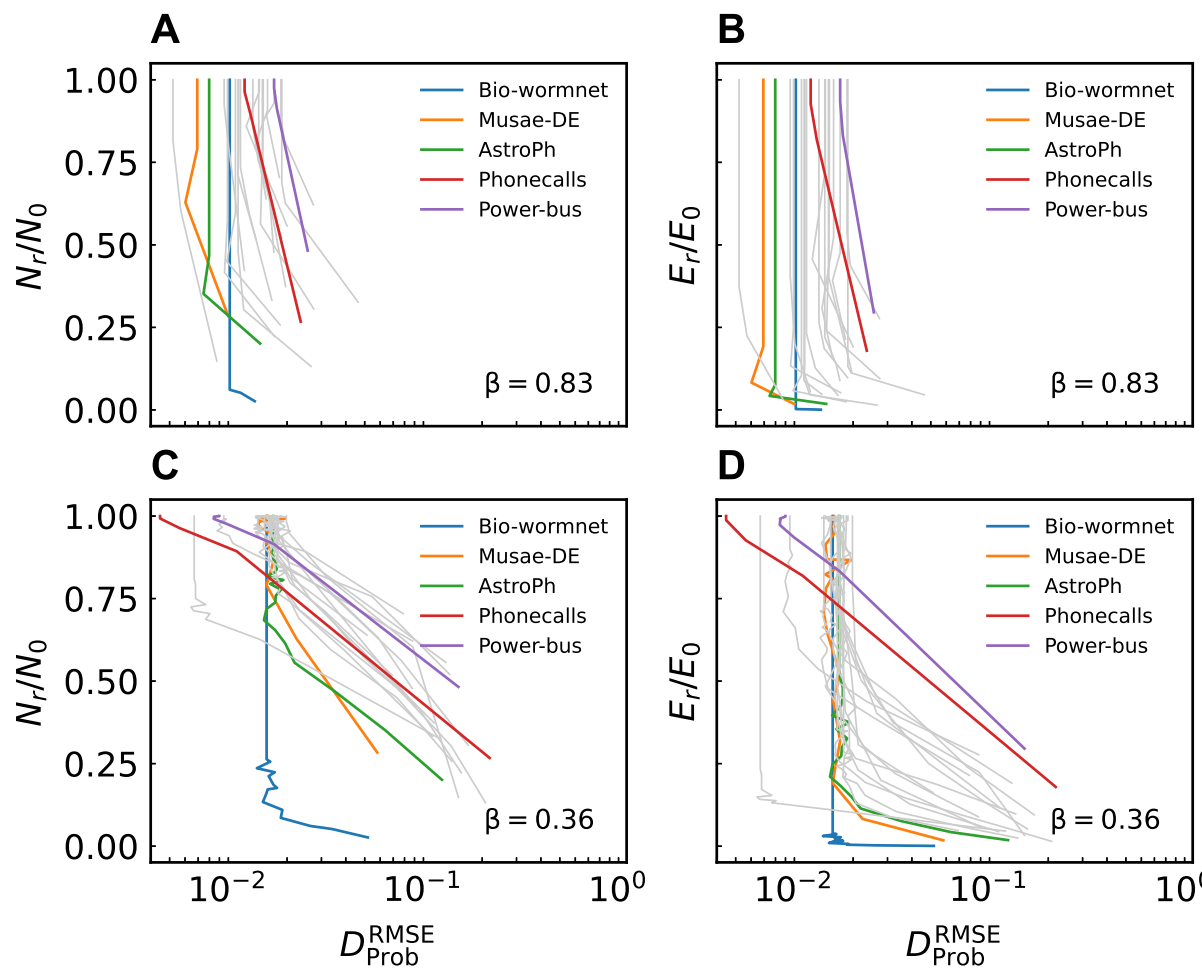

Supplementary Figure 15. **Robustness of the compression–accuracy trade-off across transmission regimes.** Compression–accuracy curves for 20 empirical networks at two transmission probabilities: $\beta = 0.83$ (**A,B**) and $\beta = 0.36$ (**C,D**). For each network, the coarse-graining scale k is varied to generate trajectories relating the retained node fraction N_r/N_0 (**A,C**) and retained edge fraction E_r/E_0 (**B,D**) to the microscopic infection-risk root-mean-square error (RMSE) $D^{\mathrm{RMSE}}_{\mathrm{prob}}$. Colored curves highlight representative best- and worst-case compression–accuracy trade-offs, while gray curves denote the remaining datasets. Across both transmission regimes, the trade-off exhibits the same characteristic structure: an initial high-fidelity plateau, where substantial structural compression is achieved with negligible microscopic error, followed by a gradual crossover in which further compression leads to progressively larger deviations. Higher transmission probabilities shift the curves toward stronger admissible compression at fixed accuracy, whereas edge compression consistently exceeds node compression throughout, reflecting the preferential removal of redundant intra-clique connectivity.

scopic level. The node-level infection-risk error, $D^{\mathrm{RMSE}}_{\mathrm{prob}}(\beta)$, decreases systematically as the transmission probability approaches the corresponding matching rate and becomes negligible within the exact-preservation regime (bottom panels of Supplementary Fig. 14). Outside the exact-preservation regime, the error increases smoothly rather than abruptly, indicating a controlled transition from exact to approximate preservation. Together, these results demonstrate that both the exact- and approximate-preservation regimes identified in the main text are consistently reproduced across additional collaboration networks, indicating that the preservation of epidemic outcomes is a general property of the ISCG framework rather than a feature specific to the GrQc network.

Robustness of the compression–accuracy trade-off. The compression–accuracy trade-off identified in the main text is robust across transmission regimes. Supplementary Figure 15 extends the analysis by comparing the compression–accuracy curves at both a higher transmission probability ($\beta = 0.83$) and a lower transmission probability ($\beta = 0.36$) across 20 empirical networks. For each transmission probability, varying the coarse-graining scale generates a characteristic compression trajectory relating the retained node and edge fractions to the microscopic infection-risk error $D^{\mathrm{RMSE}}_{\mathrm{prob}}$. Across all networks, the same qualitative behavior is consistently observed: an initial high-fidelity plateau, where substantial structural compression is achieved with negligible microscopic error, followed by a smooth crossover in which further compression produces progressively larger deviations. Although stronger transmission shifts the trade-off toward greater admissible compression at a fixed accuracy, the overall shape of the compression–accuracy relationship remains remarkably consistent. In particular, edge reduction consistently exceeds node reduction at comparable microscopic errors, indicating that ISCG preferentially removes structurally redundant intra-clique connectivity while preserving the pathways governing contagion dynamics. These results provide additional evidence that the compression–accuracy trade-off described in the main text is a robust property of the ISCG framework across both network classes and transmission regimes.

S3.5. Extension to weighted contagion dynamics

The ISCG framework naturally extends to weighted contagion dynamics without modifying the underlying coarse-graining procedure. The identification and contraction of k-cliques depend only on the network topology, while edge weights are retained through the coarse-graining process by assigning each super-edge the total weight of the corresponding inter-clique connections. This separation between structural coarse-graining and transmission dynamics allows the same coarse-grained representation to be applied directly to more general contagion models without altering the underlying reduction procedure.

To illustrate this extension, we consider the weighted US-airport2010 network, where edge weights represent heterogeneous interaction intensities. Transmission along an edge of weight w_{ij} follows the standard weighted SIR rule,

$$P_{ij} = 1 - (1 - \beta)^{w_{ij}}, \tag{S20}$$

corresponding to w_{ij} independent contact opportunities during each transmission step. The edge-weight distribution is highly heterogeneous and approximately follows a power law (Supplementary Fig. 16**A**), providing a stringent test of the framework under heterogeneous transmission dynamics.

Supplementary Figures 16**B**–**F** demonstrate that the preservation properties established for unweighted contagion remain largely unchanged in the weighted setting. Across the full range of transmission probabilities, the coarse-grained networks closely reproduce the final outbreak size and maintain small microscopic infection-risk errors over different coarse-graining scales. For a representative $k = 6$ coarse-grained network, node-level infection probabilities, mean infection times after temporal alignment, and cumulative outbreak trajectories remain in close agreement with those of the original weighted network across all transmission regimes.

Unlike in the unweighted case, heterogeneous edge weights introduce additional variability within contracted cliques, so strictly exact coarse-graining is generally not expected. Nevertheless, the resulting deviations remain small and do not substantially affect the agreement in epidemic outcomes or spatiotemporal contagion dynamics. These results show that ISCG can be extended naturally to weighted contagion dynamics while retaining high dynamical fidelity in strongly het-

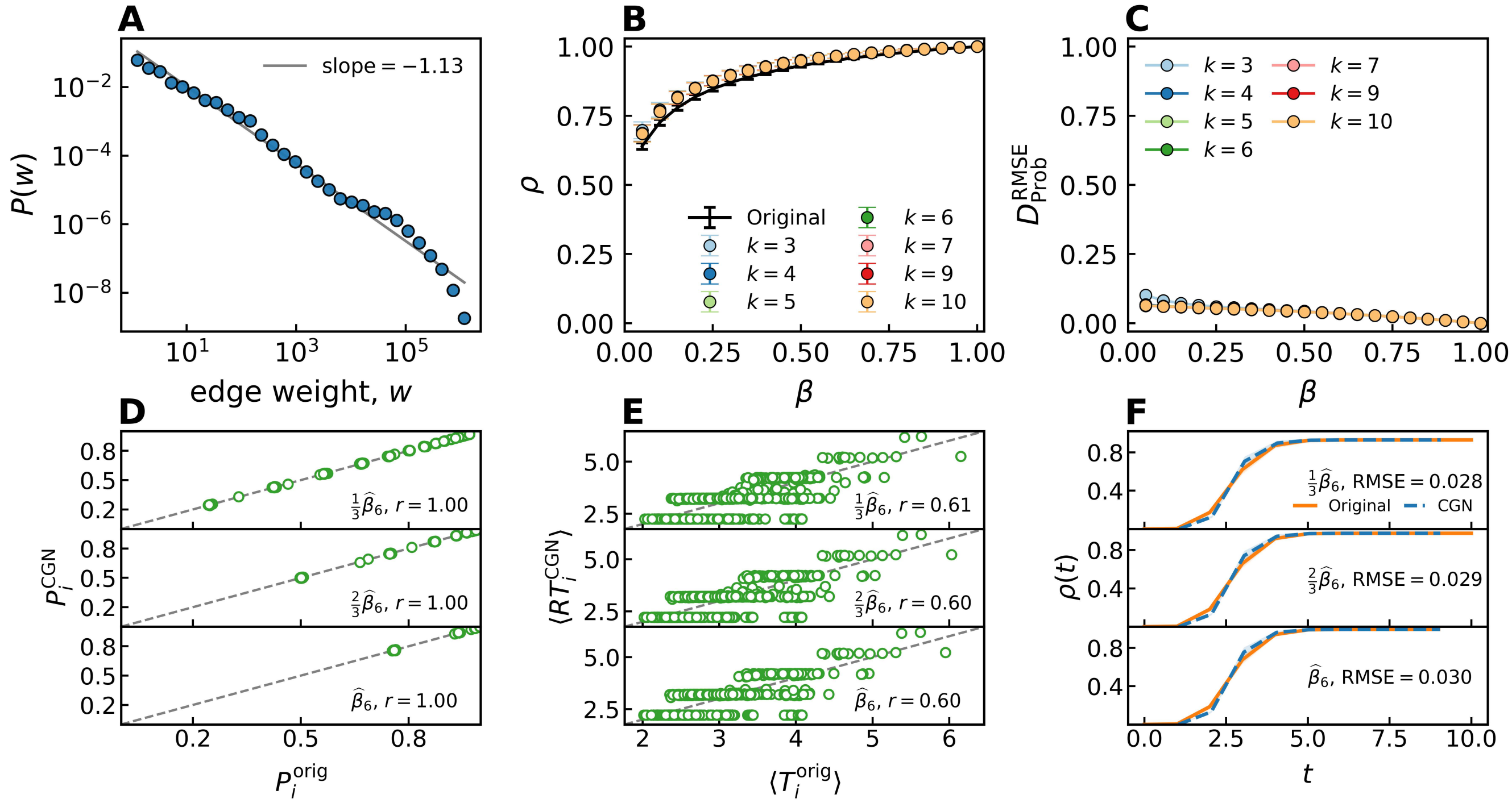

Supplementary Figure 16. **Validation of Iterative Structural Coarse-Graining (ISCG) under weighted contagion dynamics.** Validation of the ISCG framework on the weighted USairport2010 contact network, where transmission along an edge of weight w_{ij} occurs with probability $P_{ij} = 1 - (1-\beta)^{w_{ij}}$, corresponding to w_{ij} independent contact opportunities per time step. Unlike the unweighted susceptible-infected-recovered (SIR) model considered throughout the main text, this formulation introduces heterogeneous transmission probabilities determined by edge weights. **A**, Distribution of edge weights in the USairport2010 network, exhibiting a heterogeneous power-law form, $P(w) \sim w^{-1.13}$, estimated by maximum likelihood. **B,C**, Preservation of epidemic outcomes across coarse-graining scales. Panel **B** compares the final outbreak size ρ of the original and k-clique coarse-grained networks as a function of the transmission probability β, while panel **C** reports the corresponding node-level infection-risk root-mean-square error (RMSE) $D^{\mathrm{RMSE}}_{\mathrm{prob}}$. Each point represents an average over 100 randomly selected outbreak seeds, with 1000 independent epidemic realizations per seed. **D-F**, Preservation of spatiotemporal contagion dynamics for the $k = 6$ clique CGN at $\beta = \frac{\hat{\beta}_6}{3} = 0.253$, $\frac{2\hat{\beta}_6}{3} = 0.507$, and $\hat{\beta}_6 = 0.760$. Columns from left to right compare node-level infection probabilities, mean infection times, and cumulative outbreak trajectories $\rho(t)$. Mean infection times and outbreak trajectories are shown after applying the global temporal shift, $\Delta T = \langle T^{\mathrm{orig}}\rangle - \langle T^{\mathrm{CGN}}\rangle$. Shaded regions in the **F** denote the standard error of the mean over 100 randomly selected outbreak seeds, each averaged over 500 independent epidemic realizations. Panels **D-F** were computed from 100 randomly selected outbreak seeds, each averaged over 500 independent epidemic realizations; shaded regions in panel **F** denote the standard error across outbreak seeds. Across all transmission regimes, the weighted coarse-grained networks accurately reproduce both epidemic outcomes and spatiotemporal contagion dynamics despite the strong heterogeneity of edge weights, demonstrating that the ISCG framework naturally extends beyond unweighted contagion models.

erogeneous contact networks. A theoretical characterization of exact-preservation conditions for weighted or directed networks remains an important direction for future work.

S3.6. Applications of the ISCG framework

ISCG-IM method. Supplementary Figure 17**A** evaluates the performance of the ISCG-based influence maximization (ISCG-IM) method across a range of k-clique CGNs ($k = 3$ to 10). The results show that the final infected node density (ρ) remains consistently high across all scales, indicating that ISCG-IM is robust to the choice of k. This scale-invariance allows the method to adapt effectively to networks with diverse resolutions and structural properties.

Mechanisms behind ISCG-IM effectiveness. The ISCG-IM method's superior performance stems from its integration of macroscopic and microscopic perspectives. At the macroscopic level, k-clique CGNs highlight structural cores—densely connected super-nodes and their interconnections—enabling the identification of high-impact regions. This facilitates seed selection based on global structural significance. At the microscopic level, seed selection is refined within each super-node to prevent redundancy and ensure maximal coverage. Supplementary Figure 17**B** demonstrates that when multiple seeds are placed within the same super-node, contagion performance exhibits diminishing returns, particularly at higher β. By distributing seeds across distinct super-nodes, ISCG-IM mitigates overlap and amplifies spreading efficiency.

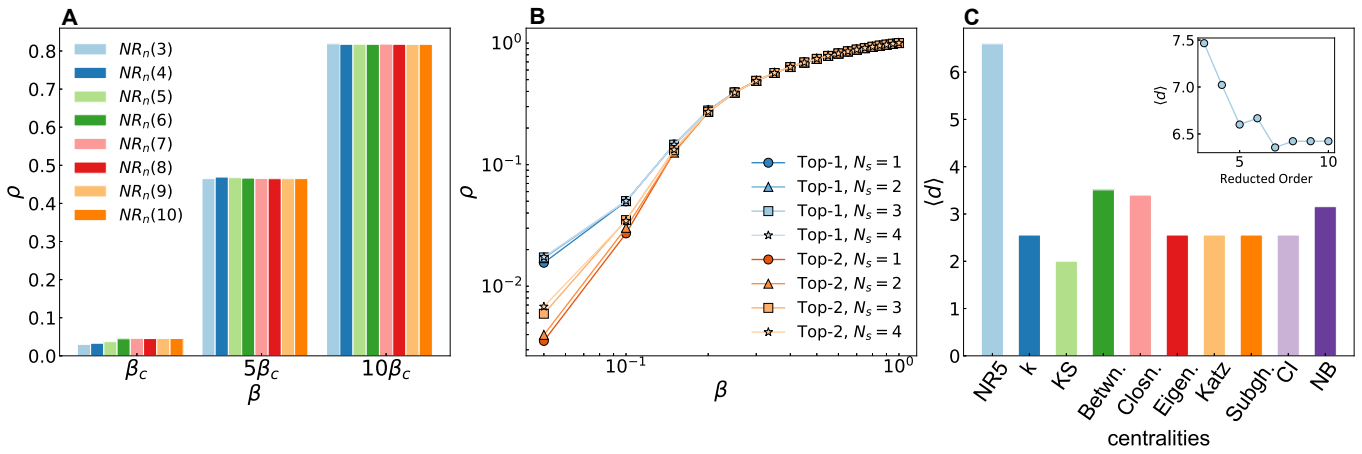

Supplementary Figure 17. **Robustness and efficiency of the Iterative Structural Coarse-Graining-based influence maximization (ISCG-IM) method. A**, Final infection density ρ as a function of transmission probability β, measured across k-clique coarse-grained networks (CGNs) ($k = 3$ to 10), illustrating the effect of structural resolution on spreading dynamics. Simulations are initialized using the top $n = 10$ seed nodes selected by the ISCG-IM method. The results demonstrate that ISCG-IM maintains consistently high performance across coarse-graining scales, indicating strong robustness. **B**, Final infection density ρ for contagion processes initiated by $N_s = 1$ to 4 seeds randomly selected within the same super-node. Results are shown for the two highest-weight super-nodes in the 5-clique CGN, demonstrating the internal redundancy and robustness of seed placement under the ISCG-IM method. **C**, Average shortest path length $\langle d \rangle$ among the top 10 selected seeds using various methods. Inset: $\langle d \rangle$ for ISCG-IM across different k-clique CGNs. These metrics reveal that ISCG-IM strategically identifies seeds with wide topological reach, underpinning its superior spreading effectiveness on multi-scale network representations. All results are based on simulations on the GrQc network.

Within each super-node, a lightweight heuristic such as node degree is used to select the most influential node, capitalizing on local structure. This dual-scale strategy—global distribution via CGNs and local optimization via centrality—enables ISCG-IM to identify seed sets that maximize both reach and efficiency.

Synergy among selected seeds. To quantify the diversity and reach of selected seeds, Supplementary Figure 17**C** presents the average shortest path length between all pairs of seeds chosen by different methods. ISCG-IM selects seeds with significantly longer average distances, ensuring spatial dispersion and minimizing redundancy. The inset further analyzes how the choice of k influences seed spread: as k increases, the average shortest path decreases, indicating that seeds become more localized within tighter communities. This reveals a trade-off between coarse-graining level and seed diversity, suggesting the importance of tuning k to achieve optimal coverage.

The ISCG-IM method's ability to fuse macroscopic structural insight with microscopic precision underpins its effectiveness in influence maximization. By exploiting multi-scale properties of CGNs, it outperforms traditional adaptive centrality-based methods, offering superior adaptability across network resolutions and enhanced scalability for large-scale applications. These results highlight the broad potential of ISCG-IM for applications in contagion modeling, strategic seeding, and network-based interventions.

ISCG-EI method. Supplementary Figure 18**A** evaluates the robustness of the ISCG-based edge immunization (ISCG-EI) method across a range of k-clique CGNs ($k = 3$ to 10).

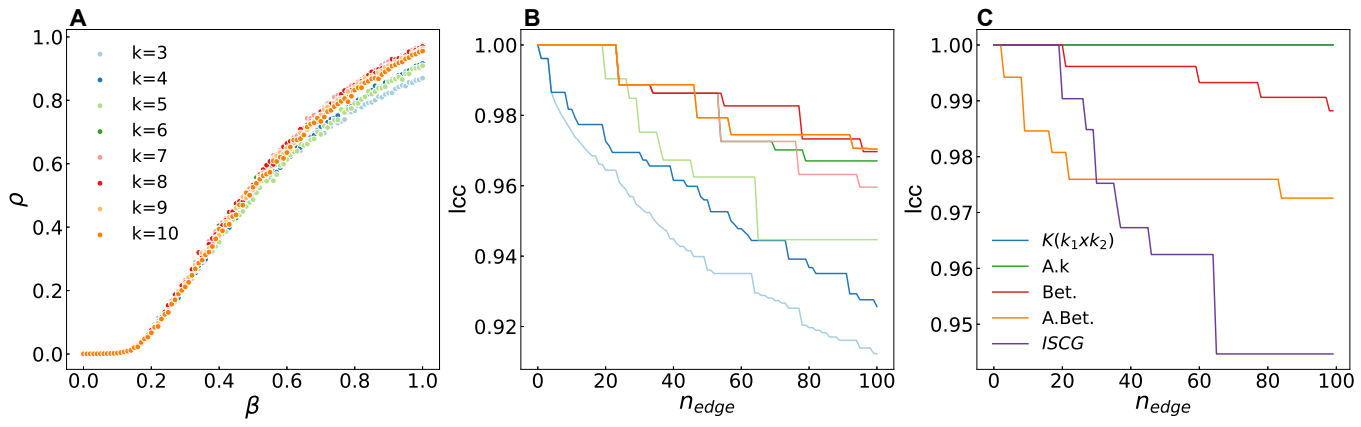

Supplementary Figure 18. **Robustness and efficiency of Iterative Structural Coarse-Graining-based edge immunization (ISCG-EI). A**, Final infection density ρ under the ISCG-EI strategy across multiple k-clique coarse-grained networks (CGNs) ($k = 3$ to 10), with 50 edges immunized in each case. The results quantify the method's effectiveness in suppressing contagion through targeted edge removal. **B**, Relative size of the largest connected component (LCC) following sequential removal of n_{edge} edges identified by ISCG-EI across different CGNs. The decline in LCC highlights the structural impact of immunization across scales. **C**, Comparison of LCC size under ISCG-EI ($k = 5$) and alternative edge centrality-based strategies. ISCG-EI exhibits superior performance in fragmenting the network. All analyses are performed on the GrQc network, demonstrating the efficacy of ISCG-EI in structurally disrupting transmission pathways via edge-based interventions.

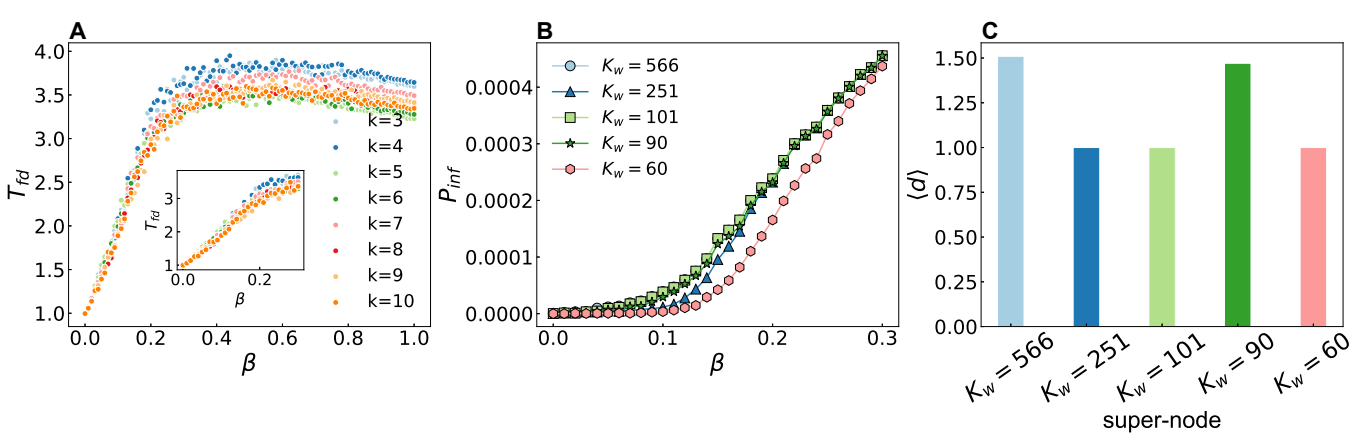

Supplementary Figure 19. **Iterative Structural Coarse-Graining-based sentinel surveillance (ISCG-SS) enables efficient early detection and structurally grounded monitoring. A**, Time to first detection (T_{fd}) of contagion signals using the top 10 sentinel nodes selected by the ISCG-SS method across various k-clique coarse-grained networks (CGNs) ($k = 3$ to 10). *Inset*: Expanded view at lower transmission probabilities β, highlighting detection sensitivity in weak-spreading regimes. **B**, Infection probability P_{inf} of the top five super-nodes in the 10-clique CGN, shown as a function of β. Super-nodes are ranked by their weighted degree K_w (i.e., total incident edge weight), revealing a strong correlation between structural prominence and contagion exposure. **C**, Average shortest path length within individual super-nodes, reflecting internal structural compactness—a critical feature enabling fast intra-group spread and reliable detection. All simulations are performed on the *GrQc* network, illustrating how ISCG-SS identifies structurally strategic locations for timely and robust outbreak monitoring across coarse-grained representations.

The final infected node density (ρ) remains consistent across all k-values, demonstrating the method's adaptability to different coarse-grained scales. Interestingly, slightly better performance is observed at lower k-values (e.g., $k = 3$), suggesting that coarser representations help identify broader structural features critical for effective immunization.

Structural impact on connectivity. To uncover the mechanisms behind ISCG-EI's effectiveness, Supplementary Figure 18**B** analyzes the proportion of the largest connected component (LCC) remaining after removing a fixed number of edges identified by ISCG-EI across various k-clique CGNs. At coarser scales (e.g., $k = 3$), the LCC shrinks dramatically, indicating that the selected edges play pivotal roles in maintaining global network connectivity. This illustrates ISCG-EI's ability to target structurally critical links that act as bridges between densely connected regions. By removing such links, the method effectively fragments the network and suppresses large-scale propagation.

Comparison with alternative methods. Supplementary Figure 18**C** compares ISCG-EI (at $k = 5$) with traditional edge-based centrality strategies, including adaptive betweenness and degree-based heuristics, using LCC size as a performance metric. ISCG-EI consistently outperforms these baselines, achieving a greater reduction in network connectivity. This advantage arises from its capacity to identify edges whose removal initiates cascading structural breakdowns. In contrast, other methods often focus on local or high-traffic edges, which may not effectively disrupt backbone structures in networks with clustered topologies.

By leveraging the multi-scale structural insights provided by k-clique CGNs, ISCG-EI identifies edges that are essential for maintaining both network connectivity and contagion transmission. At lower k, the method captures high-level structural dependencies that guide effective immunization strategies. This adaptability across scales makes ISCG-EI a robust and versatile tool for controlling contagion spread in diverse networked systems.

ISCG-SS method. Supplementary Figure 19**A** evaluates the performance of the ISCG-based sentinel surveillance method (ISCG-SS) across a range of k-clique CGNs ($k = 3$ to 10) using the *GrQc* network. As expected, the time to first detection T_{fd} decreases with increasing transmission probability β, and performance peaks at intermediate coarse-graining scales ($k = 5$ and $k = 6$), where the lowest T_{fd} values are observed at high β. This suggests that ISCG-SS achieves an optimal balance between structural resolution and dynamic accuracy at these scales. At lower β (inset), differences across k are negligible, indicating that ISCG-SS remains robust even in low-transmission regimes. This stability across both scale and dynamics underscores the method's adaptability across diverse network and epidemic conditions.

Mechanisms behind ISCG-SS effectiveness. To understand the drivers of ISCG-SS's performance, Supplementary Figures 19**B** and **C** analyze two key structural attributes of the selected sentinel nodes. Supplementary Figure 19**B** shows the infection probability P_{inf} of the top five super-nodes identified in the 10-clique CGN as a function of β. A clear positive correlation is observed between a super-node's weighted degree (K_w) and its likelihood of infection. Super-nodes with higher K_w are more exposed to incoming contagion due to their high connectivity, making them ideal candidates for early outbreak detection. Supplementary Figure 19**C** examines the average shortest path length $\langle d \rangle$ within individual super-nodes. The results show little variation across different K_w values, indicating consistently tight internal structure. This ensures that once a sentinel node within a super-node becomes infected, the contagion spreads quickly to the rest of the group. Together with the findings from Supplementary Fig. 19**B**, these results demonstrate that ISCG-SS effectively combines macroscopic (inter-super-node exposure) and microscopic (intra-super-node compactness) insights to optimize detection.

The ISCG-SS method provides a robust and scalable strategy for sentinel surveillance by leveraging multi-scale structural features of CGNs. At higher transmission probabilities, intermediate k-values ($k = 5, 6$) offer the best trade-off between coarse-graining and detection speed. The method's ability to prioritize sentinel nodes within super-nodes that are both highly exposed and internally cohesive ensures rapid and reliable outbreak detection. These results establish ISCG-SS as a powerful and generalizable tool for enhancing early-warning systems in a wide range of networked spreading processes.

REFERENCES

1. R. Pastor-Satorras, C. Castellano, P. Van Mieghem, A. Vespignani, Epidemic processes in complex networks. *Rev. Mod. Phys.* **87**, 925–979 (2015).

2. Z.-K. Zhang, C. Liu, X.-X. Zhan, X. Lu, C.-X. Zhang, Y.-C. Zhang, Dynamics of information diffusion and its applications on complex networks. *Phys. Rep.* **651**, 1–34 (2016).

3. F. Battiston, G. Cencetti, I. Iacopini, V. Latora, M. Lucas, A. Patania, J.-G. Young, G. Petri, Networks beyond pairwise interactions: Structure and dynamics. *Phys. Rep.* **874**, 1–92 (2020).

4. R. Pastor-Satorras, A. Vespignani, Epidemic spreading in scale-free networks. *Phys. Rev. Lett.* **86**, 3200–3203 (2001).

5. V. Colizza, A. Barrat, M. Barthélemy, A. Vespignani, The role of the airline transportation network in the prediction and predictability of global epidemics. *Proc. Natl. Acad. Sci. U.S.A.* **103**, 2015–2020 (2006).

6. J. L. Iribarren, E. Moro, Impact of human activity patterns on the dynamics of information diffusion. *Phys. Rev. Lett.* **103**, 038702 (2009).

7. J. T. Davis, N. Perra, Q. Zhang, Y. Moreno, A. Vespignani, Phase transitions in information spreading on structured populations. *Nat. Phys.* **16**, 590–596 (2020).

8. C. Nardini, B. Kozma, A. Barrat, Who's talking first? Consensus or lack thereof in coevolving opinion formation models. *Phys. Rev. Lett.* **100**, 158701 (2008).

9. H. Schawe, L. Hernández, Higher order interactions destroy phase transitions in Deffuant opinion dynamics model. *Commun. Phys.* **5**, 32 (2022).

10. D. Brockmann, D. Helbing, The hidden geometry of complex, network-driven contagion phenomena. *Science* **342**, 1337–1342 (2013).

11. G. Burgio, A. Arenas, S. Gómez, J. T. Matamalas, Network clique cover approximation to analyze complex contagions through group interactions. *Commun. Phys.* **4**, 111 (2021).

12. Y. Moreno, R. Pastor-Satorras, A. Vespignani, Epidemic outbreaks in complex heterogeneous networks. *Eur. Phys. J. B* **26**, 521–529 (2002).

13. C. Castellano, R. Pastor-Satorras, Thresholds for epidemic spreading in networks. *Phys. Rev. Lett.* **105**, 218701 (2010).

14. M. E. Newman, Spread of epidemic disease on networks. *Phys. Rev. E* **66**, 016128 (2002).

15. E. Volz, SIR dynamics in random networks with heterogeneous connectivity. *J. Math. Biol.* **56**, 293–310 (2008).

16. B. Karrer, M. E. Newman, Message passing approach for general epidemic models. *Phys. Rev. E* **82**, 016101 (2010).

17. A. Y. Lokhov, M. Mézard, L. Zdeborová, Dynamic message-passing equations for models with unidirectional dynamics. *Phys. Rev. E* **91**, 012811 (2015).

18. G. T. Cantwell, M. E. Newman, Message passing on networks with loops. *Proc. Natl. Acad. Sci. U.S.A.* **116**, 23398–23403 (2019).

19. M. Girvan, M. E. Newman, Community structure in social and biological networks. *Proc. Natl. Acad. Sci. U.S.A.* **99**, 7821–7826 (2002).

20. C. Song, S. Havlin, H. A. Makse, Self-similarity of complex networks. *Nature* **433**, 392–395 (2005).

21. M. Sales-Pardo, R. Guimera, A. A. Moreira, L. A. N. Amaral, Extracting the hierarchical organization of complex systems. *Proc. Natl. Acad. Sci. U.S.A.* **104**, 15224–15229 (2007).

22. M. A. Serrano, M. Boguñá, A. Vespignani, Extracting the multiscale backbone of complex weighted networks. *Proc. Natl. Acad. Sci. U.S.A.* **106**, 6483–6488 (2009).

23. S. Xu, P. Wang, "Coarse graining of complex networks: A k-means clustering approach," in *Proceedings of the 28th Chinese Control and Decision Conference, CCDC 2016* (IEEE, 2016), pp. 4113–4118.

24. G. García-Pérez, M. Boguñá, M. Serrano, Multiscale unfolding of real networks by geometric renormalization. *Nat. Phys.* **14**, 583–589 (2018).

25. D. Grady, C. Thiemann, D. Brockmann, Robust classification of salient links in complex networks. *Nat. Commun.* **3**, 864 (2012).

26. L. M. Shekhtman, J. P. Bagrow, D. Brockmann, Robustness of skeletons and salient features in networks. *J. Complex Netw.* **2**, 110–120 (2014).

27. A. Mercier, S. Scarpino, C. Moore, Effective resistance against pandemics: Mobility network sparsification for high-fidelity epidemic simulations. *PLoS Comput. Biol.* **18**, e1010650 (2022).

28. T. P. Peixoto, L. Gauvin, Change points, memory and epidemic spreading in temporal networks. *Sci. Rep.* **8**, 15511 (2018).

29. M. De Domenico, V. Nicosia, A. Arenas, V. Latora, Structural reducibility of multilayer networks. *Nat. Commun.* **6**, 6864 (2015).

30. D. Gfeller, P. De Los Rios, Spectral coarse graining of complex networks. *Phys. Rev. Lett.* **99**, 038701 (2007).

31. D. Gfeller, P. De Los Rios, Spectral coarse graining and synchronization in oscillator networks. *Phys. Rev. Lett.* **100**, 174104 (2008).

32. V. Thibeault, G. St-Onge, L. J. Dubé, P. Desrosiers, Threefold way to the dimension reduction of dynamics on networks: An application to synchronization. *Phys. Rev. Res.* **2**, 043215 (2020).

33. E. Laurence, N. Doyon, L. J. Dubé, P. Desrosiers, Spectral dimension reduction of complex dynamical networks. *Phys. Rev. X* **9**, 011042 (2019).

34. S. Ghosh, P. Khanra, P. Kundu, P. Ji, D. Ghosh, C. Hens, Dimension reduction in higher-order contagious phenomena. *Chaos* **33**, 053117 (2023).

35. J. Gao, Intrinsic simplicity of complex systems. *Nat. Phys.* **20**, 184–185 (2024).

36. A. J. Allen, C. Moore, L. Hébert-Dufresne, Compressing the chronology of a temporal network with graph commutators. *Phys. Rev. Lett.* **132**, 077402 (2024).

37. V. Thibeault, A. Allard, P. Desrosiers, The low-rank hypothesis of complex systems. *Nat. Phys.* **20**, 294–302 (2024).

38. Y. Chen, G. Paul, S. Havlin, F. Liljeros, H. E. Stanley, Finding a better immunization strategy. *Phys. Rev. Lett.* **101**, 058701 (2008).

39. C. M. Schneider, T. Mihaljev, S. Havlin, H. J. Herrmann, Suppressing epidemics with a limited amount of immunization units. *Phys. Rev. E* **84**, 061911 (2011).

40. M. Kitsak, L. K. Gallos, S. Havlin, F. Liljeros, L. Muchnik, H. E. Stanley, H. A. Makse, Identification of influential spreaders in complex networks. *Nat. Phys.* **6**, 888–893 (2010).

41. L. Xue, P. Zhang, A. Zeng, Maximizing spreading in complex networks with risk in node activation. *Inf. Sci.* **586**, 1–23 (2022).

42. R. A. Rossi, N. K. Ahmed, "The network data repository with interactive graph analytics and visualization," in *Proceedings of the 29th AAAI Conference on Artificial Intelligence* (AAAI Press, 2015).

43. T. Yabe, K. Tsubouchi, T. Shimizu, Y. Sekimoto, K. Sezaki, E. Moro, A. Pentland, YJMob100K: City scale and longitudinal dataset of anonymized human mobility trajectories. *Sci. Data* **11**, 397 (2024).

44. P. Holme, Three faces of node importance in network epidemiology: Exact results for small graphs. *Phys. Rev. E* **96**, 062305 (2017).

45. W. Chen, Y. Wang, S. Yang, "Efficient influence maximization in social networks," in *Proceedings of the 15th ACM SIGKDD International Conference on Knowledge Discovery Data Mining* (Association for Computing Machinery, 2009), pp. 199–208.

46. F. Morone, H. A. Makse, Influence maximization in complex networks through optimal percolation. *Nature* **524**, 65–68 (2015).

47. P. Bonacich, Some unique properties of eigenvector centrality. *Soc. Netw.* **29**, 555–564 (2007).

48. J. Zhan, S. Gurung, S. P. K. Parsa, Identification of top-K nodes in large networks using Katz centrality. *J. Big Data* **4**, 16 (2017).

49. E. Estrada, J. A. Rodriguez-Velazquez, Subgraph centrality in complex networks. *Phys. Rev. E* **71**, 056103 (2005).

50. T. Martin, X. Zhang, M. E. Newman, Localization and centrality in networks. *Phys. Rev. E* **90**, 052808 (2014).

51. E. Colman, P. Holme, H. Sayama, C. Gershenson, Efficient sentinel surveillance strategies for preventing epidemics on networks. *PLoS Comput. Biol.* **15**, e1007517 (2019).

52. J. L. Herrera, R. Srinivasan, J. S. Brownstein, A. P. Galvani, L. A. Meyers, Disease surveillance on complex social networks. *PLoS Comput. Biol.* **12**, e1004928 (2016).

53. G. St-Onge, J.-G. Young, L. Hébert-Dufresne, L. J. Dubé, Efficient sampling of spreading processes on complex networks using a composition and rejection algorithm. *Comput. Phys. Commun.* **240**, 30–37 (2019).

54. J. C. Miller, Epidemics on networks with large initial conditions or changing structure. *PLOS ONE* **9**, e101421 (2014).

55. E. Kenah, J. M. Robins, Second look at the spread of epidemics on networks. *Phys. Rev. E* **76**, 036113 (2007).

56. I. Iacopini, G. Petri, A. Barrat, V. Latora, Simplicial models of social contagion. *Nat. Commun.* **10**, 2485 (2019).

57. F. Battiston, E. Amico, A. Barrat, G. Bianconi, G. Ferraz de Arruda, B. Franceschiello, I. Iacopini, S. Kéfi, V. Latora, Y. Moreno, M. M. Murray, T. P. Peixoto, F. Vaccarino, G. Petri, The physics of higher-order in teractions in complex systems. *Nat. Phys.* **17**, 1093–1098 (2021).

58. G. Ferraz de Arruda, A. Aleta, Y. Moreno, Contagion dynamics on higher-order networks. *Nat. Rev. Phys.* **6**, 468–482 (2024).

59. Y. Zhang, M. Lucas, F. Battiston, Higher-order interactions shape collective dynamics differently in hypergraphs and simplicial complexes. *Nat. Commun.* **14**, 1605 (2023).

60. B. Gross, D. Vaknin, S. V. Buldyrev, S. Havlin, Two transitions in spatial modular networks. *New J. Phys.* **22**, 053002 (2020).

61. U. Alvarez-Rodriguez, F. Battiston, G. F. de Arruda, Y. Moreno, M. Perc, V. Latora, Evolutionary dynamics of higher-order interactions in social networks. *Nat. Hum. Behav.* **5**, 586–595 (2021).

62. F. Radicchi, Predicting percolation thresholds in networks. *Phys. Rev. E* **91**, 010801 (2015).

63. J. Leskovec, A. Krevl, SNAP Datasets: Stanford large network dataset collection (2014); http://snap.stanford.edu/data.

64. A.-L. Barabási, *Network Science* (Cambridge Univ. Press, 2016).

65. Network Repository, Network Repository. An Interactive Scientific Network Data Repository, https://networkrepository.com/.

66. B. Rozemberczki, Datasets, GitHub, https://github.com/benedekrozemberczki/datasets.

67. Stanford University, Stanford Network Analysis Project, http://snap.stanford.edu/data/.

68. ICON, Index of Complex Networks, https://icon.colorado.edu/#!/networks.

69. A.-L. Barabási, Network Science: Data (2016); http://networksciencebook.com/translations/en/resources/data.html.
